# A structural variation reference for medical and population genetics

**DOI:** 10.1038/s41586-020-2287-8

**Published:** 2020-05-27

**Authors:** Ryan L. Collins, Harrison Brand, Konrad J. Karczewski, Xuefang Zhao, Jessica Alföldi, Laurent C. Francioli, Amit V. Khera, Chelsea Lowther, Laura D. Gauthier, Harold Wang, Nicholas A. Watts, Matthew Solomonson, Anne O’Donnell-Luria, Alexander Baumann, Ruchi Munshi, Mark Walker, Christopher W. Whelan, Yongqing Huang, Ted Brookings, Ted Sharpe, Matthew R. Stone, Elise Valkanas, Jack Fu, Grace Tiao, Kristen M. Laricchia, Valentin Ruano-Rubio, Christine Stevens, Namrata Gupta, Caroline Cusick, Lauren Margolin, Genome Aggregation Database Production Team, Jessica Alföldi, Irina M. Armean, Eric Banks, Louis Bergelson, Kristian Cibulskis, Ryan L. Collins, Kristen M. Connolly, Miguel Covarrubias, Beryl Cummings, Mark J. Daly, Stacey Donnelly, Yossi Farjoun, Steven Ferriera, Laurent Francioli, Stacey Gabriel, Laura D. Gauthier, Jeff Gentry, Namrata Gupta, Thibault Jeandet, Diane Kaplan, Konrad J. Karczewski, Kristen M. Laricchia, Christopher Llanwarne, Eric V. Minikel, Ruchi Munshi, Benjamin M. Neale, Sam Novod, Anne H. O’Donnell-Luria, Nikelle Petrillo, Timothy Poterba, David Roazen, Valentin Ruano-Rubio, Andrea Saltzman, Kaitlin E. Samocha, Molly Schleicher, Cotton Seed, Matthew Solomonson, Jose Soto, Grace Tiao, Kathleen Tibbetts, Charlotte Tolonen, Christopher Vittal, Gordon Wade, Arcturus Wang, Qingbo Wang, James S. Ware, Nicholas A. Watts, Ben Weisburd, Nicola Whiffin, Genome Aggregation Database Consortium, Carlos A. Aguilar Salinas, Tariq Ahmad, Christine M. Albert, Diego Ardissino, Gil Atzmon, John Barnard, Laurent Beaugerie, Emelia J. Benjamin, Michael Boehnke, Lori L. Bonnycastle, Erwin P. Bottinger, Donald W. Bowden, Matthew J. Bown, John C. Chambers, Juliana C. Chan, Daniel Chasman, Judy Cho, Mina K. Chung, Bruce Cohen, Adolfo Correa, Dana Dabelea, Mark J. Daly, Dawood Darbar, Ravindranath Duggirala, Josée Dupuis, Patrick T. Ellinor, Roberto Elosua, Jeanette Erdmann, Tõnu Esko, Martti Färkkilä, Jose Florez, Andre Franke, Gad Getz, Benjamin Glaser, Stephen J. Glatt, David Goldstein, Clicerio Gonzalez, Leif Groop, Christopher Haiman, Craig Hanis, Matthew Harms, Mikko Hiltunen, Matti M. Holi, Christina M. Hultman, Mikko Kallela, Jaakko Kaprio, Sekar Kathiresan, Bong-Jo Kim, Young Jin Kim, George Kirov, Jaspal Kooner, Seppo Koskinen, Harlan M. Krumholz, Subra Kugathasan, Soo Heon Kwak, Markku Laakso, Terho Lehtimäki, Ruth J. F. Loos, Steven A. Lubitz, Ronald C. W. Ma, Daniel G. MacArthur, Jaume Marrugat, Kari M. Mattila, Steven McCarroll, Mark I. McCarthy, Dermot McGovern, Ruth McPherson, James B. Meigs, Olle Melander, Andres Metspalu, Benjamin M. Neale, Peter M. Nilsson, Michael C. O’Donovan, Dost Ongur, Lorena Orozco, Michael J. Owen, Colin N. A. Palmer, Aarno Palotie, Kyong Soo Park, Carlos Pato, Ann E. Pulver, Nazneen Rahman, Anne M. Remes, John D. Rioux, Samuli Ripatti, Dan M. Roden, Danish Saleheen, Veikko Salomaa, Nilesh J. Samani, Jeremiah Scharf, Heribert Schunkert, Moore B. Shoemaker, Pamela Sklar, Hilkka Soininen, Harry Sokol, Tim Spector, Patrick F. Sullivan, Jaana Suvisaari, E. Shyong Tai, Yik Ying Teo, Tuomi Tiinamaija, Ming Tsuang, Dan Turner, Teresa Tusie-Luna, Erkki Vartiainen, Marquis P. Vawter, James S. Ware, Hugh Watkins, Rinse K. Weersma, Maija Wessman, James G. Wilson, Ramnik J. Xavier, Kent D. Taylor, Henry J. Lin, Stephen S. Rich, Wendy S. Post, Yii-Der Ida Chen, Jerome I. Rotter, Chad Nusbaum, Anthony Philippakis, Eric Lander, Stacey Gabriel, Benjamin M. Neale, Sekar Kathiresan, Mark J. Daly, Eric Banks, Daniel G. MacArthur, Michael E. Talkowski

**Affiliations:** 1 Program in Medical and Population Genetics, Broad Institute of MIT and Harvard, Cambridge, MA USA; 2 Center for Genomic Medicine, Massachusetts General Hospital, Boston, MA USA; 3 Division of Medical Sciences, Harvard Medical School, Boston, MA USA; 4 Department of Neurology, Massachusetts General Hospital and Harvard Medical School, Boston, MA USA; 5 Analytical and Translational Genetics Unit, Massachusetts General Hospital, Boston, MA USA; 6 Department of Medicine, Harvard Medical School, Boston, MA USA; 7 Data Science Platform, Broad Institute of MIT and Harvard, Cambridge, MA USA; 8 The Institute for Translational Genomics and Population Sciences, Department of Pediatrics, Los Angeles Biomedical Research Institute at Harbor-UCLA Medical Center, Torrance, CA USA; 9 Center for Public Health Genomics, University of Virginia, Charlottesville, VA USA; 10 Johns Hopkins University School of Medicine, Baltimore, MD USA; 11 Department of Systems Biology, Harvard Medical School, Boston, MA USA; 12 Department of Biology, MIT, Cambridge, MA USA; 13 Stanley Center for Psychiatric Research, Broad Institute of MIT and Harvard, Cambridge, MA USA; 14 Division of Cardiology, Massachusetts General Hospital, Boston, MA USA; 155 Present Address: Cellarity Inc., Cambridge, MA USA; 156 Present Address: Centre for Population Genomics, Garvan Institute of Medical Research, and UNSW Sydney, Sydney, Australia; 157 Present Address: Centre for Population Genomics, Murdoch Children’s Research Institute, Melbourne, Australia; 15 Analytic and Translational Genetics Unit, Massachusetts General Hospital, Boston, MA USA; 16 European Molecular Biology Laboratory, European Bioinformatics Institute, Wellcome Genome Campus, Hinxton, Cambridge, UK; 17 Genomics Platform, Broad Institute of MIT and Harvard, Cambridge, MA USA; 18 Broad Genomics, Broad Institute of MIT and Harvard, Cambridge, MA USA; 19 Division of Genetics and Genomics, Boston Children’s Hospital, Boston, MA USA; 20 Department of Pediatrics, Harvard Medical School, Boston, MA USA; 21 Wellcome Sanger Institute, Wellcome Genome Campus, Hinxton, Cambridge, UK; 22 National Heart & Lung Institute and MRC London Institute of Medical Sciences, Imperial College London, London, UK; 23 Cardiovascular Research Centre, Royal Brompton & Harefield Hospitals NHS Trust, London, UK; 24 Unidad de Investigacion de Enfermedades Metabolicas, Instituto Nacional de Ciencias Medicas y Nutricion, Mexico City, Mexico; 25 Peninsula College of Medicine and Dentistry, Exeter, UK; 26 Division of Preventive Medicine, Brigham and Women’s Hospital, Boston, MA USA; 27 Division of Cardiovascular Medicine, Brigham and Women’s Hospital and Harvard Medical School, Boston, MA USA; 28 Department of Cardiology, University Hospital, Parma, Italy; 29 Department of Biology, Faculty of Natural Sciences, University of Haifa, Haifa, Israel; 30 Department of Medicine, Albert Einstein College of Medicine, Bronx, NY USA; 31 Department of Genetics, Albert Einstein College of Medicine, Bronx, NY USA; 32 Department of Quantitative Health Sciences, Lerner Research Institute, Cleveland Clinic, Cleveland, OH USA; 33 Gastroenterology Department, Sorbonne Université, APHP, Saint Antoine Hospital, Paris, France; 34 Framingham Heart Study, National Heart, Lung, & Blood Institute and Boston University, Framingham, MA USA; 35 Department of Medicine, Boston University School of Medicine, Boston, Massachusetts, USA; 36 Department of Epidemiology, Boston University School of Public Health, Boston, Massachusetts, USA; 37 Department of Biostatistics, Center for Statistical Genetics, University of Michigan, Ann Arbor, MI USA; 38 National Human Genome Research Institute, National Institutes of Health, Bethesda, MD USA; 39 The Charles Bronfman Institute for Personalized Medicine, Icahn School of Medicine at Mount Sinai, New York, NY USA; 40 Department of Biochemistry, Wake Forest School of Medicine, Winston-Salem, NC USA; 41 Center for Genomics and Personalized Medicine Research, Wake Forest School of Medicine, Winston-Salem, NC USA; 42 Center for Diabetes Research, Wake Forest School of Medicine, Winston-Salem, NC USA; 43 Department of Cardiovascular Sciences, University of Leicester, Leicester, UK; 44 Department of Cardiovascular Sciences and NIHR Leicester Biomedical Research Centre, University of Leicester, Leicester, UK; 45 Department of Epidemiology and Biostatistics, Imperial College London, London, UK; 46 Department of Cardiology, Ealing Hospital NHS Trust, Southall, UK; 47 Imperial College Healthcare NHS Trust, Imperial College London, London, UK; 48 Department of Medicine and Therapeutics, The Chinese University of Hong Kong, Hong Kong, China; 49 Program for Neuropsychiatric Research, McLean Hospital, Belmont, MA USA; 50 Department of Medicine, University of Mississippi Medical Center, Jackson, MI USA; 51 Department of Epidemiology, Colorado School of Public Health, Aurora, CO USA; 52 Department of Pharmacology, University of Illinois at Chicago, Chicago, IL USA; 53 Department of Medicine, University of Illinois at Chicago, Chicago, IL USA; 54 Department of Genetics, Texas Biomedical Research Institute, San Antonio, TX USA; 55 Department of Biostatistics, Boston University School of Public Health, Boston, MA USA; 56 Cardiac Arrhythmia Service, Cardiovascular Research Center, Massachusetts General Hospital, Boston, MA USA; 57 Cardiovascular Epidemiology and Genetics, Hospital del Mar Medical Research Institute (IMIM), Barcelona, Catalonia Spain; 58 Centro de Investigación en Red en Enfermedades Cardiovasculares (CIBERCV), Barcelona, Catalonia Spain; 59 Department of Medicine, Medical School, University of Vic-Central University of Catalonia, Vic, Catalonia Spain; 60 Institute for Cardiogenetics, University of Lübeck, Lübeck, Germany; 61 DZHK (German Research Centre for Cardiovascular Research), partner site Hamburg/Lübeck/Kiel, Lübeck, Germany; 62 University Heart Center, Lübeck, Lübeck, Germany; 63 Estonian Genome Center, Institute of Genomics, University of Tartu, Tartu, Estonia; 64 Clinic of Gastroenterology, Helsinki University Hospital, Helsinki University, Helsinki, Finland; 65 Diabetes Unit, Massachusetts General Hospital, Boston, MA USA; 66 Center for Genomic Medicine, Massachusetts General Hospital, Boston, MA USA; 67 Program in Metabolism, Broad Institute of MIT and Harvard, Cambridge, MA USA; 68 Institute of Clinical Molecular Biology (IKMB), Christian-Albrechts-University of Kiel, Kiel, Germany; 69 Bioinformatics Consortium, Massachusetts General Hospital, Boston, MA USA; 70 Department of Pathology, Massachusetts General Hospital, Boston, MA USA; 71 Cancer Center, Massachusetts General Hospital, Boston, MA USA; 72 Cancer Genome Computational Analysis Group, Broad Institute of MIT and Harvard, Cambridge, MA USA; 73 Endocrinology and Metabolism Department, Hadassah-Hebrew University Medical Center, Jerusalem, Israel; 74 Department of Psychiatry and Behavioral Sciences, SUNY Upstate Medical University, Syracuse, NY USA; 75 Institute for Genomic Medicine, Columbia University Medical Center, Hammer Health Sciences, New York, NY USA; 76 Department of Genetics and Development, Columbia University Medical Center, Hammer Health Sciences, New York, NY USA; 77 Centro de Investigacion en Salud Poblacional, Instituto Nacional de Salud Publica, Cuernavaca, Mexico; 78 Genomics, Diabetes and Endocrinology, Lund University, Lund, Sweden; 79 Institute for Molecular Medicine Finland (FIMM), HiLIFE, University of Helsinki, Helsinki, Finland; 80 Lund University Diabetes Centre, Malmö, Sweden; 81 Human Genetics Center, University of Texas Health Science Center at Houston, Houston, TX USA; 82 Department of Neurology, Columbia University, New York, NY USA; 83 Institute of Genomic Medicine, Columbia University, New York, NY USA; 84 Institute of Biomedicine, University of Eastern Finland, Kuopio, Finland; 85 Department of Psychiatry, Helsinki University Central Hospital, Lapinlahdentie, Helsinki, Finland; 86 Department of Medical Epidemiology and Biostatistics, Karolinska Institutet, Stockholm, Sweden; 87 Icahn School of Medicine at Mount Sinai, New York, NY USA; 88 Department of Neurology, Helsinki University Central Hospital, Helsinki, Finland; 89 Department of Public Health, Faculty of Medicine, University of Helsinki, Helsinki, Finland; 90 Cardiovascular Disease Initiative, Broad Institute of MIT and Harvard, Cambridge, MA USA; 91 Center for Genome Science, Korea National Institute of Health, Chungcheongbuk-do, South Korea; 92 MRC Centre for Neuropsychiatric Genetics & Genomics, Cardiff University School of Medicine, Cardiff, USA; 93 Department of Health, National Institute for Health and Welfare (THL), Helsinki, Finland; 94 Section of Cardiovascular Medicine, Department of Internal Medicine, Yale School of Medicine, Connecticut Center for Outcomes Research and Evaluation, Yale New Haven Hospital, New Haven, CT USA; 95 Division of Pediatric Gastroenterology, Emory University School of Medicine, Atlanta, GA USA; 96 Department of Internal Medicine, Seoul National University Hospital, Seoul, South Korea; 97 Institute of Clinical Medicine, The University of Eastern Finland, Kuopio, Finland; 98 Institute of Clinical Medicine Neurology, Kuopio University Hospital, Kuopio, Finland; 99 Department of Clinical Chemistry, Fimlab Laboratories and Finnish Cardiovascular Research Center-Tampere, Faculty of Medicine and Health Technology, Tampere University, Tampere, Finland; 100 The Mindich Child Health and Development Institute, Icahn School of Medicine at Mount Sinai, New York, NY USA; 101 Li Ka Shing Institute of Health Sciences, The Chinese University of Hong Kong, Hong Kong, China; 102 Hong Kong Institute of Diabetes and Obesity, The Chinese University of Hong Kong, Hong Kong, China; 103 Cardiovascular Research REGICOR Group, Hospital del Mar Medical Research Institute (IMIM), Barcelona, Catalonia Spain; 104 Department of Genetics, Harvard Medical School, Boston, MA USA; 105 Oxford Centre for Diabetes, Endocrinology and Metabolism, University of Oxford, Churchill Hospital, Headington, Oxford, UK; 106 Wellcome Centre for Human Genetics, University of Oxford, Oxford, UK; 107 Oxford NIHR Biomedical Research Centre, Oxford University Hospitals NHS Foundation Trust, John Radcliffe Hospital, Oxford, UK; 108 F Widjaja Foundation Inflammatory Bowel and Immunobiology Research Institute, Cedars-Sinai Medical Center, Los Angeles, CA USA; 109 Atherogenomics Laboratory, University of Ottawa Heart Institute, Ottawa, Canada; 110 Division of General Internal Medicine, Massachusetts General Hospital, Boston, MA USA; 111 Department of Clinical Sciences, University Hospital Malmo Clinical Research Center, Lund University, Malmo, Sweden; 112 Department of Clinical Sciences, Lund University, Skane University Hospital, Malmo, Sweden; 113 Instituto Nacional de Medicina Genómica (INMEGEN), Mexico City, Mexico; 114 Medical Research Institute, Ninewells Hospital and Medical School, University of Dundee, Dundee, UK; 115 Department of Molecular Medicine and Biopharmaceutical Sciences, Graduate School of Convergence Science and Technology, Seoul National University, Seoul, South Korea; 116 Department of Psychiatry, Keck School of Medicine at the University of Southern California, Los Angeles, CA USA; 117 Department of Psychiatry and Behavioral Sciences, Johns Hopkins University School of Medicine, Baltimore, MA USA; 118 Division of Genetics and Epidemiology, Institute of Cancer Research, London, USA; 119 Research Unit of Clinical Neuroscience, University of Oulu, Oulu, Finland; 120 Research Center, Montreal Heart Institute, Montreal, Quebec Canada; 121 Department of Medicine, Faculty of Medicine, Université de Montréal, Montreal, Quebec Canada; 122 Department of Biomedical Informatics, Vanderbilt University Medical Center, Nashville, TN USA; 123 Department of Medicine, Vanderbilt University Medical Center, Nashville, TN USA; 124 Department of Biostatistics and Epidemiology, Perelman School of Medicine at the University of Pennsylvania, Philadelphia, PA USA; 125 Department of Medicine, Perelman School of Medicine at the University of Pennsylvania, Philadelphia, PA USA; 126 Center for Non-Communicable Diseases, Karachi, Pakistan; 127 National Institute for Health and Welfare, Helsinki, Finland; 128 Deutsches Herzzentrum München, Munich, Germany; 129 Technische Universität München, Munich, Germany; 130 Division of Cardiovascular Medicine, Nashville VA Medical Center and Vanderbilt University, School of Medicine, Nashville, TN USA; 131 Department of Psychiatry, Icahn School of Medicine at Mount Sinai, New York, NY USA; 132 Department of Genetics and Genomic Sciences, Icahn School of Medicine at Mount Sinai, New York, NY USA; 133 Institute for Genomics and Multiscale Biology, Icahn School of Medicine at Mount Sinai, New York, NY USA; 134 Institute of Clinical Medicine Neurology, University of Eastern Finland, Kuopio, Finland; 135 Department of Twin Research and Genetic Epidemiology, King’s College London, London, UK; 136 Departments of Genetics and Psychiatry, University of North Carolina, Chapel Hill, NC USA; 137 Saw Swee Hock School of Public Health, National University of Singapore, National University Health System, Singapore, Singapore; 138 Department of Medicine, Yong Loo Lin School of Medicine, National University of Singapore, Singapore, Singapore; 139 Duke-NUS Graduate Medical School, Singapore, Singapore; 140 Life Sciences Institute, National University of Singapore, Singapore, Singapore; 141 Department of Statistics and Applied Probability, National University of, Singapore, Singapore; 142 Folkhälsan Institute of Genetics, Folkhälsan Research Center, Helsinki, Finland; 143 HUCH Abdominal Center, Helsinki University Hospital, Helsinki, Finland; 144 Department of Psychiatry, Center for Behavioral Genomics, University of California, San Diego, CA USA; 145 Institute of Genomic Medicine, University of California, San Diego, CA USA; 146 Juliet Keidan Institute of Pediatric Gastroenterology, Shaare Zedek Medical Center, The Hebrew University of Jerusalem, Jerusalem, Israel; 147 Instituto de Investigaciones Biomédicas UNAM, Mexico City, Mexico; 148 Instituto Nacional de Ciencias Médicas y Nutrición Salvador Zubirán, Mexico City, Mexico; 149 Radcliffe Department of Medicine, University of Oxford, Oxford, UK; 150 Department of Gastroenterology and Hepatology, University of Groningen and University Medical Center Groningen, Groningen, The Netherlands; 151 Department of Physiology and Biophysics, University of Mississippi Medical Center, Jackson, MS USA; 152 Program in Infectious Disease and Microbiome, Broad Institute of MIT and Harvard, Cambridge, MA USA; 153 Center for Computational and Integrative Biology, Massachusetts General Hospital, Boston, MA USA; 154 Department of Psychiatry & Human Behavior, University of California Irvine, Irvine, CA USA

**Keywords:** Genome informatics, Chromosome abnormality, Structural variation, Genomics, Mutation

## Abstract

Structural variants (SVs) rearrange large segments of DNA^1^ and can have profound consequences in evolution and human disease^2,3^. As national biobanks, disease-association studies, and clinical genetic testing have grown increasingly reliant on genome sequencing, population references such as the Genome Aggregation Database (gnomAD)^4^ have become integral in the interpretation of single-nucleotide variants (SNVs)^5^. However, there are no reference maps of SVs from high-coverage genome sequencing comparable to those for SNVs. Here we present a reference of sequence-resolved SVs constructed from 14,891 genomes across diverse global populations (54% non-European) in gnomAD. We discovered a rich and complex landscape of 433,371 SVs, from which we estimate that SVs are responsible for 25–29% of all rare protein-truncating events per genome. We found strong correlations between natural selection against damaging SNVs and rare SVs that disrupt or duplicate protein-coding sequence, which suggests that genes that are highly intolerant to loss-of-function are also sensitive to increased dosage^6^. We also uncovered modest selection against noncoding SVs in *cis*-regulatory elements, although selection against protein-truncating SVs was stronger than all noncoding effects. Finally, we identified very large (over one megabase), rare SVs in 3.9% of samples, and estimate that 0.13% of individuals may carry an SV that meets the existing criteria for clinically important incidental findings^7^. This SV resource is freely distributed via the gnomAD browser^8^ and will have broad utility in population genetics, disease-association studies, and diagnostic screening.

## Abstract (web summary)

A large empirical assessment of sequence-resolved structural variants from 14,891 genomes across diverse global populations in the Genome Aggregation Database (gnomAD) provides a reference map for disease-association studies, population genetics, and diagnostic screening.

## Main

**Fig. 1:**
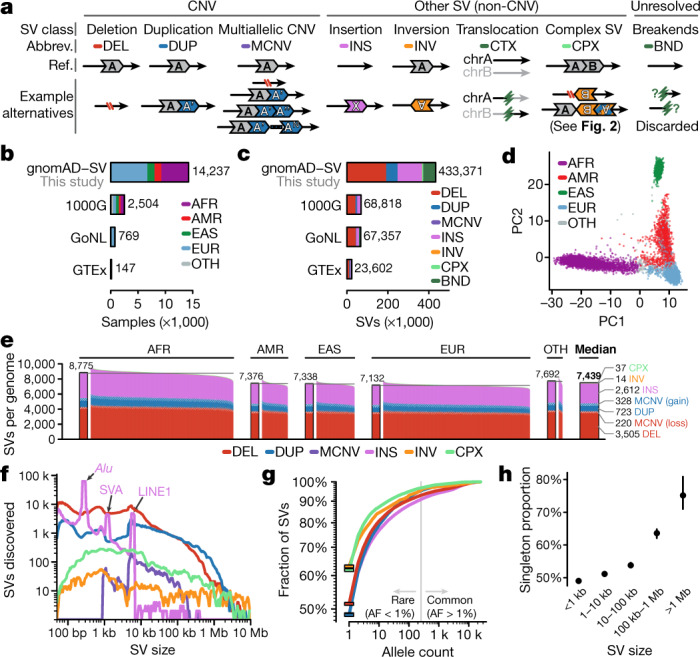
Properties of SVs across human populations. **a**, SV classes catalogued in this study. We also documented unresolved non-reference ‘breakends’ (BNDs), but they were excluded from all analyses as low-quality variants. **b**, After quality control, we analysed 14,237 samples across continental populations, including African/African American (AFR), Latino (AMR), East Asian (EAS), and European (EUR), or other populations (OTH). Three publicly available WGS-based SV datasets are provided for comparison (1000 Genomes Project (1000G), approximately 7× coverage; Genome of the Netherlands Project (GoNL), around 13× coverage; Genotype-Tissue Expression Project (GTEx), approximately 50× coverage)^1,16,17^. **c**, We discovered 433,371 SVs, and provide counts from previous studies for comparison^1,16,17^. **d**, A principal component (PC) analysis of genotypes for 15,395 common SVs separated samples along axes corresponding to genetic ancestry. **e**, The median genome contained 7,439 SVs. **f**, Most SVs were small. Expected *Alu*, SVA and LINE1 mobile element insertion peaks are marked at approximately 300 bp, 2.1 kb and 6 kb, respectively. **g**, Most SVs were rare (allele frequency (AF) < 1%), and 49.8% of SVs were singletons (solid bars). **h**, Allele frequencies were inversely correlated with SV size across all 335,470 resolved SVs in unrelated individuals. Values are mean and 95% confidence interval from 100-fold bootstrapping. Colour codes are consistent between **a**, **c**, **e**–**h**, and between **b** and **d**.

SVs are DNA rearrangements that involve at least 50 nucleotides^1^. By virtue of their size and abundance, SVs represent an important mutational force that shape genome evolution and function^2,3^, and contribute to germline and somatic diseases^9–11^. The profound effect of SVs is also attributable to the numerous mechanisms by which they can disrupt protein-coding genes and *cis*-regulatory architecture^12^. SVs can be grouped into mutational classes that include ‘unbalanced’ gains or losses of DNA (for example, copy-number variants, CNVs), and ‘balanced’ rearrangements that occur without corresponding dosage alterations (such as inversions and translocations)^1^ (Fig. 1a). Other common forms of SVs include mobile elements that insert themselves throughout the genome, and multiallelic CNVs (MCNVs) that can exist at high copy numbers^1^. More recently, exotic species of complex SVs have been discovered that involve two or more distinct SV signatures in a single mutational event interleaved on the same allele, and can range from CNV-flanked inversions to rare instances of localized chromosome shattering, such as chromothripsis^13,14^. The diversity of SVs in humans is therefore far greater than has been widely appreciated, as is their influence on genome structure and function.

Although SVs alter more nucleotides per genome than SNVs and short insertion/deletion variants (indels; <50 bp)^1^, surprisingly little is known about their mutational spectra on a global scale. The largest published population study of SVs using whole-genome sequencing (WGS) remains the 1000 Genomes Project (*n* = 2,504; 7× sequence coverage)^1^, and the substantial technical challenges of SV discovery from WGS^15^ has led to non-uniform SV analyses across contemporary studies^16–20^. Moreover, short-read WGS is unable to capture a subset of SVs accessible to more expensive niche technologies, such as long-read WGS^21^. Owing to the combination of these challenges, SV references are dwarfed by contemporary resources for short variants, such as the Exome Aggregation Consortium (ExAC) and its successor, the Genome Aggregation Database (gnomAD), which have jointly analysed more than 140,000 individuals^4,6^. Publicly available resources such as ExAC and gnomAD have transformed many aspects of human genetics, including defining sets of genes constrained against damaging coding mutations^6^ and providing frequency filters for variant interpretation^5^. As short-read WGS is rapidly becoming the predominant technology in large-scale human disease studies, and will probably displace conventional methods for diagnostic screening, there is a mounting need for comparable references of SVs across global populations.

In this study, we developed gnomAD-SV, a sequence-resolved reference for SVs from 14,891 genomes. Our analyses revealed diverse mutational patterns among SVs, and principles of selection acting against reciprocal dosage changes in genes and noncoding *cis*-regulatory elements. From these analyses, we determined that SVs represent more than 25% of all rare protein-truncating events per genome, emphasizing the unrealized potential of routine SV detection in WGS studies. This SV reference has been integrated into the gnomAD browser (http://gnomad.broadinstitute.org) with no restrictions on reuse so that it can be mined for new insights into genome biology and applied as a resource to interpret SVs in diagnostic screening.

## SV discovery and genotyping

We analysed WGS data for 14,891 samples (average coverage of 32×) aggregated from large-scale sequencing projects, of which 14,237 (95.6%) passed all quality thresholds, representing a general adult population depleted for severe Mendelian diseases (median age of 49 years) (Supplementary Table 1, Supplementary Figs. 1, 2). This cohort included 46.1% European, 34.9% African or African American, 9.2% East Asian, and 8.7% Latino samples, as well as 1.2% samples from admixed or other populations (Fig. 1). Following family-based analyses using 970 parent–child trios for quality assessments, we pruned all first-degree relatives from the cohort, retaining 12,653 unrelated genomes for subsequent analyses.

**Fig. 2:**
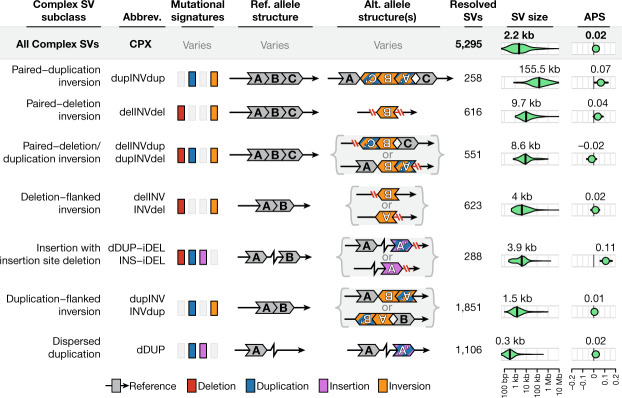
Complex SVs are abundant in the human genome. We resolved 5,295 complex SVs across 11 mutational subclasses, 73.7% of which involved at least one inversion. Each subclass is detailed here, including their mutational signatures, structures, abundance, density of SV sizes (vertical line indicates median size), and allele frequencies. Five pairs of subclasses have been collapsed into single rows due to mirrored or similar alternative allele structures (for example, delINV versus INVdel). Two complex SVs did not conform to any subclass (Extended Data Fig. 8).

**Extended Data Fig. 1:**
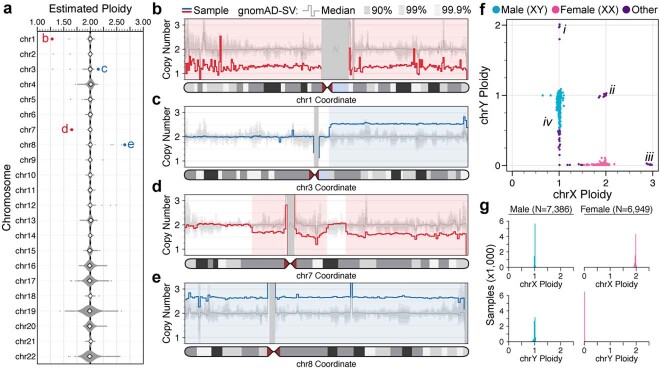
Detection of chromosome-scale dosage alterations. We estimated ploidy (that is, whole-chromosome copy number) for all 24 chromosomes per sample. **a**, Distribution of autosome ploidy estimates across 14,378 samples passing initial data quality thresholds. White diamonds indicate medians. Individual points are outlier samples at least three standard deviations away from the cohort-wide mean. The outlier points marked in red and blue correspond to the samples highlighted in **b**–**e**. **b**–**e**, Samples with outlier autosome ploidy estimates typically contained somatic or mosaic chromosomal abnormalities, such as somatic aneuploidy of chromosome 1 (chr1) (**b**) or chromosome 8 (**e**), or large focal somatic or mosaic CNVs on chromosome 3 (**c**) and chromosome 7 (**d**). Each panel depicts copy-number estimates in 1-Mb bins for each rearranged sample in red or blue. Dark, medium and light-grey background shading indicates the range of copy number estimates for 90%, 99% and 99.9% of all gnomAD-SV samples, respectively, and the medium grey line indicates the median copy number estimate across all samples. Regions of unalignable N-masked bases >1 Mb in the reference genome are masked with grey rectangles. **f**, Sex chromosome ploidy estimates for all samples from **a**. We inferred karyotypic sex by clustering samples to their nearest integer ploidy for sex chromosomes. Several abnormal sex chromosome ploidies are marked, including XYY (i), XXY (ii), XXX (iii), and mosaic loss-of-Y (iv). **g**, Histogram representation of the data from **f**. Essentially all samples conformed to canonical sex chromosome ploidies.

**Extended Data Fig. 2:**
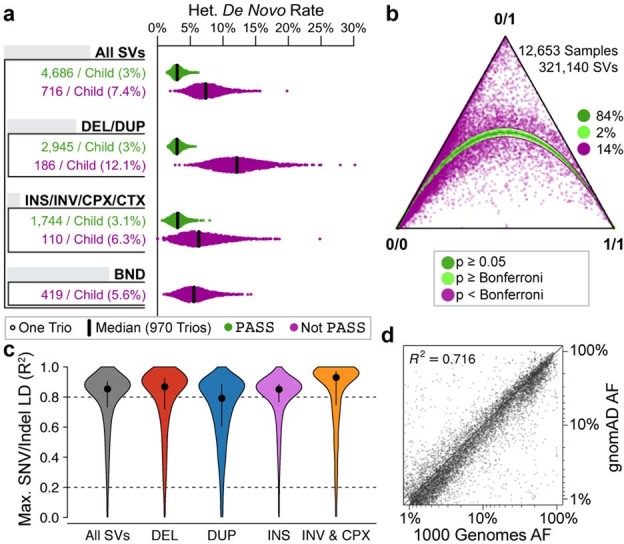
Benchmarking the technical qualities of the gnomAD-SV callset. We evaluated the quality of gnomAD-SV with seven orthogonal analyses detailed in Supplementary Table 4, Supplementary Figs. 6–9 and Supplementary Note 1. Four core analyses are presented here. **a**, Apparent rates of de novo (that is, spontaneous) heterozygous SVs per child across 970 parent–child trios. Each point is a single trio, and vertical lines denote medians. Given the expected mutation rate of SVs accessible to short-read WGS^1,20^ (<1 true de novo SV per trio; see also Fig. 3a), effectively all de novo SVs represented a combination of false-positive genotypes in children and/or false-negative genotypes in parents. SVs passing all filters and included in the final gnomAD-SV callset (‘pass’) are shown in green. For comparison, variants that did not pass post hoc site-level filters (‘not pass’) are also shown in purple. **b**, Hardy–Weinberg equilibrium (HWE) metrics for all biallelic SVs localized to autosomes. Deviation from HWE was assessed using a chi-square goodness-of-fit test with one degree of freedom. Vertex labels reflect genotypes: 0/0 denotes homozygous reference; 0/1 denotes heterozygous; and 1/1 denotes homozygous alternate, with all sites shaded by chi-squared *P* value. **c**, Linkage disequilibrium between SVs and SNVs or indels for 23,706 common (allele frequency > 1%) SVs represented as cross-population maximum *R*^2^ values after excluding repetitive and low-complexity regions (see Supplementary Fig. 7). Points and vertical bars represent medians and interquartile ranges, respectively. **d**, Correlation of allele frequency (AF) for 37,907 common SVs captured by both the 1000 Genomes Project and gnomAD-SV^1^. Pearson’s correlation coefficient (*R*^2^) is provided.

We discovered and genotyped SVs using a cloud-based, multi-algorithm pipeline for short-read WGS (Supplementary Fig. 3), which we prototyped in a study of 519 autism quartet families^20^. This pipeline integrated four orthogonal evidence types to capture SVs across the size and allele frequency spectra, including six classes of canonical SVs (Fig. 1a) and 11 subclasses of complex SVs^22^ (Fig. 2). We augmented this pipeline with new methods to account for the technical heterogeneity of aggregated datasets (Extended Data Fig. 1, Supplementary Figs. 4, 5), and discovered 433,371 SVs (Fig. 1c). After excluding low-quality SVs, which were predominantly (61.6%) composed of incompletely resolved breakpoint junctions (that is, ‘breakends’) that lack interpretable alternative allele structures for functional annotation and produce high false-discovery rates^20^ (Extended Data Fig. 2a), we retained 335,470 high-quality SVs for subsequent analyses (Supplementary Table 3). This final set of high-quality SVs corresponded to a median of 7,439 SVs per genome, or more than twice the number of variants per genome captured by previous WGS-based SV studies such as the 1000 Genomes Project (3,441 SVs per genome from approximately 7× coverage WGS), which underscores the benefits of high-coverage WGS and improved multi-algorithm ensemble methods for SV discovery.

**Extended Data Fig. 3:**
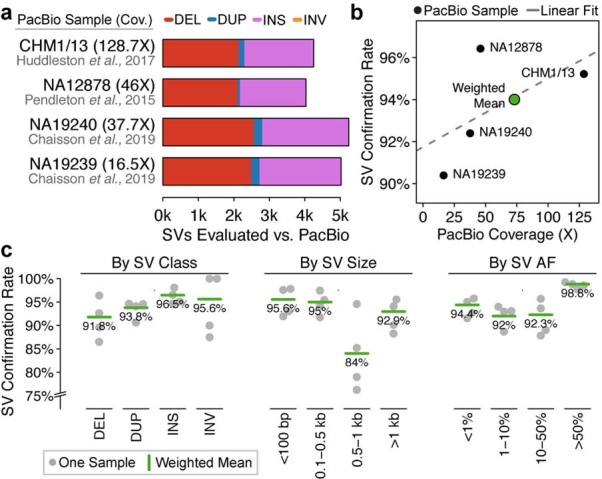
In silico confirmation of SVs in gnomAD-SV with long-read WGS. We used Pacific Biosciences (PacBio) long-read WGS data available for four samples in this study to perform in silico confirmation to estimate the positive predictive value and breakpoint accuracy for SVs in gnomAD-SV^21,45,46^ (Supplementary Fig. 10). **a**, Counts of SVs evaluated per sample in this analysis. SVs were restricted to those with breakpoint-level read support (that is, ‘split-read’ evidence, 92.8% of all SVs) and did not have breakpoints localized to annotated simple repeats or segmental duplications. **b**, An iterative local long-read WGS realignment algorithm, VaPoR^47^, was used to perform in silico confirmation of SVs predicted from short-read WGS in gnomAD-SV. As noted by the VaPoR developers^47^, the performance of this approach was sensitive to the sequencing depth of long-read WGS data. Therefore, the weighted mean of the four samples was used as a study-wide long-read WGS confirmation rate, weighting the confirmation rate of each sample based on the square root of its long-read WGS sequencing depth. **c**, Confirmation rates stratified by SV class, size and allele frequency. A mean of 4,829 SVs per sample were assessed. Horizontal green bars denote weighted means.

Given that there are no gold-standard benchmarking procedures for SVs from WGS, we evaluated the technical qualities of gnomAD-SV using seven orthogonal approaches. These analyses are described in detail in Extended Data Figs. 2, 3, Supplementary Figs. 6–12, Supplementary Table 4 and Supplementary Note 1, but we highlight just a few here to demonstrate that gnomAD-SV conforms to many fundamental principles of population genetics, including Mendelian segregation, genotype distributions, and linkage disequilibrium. We found that the precision of gnomAD-SV was comparable to our previous study of 519 autism quartets that attained a 97% molecular validation rate for all de novo SV predictions^20^: in gnomAD, analyses of 970 parent–child trios indicated a median Mendelian violation rate of 3.8% and a heterozygous de novo rate of 3.0%. We also observed that 86% of SVs were in Hardy–Weinberg equilibrium, and common SVs were in strong linkage disequilibrium with nearby SNVs or indels (median peak *R*^2^ = 0.85). We performed extensive in silico confirmation of 19,316 SVs predicted from short-read WGS using matched long-read WGS from four samples^21,23^, finding a 94.0% confirmation rate with breakpoint-level read evidence, and revealing that 59.8% of breakpoint coordinates were accurate within a single nucleotide of the long-read data. These and other benchmarking approaches suggested that gnomAD-SV was sufficiently sensitive and specific to be used as a reference dataset for most applications in human genomics.

## Population genetics and genome biology

**Extended Data Fig. 4:**
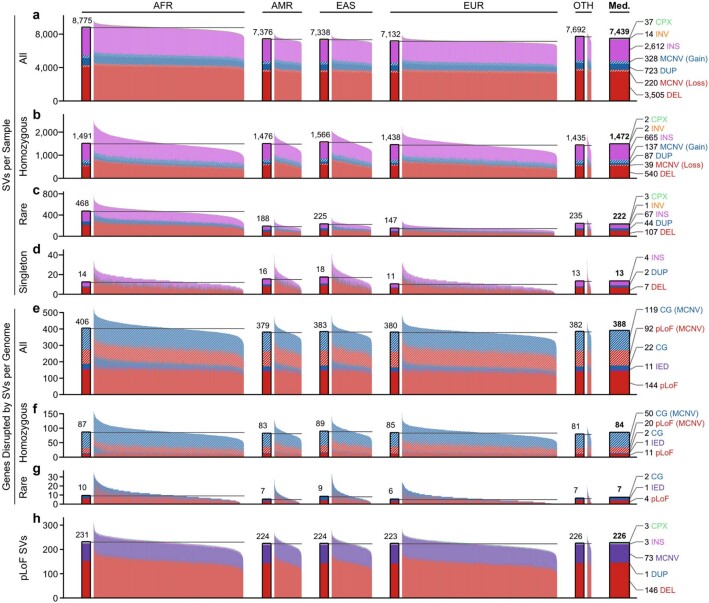
SVs contribute a substantial burden of rare, homozygous, and coding mutations per genome. **a**–**d**, Counts of SVs per genome across a variety of parameters, corresponding to median counts of total SVs (**a**), homozygous SVs (**b**), rare SVs (**c**) and singleton SVs (**d**). Samples are grouped by population and coloured by SV types. The solid bar to the left of each population indicates the population median. **e**–**g**, Median counts of genes disrupted by SVs per genome when considering all SVs (including MCNVs) (**e**), homozygous SVs (including MCNVs) (**f**), and rare SVs (**g**). Colours correspond to predicted functional consequence. **h**, Counts of pLoF SVs per genome. For certain categories, such as genes disrupted by rare SVs per genome, a subset of samples (<5%) were enriched above the population average, as expected for individuals carrying large, rare CNVs predicted to cause the disruption of dozens or hundreds of genes (see Extended Data Fig. 1); for the purposes of visualization, the *y* axis for all panels has been restricted to a maximum of three interquartile ranges above the third quartile across all samples for each category.

**Extended Data Fig. 5:**
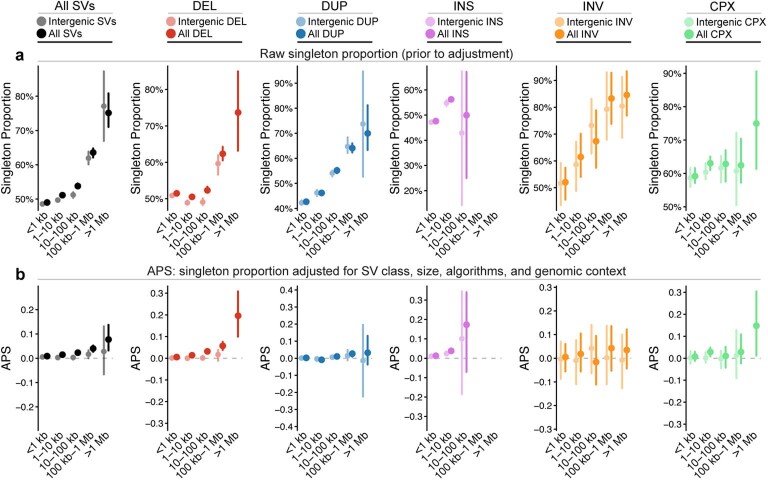
Rearrangement size is a primary determinant of allele frequency for most classes of SVs. **a**, Proportion of singleton SVs in five SV size bins for each class of biallelic SVs considered in this study. Intergenic SVs (light colours; *n* = 206,954) exhibited reduced singleton proportions when compared to all SVs (dark colours; *n* = 335,470) of the same size and class. Bars reflect 95% confidence intervals from 100-fold bootstrapping. Categories with fewer than ten SVs are not shown. **b**, To account for the strong dependency of singleton proportion on SV size and class, we developed the APS metric, which normalizes singleton proportions using SV-specific technical and genomic covariates to permit comparisons of the frequency spectra across SV classes (see Supplementary Fig. 14). The same data as in **a** are shown, transformed onto the APS scale, which shows effectively no dependency on SV size for intergenic SVs. Bars reflect 95% confidence intervals from 100-fold bootstrapping. Residual deviation from APS = 0 is maintained when considering all SVs, owing to APS being intentionally calibrated to intergenic SVs as a proxy for neutral variation. Because larger SVs are more likely to be gene-disruptive, they upwardly bias the APS point estimates due to residual negative selection not captured by SV size alone. Counts of SVs per category for both **a** and **b** are listed in Supplementary Table 9.

The distribution of SVs across samples matched expectations based on human demographic history, with the top three components of genetic variance separating continental populations (Fig. 1d, Supplementary Fig. 13). African and African American samples exhibited the greatest genetic diversity and their common SVs were in weaker linkage disequilibrium with nearby short variants than Europeans, whereas East Asians featured the highest levels of homozygosity (Fig. 1e, Extended Data Fig. 4a–d, Supplementary Fig. 7). The mutational diversity of gnomAD-SV was extensive: we completely resolved 5,295 complex SVs across 11 mutational subclasses, of which 3,901 (73.7%) involved inverted segments (Fig. 2), confirming that inversion variation is predominantly composed of complex SVs rather than canonical inversions^1,24^. Across all SV classes, most SVs were small (median size of 331 bp) and rare (allele frequency < 1%; 92% of SVs), with half of all SVs (49.8%) appearing as ‘singletons’ (that is, only one allele observed across all samples) (Fig. 1f, g). Although the proportion of singletons varied by SV class, it was strongly dependent on SV size across all classes, which suggests that the amount of DNA rearranged is a key determinant of selection against most SVs (Fig. 1h, Extended Data Fig. 5a).

**Fig. 3:**
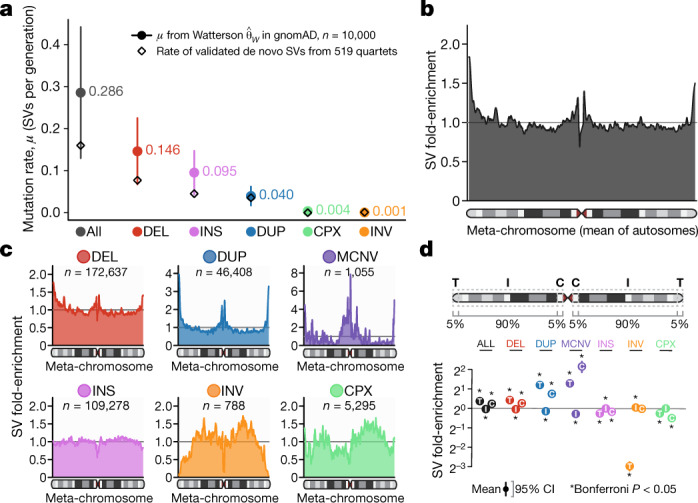
Genome-wide mutational patterns of SVs. **a**, Mutation rates (*μ*) from the Watterson estimator for each SV class^26^. Bars represent 95% confidence intervals. Rates of molecularly validated de novo SVs from 519 quartet families are provided for comparison^20^. **b**, Smoothed enrichment of SVs per 100-kb window across the average of all autosomes normalized by chromosome arm length (a ‘meta-chromosome’) (Supplementary Fig. 16). **c**, The distribution of SVs along the meta-chromosome was dependent on variant class. **d**, SV enrichment by class and chromosomal position provided as mean and 95% confidence intervals (CI). C, centromeric; I, interstitial; T, telomeric. *P* values were computed using a two-sided *t*-test and were Bonferroni-adjusted for 21 comparisons. *P ≤ 2.38 × 10^−3^.

Mutation rate estimates for SVs have remained elusive owing to limited sample sizes, poor resolution of conventional technologies, technical challenges of SV discovery, and use of cell line-derived DNA in population studies^1,25^. Here, we used the Watterson estimator^26^ to project a mean mutation rate of 0.29 de novo SVs (95% confidence interval 0.13–0.44) per generation in regions of the genome accessible to short-read WGS, or roughly one new SV every 2–8 live births, with mutation rates varying markedly by SV class (Fig. 3a). Although this imperfect method extrapolates from data pooled across unrelated individuals, we previously demonstrated comparable rates from molecularly validated observations in 519 quartet families^20^. Like mutation rates, the distribution of SVs throughout the genome was non-uniform, significantly correlated with repetitive sequence contexts, and was enriched near centromeres and telomeres^23^ (Supplementary Fig. 16). These trends were dependent on SV class, as biallelic deletions and duplications were predominantly enriched at telomeres, whereas MCNVs were enriched in centromeric segmental duplications (Fig. 3b–d). Given the reduced sensitivity of short-read WGS in repetitive sequences, this study certainly underestimates the true SV mutation rates; nevertheless, these analyses implicate several aspects of chromosomal context and SV class in determining SV mutation rates throughout the genome.

## Dosage sensitivity of coding and noncoding loci

**Fig. 4:**
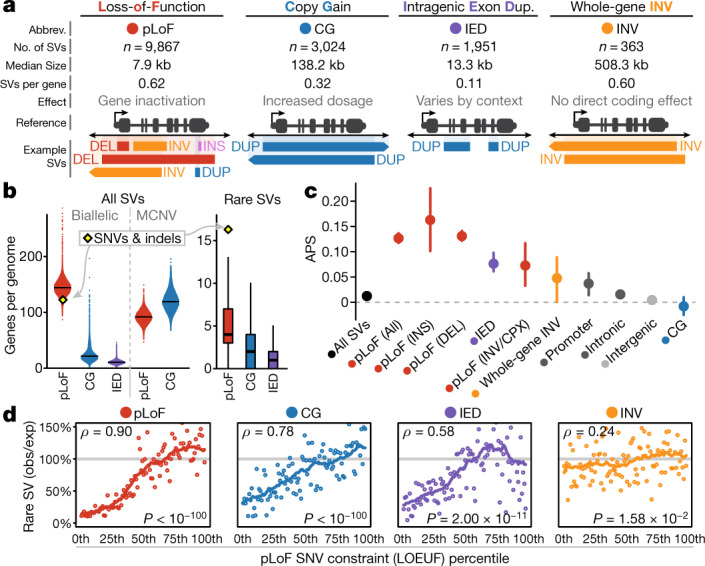
Pervasive selection against SVs in genes mirrors coding short variants. **a**, Four categories of gene-overlapping SVs, with counts of total SVs, median SV size, and mean SVs per gene in gnomAD-SV. **b**, Count of genes altered by SVs per genome. Horizontal lines indicate medians. Sample sizes per category listed in Supplementary Table 9. **c**, APS value for SVs overlapping genes. Bars indicate 100-fold bootstrapped 95% confidence intervals. SVs per category listed in Supplementary Table 9. **d**, Relationships of constraint against pLoF SNVs versus gene-overlapping SVs in 100 bins of around 175 genes each, ranked by SNV constraint^4^. Correlations were assessed with a two-sided Spearman correlation test. Solid lines represent 21-point rolling means. See Supplementary Fig. 19 for comparisons to missense constraint.

Owing to their size and mutational diversity, SVs can have varied consequences on protein-coding genes^12^ (Fig. 4a, Supplementary Fig. 17). In principle, any SV can result in predicted loss-of-function (pLoF), either by deleting coding nucleotides or altering open-reading frames. Coding duplications can result in copy-gain of entire genes, or of a subset of exons within a gene (referred to here as intragenic exonic duplication, or IED). The average genome in gnomAD-SV contained a mean of 179.8 genes altered by biallelic SVs (144.3 pLoF, 24.3 copy-gain, and 11.2 IED), of which 11.6 were predicted to be completely inactivated by homozygous pLoF (Fig. 4b, Extended Data Fig. 4e–h). When restricted to rare (allele frequency < 1%) SVs, we observed a mean of 10.2 altered genes per genome (5.5 pLoF, 3.4 copy-gain, and 1.3 IED). By comparison, a companion gnomAD paper estimated 122.4 pLoF short variants per genome, of which 16.3 were rare^4^. These analyses suggest that 29.4% of rare heterozygous gene inactivation events per individual are contributed by SVs, or conservatively 25.2% of pLoF events if we exclude IEDs given the context-dependence of their functional impact.

**Extended Data Fig. 6:**
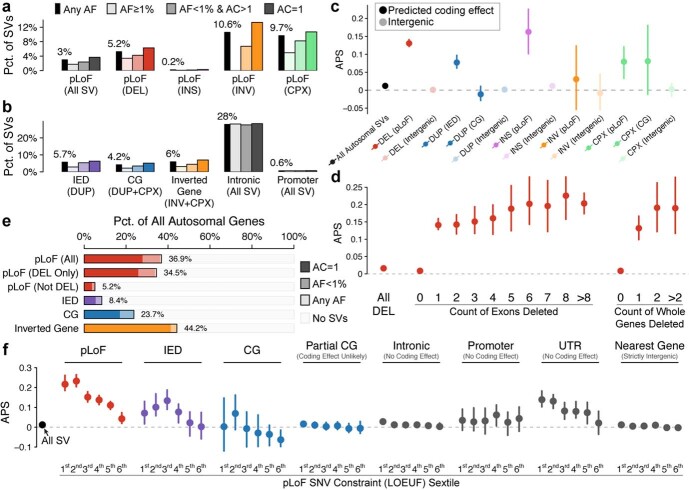
Most SVs within genes appear under negative selection. **a**, Enrichments for pLoF consequences among rare and singleton SVs across SV classes. **b**, Enrichments for non-pLoF functional consequences among rare and singleton SVs across SV classes. **c**, Adjusted proportion of singletons across SV types and functional consequences. **d**, APS among deletions relative to count of exons and whole genes deleted. **e**, Fractions of all autosomal protein-coding genes with at least one SV across a variety of functional consequences. **f**, Relationship of APS and constraint against pLoF SNVs^4^. For this analysis, intronic, promoter and UTR SVs were required to have precise breakpoints (that is, have ‘split-read’ support) to protect against any cryptic overlap with coding sequence unable to be annotated due to imprecise breakpoints. For **c**, **d** and **f**, points and vertical bars represent 95% confidence intervals from 100-fold bootstrapping, respectively. Counts of SVs per category in **c** and **d** are provided in Supplementary Table 9. For **d** and **f**, deletions in highly repetitive or low-complexity sequence (≥30% coverage by annotated segmental duplications or simple repeats) were excluded.

A fundamental question in human genetics is the degree to which natural selection acts on coding and noncoding loci. The proportion of singleton variants has been established as a proxy for strength of selection^6^; however, this metric is confounded for SVs given the strong correlation between allele frequency and SV size, among other factors. Therefore, we developed a new metric, adjusted proportion of singletons (APS), to account for SV class, size, genomic context, and other technical covariates (Extended Data Fig. 5, Supplementary Fig. 14). Under this normalized APS metric, a value of zero corresponds to a singleton proportion comparable to intergenic SVs, whereas values greater than zero reflect purifying selection, similar to the ‘mutability-adjusted proportion of singletons’ (MAPS) metric used for SNVs^6^. Applying this APS model revealed signals of pervasive selection against nearly all classes of SVs that overlap genes, including intronic SVs, whole-gene inversions, SVs in gene promoters, and deletions as small as a single exon (Fig. 4c, Extended Data Fig. 6, Supplementary Fig. 18). The one notable exception was copy-gain duplications, which showed no clear evidence of selection beyond what could already be explained by their sizes, which were vastly larger than non-copy-gain duplications (median copy-gain duplication size = 134.8 kb; median non-copy-gain duplication size = 2.7 kb; one-tailed Wilcoxon test, *W* = 1.18 × 10^8^, *P* < 10^−100^). This result could have numerous explanations, but it is consistent with the known diverse evolutionary roles of gene duplication events, including positive selection reported in humans^27,28^.

Methods that quantify evolutionary constraint on a per-gene basis, such as the probability of intolerance to heterozygous pLoF variation (pLI)^6^ and the pLoF observed/expected upper fraction (LOEUF)^4^, have become core resources in human genetics. Nearly all existing metrics, including pLI and LOEUF, are derived from SNVs. Although previous studies have attempted to compute similar scores using large CNVs detected by microarray and exome sequencing^29,30^, or to correlate deletions with pLI^18^, no gene-level metrics comparable to LOEUF exist for SVs at WGS resolution. To gain insight into this problem, we built a model to estimate the depletion of rare SVs per gene compared to expectations based on gene length, genomic context, and the structure of exons and introns. This model is imperfect, as current sample sizes are too sparse to derive precise gene-level metrics of constraint from SVs. Nevertheless, we found strong concordance between the depletion of rare pLoF SVs and existing pLoF and missense SNV constraint metrics^4^ (pLoF Spearman correlation test, *ρ* = 0.90, *P* < 10^−100^) (Fig. 4d, Supplementary Fig. 19). Notably, a comparable positive correlation was also observed for copy-gain SVs and SNV constraint (pLoF Spearman correlation test, *ρ* = 0.78, *P* < 10^−100^), whereas a weaker yet significant correlation was detected for IEDs (pLoF Spearman correlation test, *ρ* = 0.58, *P* = 2.0 × 10^−11^). As orthogonal support for these trends, we identified an inverse correlation between APS and SNV constraint across all functional categories of SVs, which was consistent with our observed depletion of rare, functional SVs in constrained genes (Extended Data Fig. 6f). These comparisons confirm that selection against most classes of gene-altering SVs mirrors patterns observed for short variants^18,30^. They further suggest that SNV-derived constraint metrics such as LOEUF capture a general correspondence between haploinsufficiency and triplosensitivity for a large fraction of genes in the genome. It therefore appears that the most highly pLoF-constrained genes not only are sensitive to pLoF, but also are more likely to be intolerant to increased dosage and other functional alterations.

**Fig. 5:**
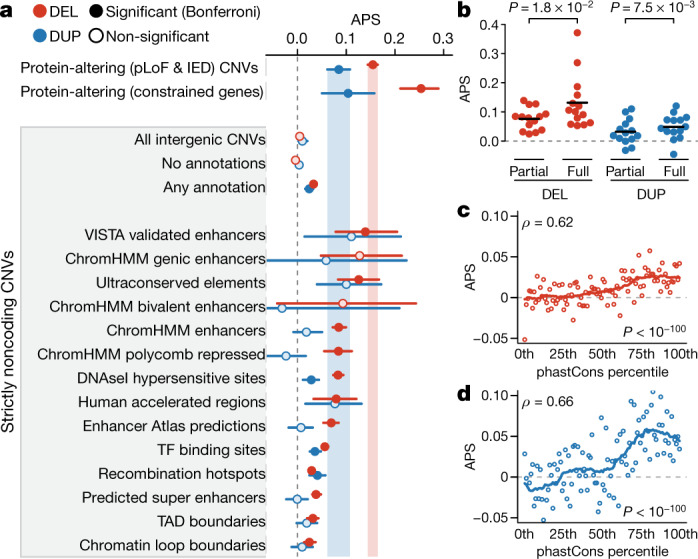
Dosage sensitivity in the noncoding genome. **a**, Strength of selection (APS) for noncoding CNVs overlapping 14 categories of noncoding elements (Supplementary Table 5). Bars reflect 95% confidence intervals from 100-fold bootstrapping. Each category was compared to neutral variation (APS = 0) using a one-tailed *t*-test. Categories surpassing Bonferroni-corrected significance for 32 comparisons are indicated with dark shaded points. SVs per category listed in Supplementary Table 9. DEL, deletion; DUP, duplication; TAD, topologically associating domain; TF, transcription factor. **b**, CNVs that completely covered elements (‘full’) had significantly higher average APS values than CNVs that only partially covered elements (‘partial’). *P* values calculated using a two-tailed paired two-sample *t*-test for the 14 categories from **a**. **c**, **d**, Spearman correlations between sequence conservation and APS for noncoding deletions (*n* = 143,353) (**c**) and duplications (*n* = 30,052) (**d**). Noncoding CNVs were sorted into 100-percentile bins based on the sum of the phastCons scores overlapped by the CNV. Correlations were assessed with a two-sided Spearman correlation test. Solid lines represent 21-point rolling means.

In contrast to the well-studied effects of coding variation, the effects of noncoding SVs on regulatory elements are largely unknown. There are a handful of examples of SVs with strong noncoding effects, although they are scarce in humans and model organisms^31,32^. In gnomAD-SV, we explored noncoding dosage sensitivity across 14 regulatory element classes, ranging from high-confidence experimentally validated enhancers to large databases of computationally predicted elements (Supplementary Table 5). We found that noncoding CNVs overlapping most element classes had increased proportions of singletons, although none exceeded the APS observed for pLoF SVs (Fig. 5a). In general, the effects of noncoding deletions appeared stronger than noncoding duplications, and CNVs predicted to delete or duplicate entire elements were under stronger selection than partial element disruption (Fig. 5b). We also observed that primary sequence conservation was correlated with selection against noncoding CNVs (Fig. 5c, d), which provides a foothold for future work on interpretation and functional effect prediction for noncoding SVs. Broadly, these results followed trends we observed for protein-coding SVs, which we interpreted as evidence for weak but widespread selection against CNVs altering most classes of annotated regulatory elements.

## Trait association and clinical genetics

**Extended Data Fig. 7:**
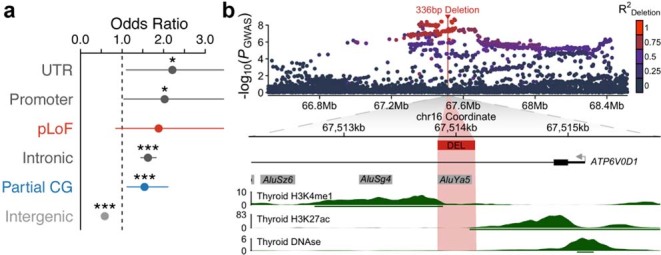
gnomAD-SV can augment disease association studies. **a**, Functional enrichments of 2,307 common SVs in strong linkage disequilibrium (*R*^2^ ≥ 0.8) with an SNV associated with a trait or disease in the GWAS catalogue or the UK Biobank^33,34^. Points represent odds ratios of SVs being in strong linkage disequilibrium with at least one GWAS-significant SNV among all SVs in strong linkage disequilibrium with at least one SNV (total *n* = 15,634 SVs). Single and triple asterisks correspond to nominal (*P* < 0.05) and Bonferroni-corrected (*P* < 0.0083) significance thresholds from a two-sided Fisher’s exact test, respectively. Bars represent 95% confidence intervals. Test statistics, SV counts, and *P* values are provided in Supplementary Table 6. **b**, Example locus at 16q22.1, where we identified a 336-bp deletion in strong linkage disequilibrium with SNVs significantly associated with hypothyroidism in the UK Biobank^34^. Top, the GWAS signal among genotyped SNVs in the UK Biobank, coloured by strength of linkage disequilibrium (Pearson’s *R*^2^ value) with the 336-bp deletion identified in gnomAD-SV. Bottom, the local genomic context of this deletion, which overlaps an annotated intronic *Alu* element near (<1 kb) the first exon of a highly constrained, thyroid-expressed gene, *ATP6V0D1*. The deletion lies amidst histone mark peaks commonly found at active enhancers (H3K27ac and H3K4me1) based on publicly available chromatin data from adult thyroid samples, a phenotype-relevant tissue^48^. Human *Alu* elements are known to frequently act as enhancers, and the sentinel hypothyroidism SNV from the UK Biobank GWAS is a significant expression-modifying variant (that is, eQTL) for *ATP6V0D1* and other nearby genes across many tissues, which indicates that the hypothyroidism risk haplotype modifies expression of *ATP6V0D1* and/or other genes, potentially through the deletion of an intronic enhancer^4,49^.

Most large-scale trait association studies have only considered SNVs in genome-wide association studies (GWAS). Taking advantage of the sample size and resolution of gnomAD-SV, we evaluated whether SNVs associated with human traits might be in linkage disequilibrium with SVs not directly genotyped in GWAS. We identified 15,634 common SVs (allele frequency >1%) in strong linkage disequilibrium (*R*^2^ ≥ 0.8) with at least one common short variant (Supplementary Fig. 7), 14.8% of which matched a reported association from the NHGRI-EBI GWAS catalogue or a recent analysis of 4,203 phenotypes in the UK Biobank^33,34^. Common SVs in linkage disequilibrium with GWAS variants were enriched for genic SVs across multiple functional categories (Supplementary Table 6), and included candidate SVs such as a deletion of a thyroid enhancer in the first intron of *ATP6V0D1* at a hypothyroidism-associated locus^34^ (Extended Data Fig. 7). We also identified matches for previously proposed causal SVs tagged by common SNVs, including pLoF deletions of *CFHR3* or *CFHR1* in nephropathies and of *LCE3B* or *LCE3C* in psoriasis^35,36^. These results demonstrate the value of imputing SVs into GWAS, and for the eventual unification of short variants and SVs in all trait association studies. Given the potential value of this resource, we have released these linkage disequilibrium maps in Supplementary Table 7.

**Fig. 6:**
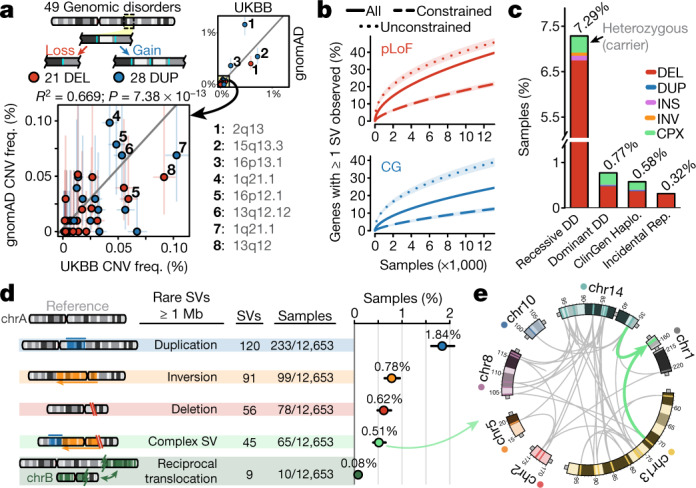
gnomAD-SV as a resource for clinical WGS interpretation. **a**, Comparison of carrier frequencies for 49 putatively disease-associated deletions (red) and duplications (blue) at genomic disorder loci between gnomAD-SV and microarray analyses in the UK Biobank (UKBB)^38^. Light bars indicate binomial 95% confidence intervals. Solid grey line represents linear best fit. **b**, At least one pLoF or copy-gain SV was detected in 36.9% and 23.7% of all autosomal genes, respectively. ‘Constrained’ and ‘unconstrained’ includes the least and most constrained 15% of all genes based on LOEUF^4^, respectively. **c**, Carrier rates for very rare (allele frequency < 0.1%) pLoF SVs in medically relevant genes across several gene lists^7,39,44^. SVs per category listed in Supplementary Table 9. **d**, Carrier rates for very large (≥1 Mb) rare autosomal SVs among 12,653 genomes. Bars represent binomial 95% confidence intervals. **e**, A complex SV involving at least 49 breakpoints and seven chromosomes (also see Extended Data Fig. 8). Teal arrows indicate insertion point into chromosome 1.

As genomic medicine advances towards diagnostic screening at sequence resolution, computational methods for variant discovery from WGS and population references for interpretation will become indispensable. One category of disease-associated SVs, recurrent CNVs mediated by homologous segmental duplications known as genomic disorders, are particularly important because they collectively represent a common cause of developmental disorders^37^. Accurate detection of large, repeat-mediated CNVs is thus crucial for WGS-based diagnostic testing as chromosomal microarray is the recommended first-tier diagnostic screen at present for unexplained developmental disorders^37^. Using gnomAD-SV, we evaluated our ability to detect genomic disorders in WGS data by calculating CNV carrier frequencies for 49 genomic disorders across 10,047 unrelated samples with no known neuropsychiatric disease and found that CNV carrier frequencies in gnomAD-SV were consistent with those reported from chromosomal microarray in the UK Biobank^38^ (*R*^2^ = 0.669; Pearson correlation test, *P* = 7.38 × 10^−13^) (Fig. 6a, Supplementary Table 8, Supplementary Fig. 20). The frequencies of carriers of genomic disorders did not vary significantly among populations, with the exception of duplications of *NPHP1* at 2q13, in which carrier frequencies in East Asian samples were up to 4.6-fold higher than in other populations, further highlighting the potential for variant interpretation to be confounded by the limited diversity of existing SV references (Supplementary Fig. 21).

**Extended Data Fig. 8:**
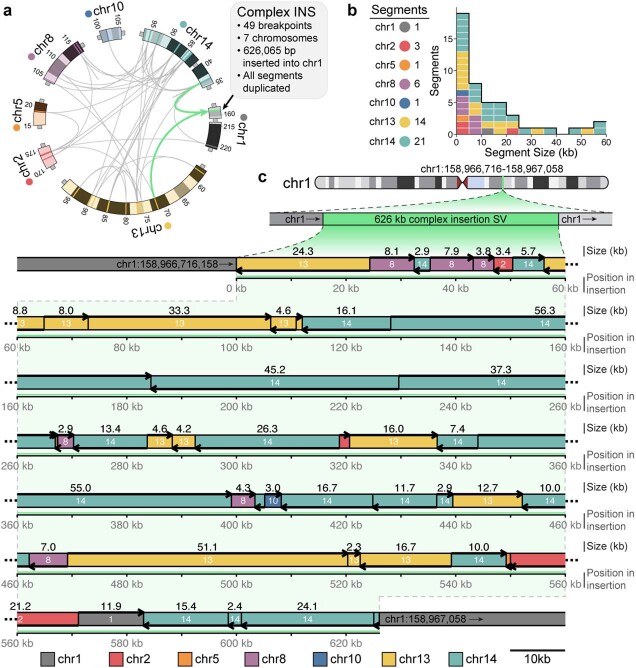
An extremely complex SV involving 49 breakpoints and seven chromosomes. A highly complex insertion rearrangement from gnomAD-SV in which 47 segments from six different chromosomes were duplicated and inserted into a single locus on chromosome 1, forming a 626,065 bp stretch of contiguous inserted sequence composed of shattered fragments. Given the involvement of multiple chromosomes, the signature of localized shattering, and the clustered breakpoints, we note that this rearrangement has several hallmarks of germline chromothripsis, which has been observed in healthy adults previously, albeit rarely^22^. However, unlike previous reports of germline chromothripsis, there are no apparent whole-chromosome translocations, and all segments were duplicated before being inserted in a compound manner into chromosome 1, potentially suggesting a replication-based repair mechanism. The exact origin of this rearrangement is unclear. **a**, Circos representation of all 49 breakpoints and seven chromosomes involved in this SV. Teal arrows indicate insertion point into chromosome 1. **b**, The median segment size was 8.4 kb. **c**, Linear representation of the rearranged inserted sequence. Colours correspond to chromosome of origin, and arrows indicate strandedness of the inserted sequence, relative to the GRCh37 reference.

In the context of variant interpretation, the current gnomAD-SV resource will permit a screening threshold of allele frequencies less than 0.1% when matching on ancestry to the populations sampled here, and allele frequencies less than 0.004% globally. In the current release, we catalogued at least one pLoF or copy-gain variant for 36.9% and 23.7% of all autosomal genes, respectively, and 490 genes with at least one homozygous pLoF SV (Fig. 6b, Extended Data Fig. 6e, Supplementary Fig. 22). We also benchmarked carrier rates for several categories of clinically relevant variants in gnomAD-SV. First, 0.32% of samples carried a very rare (allele frequency < 0.1%) SV resulting in pLoF of a gene for which incidental findings are clinically actionable, nearly half of which (that is, 0.13% of all samples) would meet diagnostic criteria as pathogenic or likely pathogenic based upon the American College of Medical Genetics (ACMG) recommendations^7^ (Fig. 6c). Second, 7.22% of individuals were heterozygous carriers of rare pLoF SVs in known recessive developmental disorder genes^39^. Third, we estimated that 3.8% of the general population (95% confidence interval of 3.2–4.6%) carries at least one very large (≥1 Mb) rare autosomal SV, roughly half of which (45.2%) were balanced or complex (Fig. 6d). Among these was an example of localized chromosome shattering involving at least 49 breakpoints, yet resulting in largely balanced products, reminiscent of chromothripsis, in an adult with no known severe disease or DNA repair defect^13,14,22^ (Fig. 6e, Extended Data Fig. 8). Collectively, these analyses highlight the potential of gnomAD-SV and WGS-based SV methods to augment disease-association studies and clinical interpretation across a broad spectrum of variant classes and study designs.

## Discussion

Human genetic research and clinical diagnostics are becoming increasingly invested in capturing the complete landscape of variation in individual genomes. Ambitious international initiatives to generate short-read WGS in many thousands of individuals from common disease cohorts have underwritten this goal^40,41^, and millions of genomes will be sequenced in the coming years from national biobanks^42,43^. A central challenge to these efforts will be the uniform analysis and interpretation of all variation accessible to WGS, particularly SVs, which are frequently invoked as a source of added value offered by WGS. Indeed, early WGS studies in cardiovascular disease and autism have been largely consistent in their analyses of short variants, but every study has differed in its analysis of SVs^18–20,40,41^. Thus, while ExAC and gnomAD have prompted remarkable advances in medical and population genetics for short variants, the same gains have not yet been realized for SVs. Although gnomAD-SV is not exhaustively comprehensive, it was derived from WGS methods and a reference genome that match those currently used in many research and clinical settings, which will help to facilitate the eventual standardization of SV discovery, analysis, and interpretation across studies.

Most foundational assumptions about human genetic variation were consistent between SVs and short variants in gnomAD, most notably that SVs segregate stably on haplotypes in the population and experience selection commensurate with their predicted biological consequences. This study also spotlights unique aspects of SVs, such as their remarkable mutational diversity, their varied functional effects on coding sequence, and the intense selection against large and complex SVs. Our analyses also demonstrate that gene-altering effects of SVs beyond pLoF are remarkably similar to the mutational constraints of SNVs, and that SNV constraint metrics are not specific to haploinsufficiency but underlie a general intolerance to alterations of both gene dosage and structure. Beyond genes, we uncovered widespread but modest selection against noncoding dosage alterations of many families of *cis*-regulatory elements. This study represents one of the largest empirical assessments of noncoding dosage sensitivity in humans, and underscores that: (1) few—if any—classes of noncoding *cis*-regulatory variants are likely to experience selection as strong as protein-truncating variants; (2) sequence conservation is unsurprisingly one of the strongest features associated with selection against noncoding SVs; and (3) current WGS sample sizes are vastly underpowered to identify individual constrained functional elements in the noncoding genome.

The value of the multi-algorithm ensemble approach and deep WGS is evident in the improved sensitivity of SV detection in gnomAD-SV. However, short-read WGS remains limited by comparison to emerging long-read technologies^21^. Given that short-read WGS is blind to a disproportionate fraction of repeat-mediated SVs and small insertions by comparison to long-read methods, this study certainly underestimates the true mutation rates within such hypermutable regions. Similarly, although our approach involves extensive methods to resolve complex SV alleles, some variants such as high-copy-state MCNVs often involve complicated haplotype configurations, and we expect that emerging de novo assembly and graph-based genome representations will greatly expand our knowledge of such SVs^21,23^. Nonetheless, 92.7% of all known autosomal protein-coding nucleotides are not localized to simple- or low-copy repeats, and therefore we expect that the catalogues of SVs accessible to short-read WGS across large populations like gnomAD-SV will capture a majority of the most interpretable gene-disrupting SVs in humans.

The scale of short-read WGS datasets currently in production has magnified the need for publicly available SV resources, and gnomAD-SV represents an initial effort to fill this void. Although these data remain insufficient to derive accurate estimates of gene-level constraint, sequence-specific mutation rates, and intolerance to noncoding SVs, they provide a step towards these goals and reinforce the value of data sharing and harmonized analyses of aggregated genomic data sets. These data have been made available without restrictions on reuse (https://gnomad.broadinstitute.org), and this resource will catalyse new discoveries in basic research while providing immediate clinical utility for the interpretation of rare structural rearrangements across human populations.

### Reporting summary

Further information on research design is available in the Nature Research Reporting Summary linked to this paper.

## Online content

Any methods, additional references, Nature Research reporting summaries, source data, extended data, supplementary information, acknowledgements, peer review information; details of author contributions and competing interests; and statements of data and code availability are available at 10.1038/s41586-020-2287-8.

## Supplementary information

Supplementary Information This file contains Supplementary Figures 1-24, Supplementary Notes, Supplementary Methods, and additional references.

Reporting Summary

Peer Review File Reviewer reports and authors' response from the peer review of this Article at Nature.

Supplementary Tables This file contains Supplementary Tables 1-9.

## Data Availability

All gnomAD-SV site-frequency data for appropriately consented samples (*n* = 10,847) have been distributed in VCF and BED format via the gnomAD browser (https://gnomad.broadinstitute.org/downloads/), as well as from NCBI dbVar under accession nstd166. Furthermore, these SVs have been integrated directly into the gnomAD browser^8^. The architecture of the gnomAD browser is described in the main gnomAD study^4^, as well as instructions for how to access and query the data hosted therein.

## Code availability

The gnomAD-SV discovery pipeline is publicly available via a series of methods configured for the FireCloud/Terra platform (https://portal.firecloud.org/#methods) under the methods namespace ‘Talkowski-SV’. The svtk software package used extensively in the gnomAD-SV discovery pipeline is publicly available via GitHub (https://github.com/talkowski-lab/svtk). Most custom scripts used in the production and/or analysis of the gnomAD-SV dataset are publicly available via GitHub (https://github.com/talkowski-lab/gnomad-sv-pipeline). All code is made available under the MIT license, unless stated otherwise.
