# Author Correction: A structural variation reference for medical and population genetics

**DOI:** 10.1038/s41586-020-03176-6

**Published:** 2021-02-03

**Authors:** Ryan L. Collins, Harrison Brand, Konrad J. Karczewski, Xuefang Zhao, Jessica Alföldi, Laurent C. Francioli, Amit V. Khera, Chelsea Lowther, Laura D. Gauthier, Harold Wang, Nicholas A. Watts, Matthew Solomonson, Anne O’Donnell-Luria, Alexander Baumann, Ruchi Munshi, Mark Walker, Christopher W. Whelan, Yongqing Huang, Ted Brookings, Ted Sharpe, Matthew R. Stone, Elise Valkanas, Jack Fu, Grace Tiao, Kristen M. Laricchia, Valentin Ruano-Rubio, Christine Stevens, Namrata Gupta, Caroline Cusick, Lauren Margolin, Genome Aggregation Database Production Team, Jessica Alföldi, Irina M. Armean, Eric Banks, Louis Bergelson, Kristian Cibulskis, Ryan L. Collins, Kristen M. Connolly, Miguel Covarrubias, Beryl Cummings, Mark J. Daly, Stacey Donnelly, Yossi Farjoun, Steven Ferriera, Laurent Francioli, Stacey Gabriel, Laura D. Gauthier, Jeff Gentry, Namrata Gupta, Thibault Jeandet, Diane Kaplan, Konrad J. Karczewski, Kristen M. Laricchia, Christopher Llanwarne, Eric V. Minikel, Ruchi Munshi, Benjamin M. Neale, Sam Novod, Anne H. O’Donnell-Luria, Nikelle Petrillo, Timothy Poterba, David Roazen, Valentin Ruano-Rubio, Andrea Saltzman, Kaitlin E. Samocha, Molly Schleicher, Cotton Seed, Matthew Solomonson, Jose Soto, Grace Tiao, Kathleen Tibbetts, Charlotte Tolonen, Christopher Vittal, Gordon Wade, Arcturus Wang, Qingbo Wang, James S. Ware, Nicholas A. Watts, Ben Weisburd, Nicola Whiffin, Genome Aggregation Database Consortium, Carlos A. Aguilar Salinas, Tariq Ahmad, Christine M. Albert, Diego Ardissino, Gil Atzmon, John Barnard, Laurent Beaugerie, Emelia J. Benjamin, Michael Boehnke, Lori L. Bonnycastle, Erwin P. Bottinger, Donald W. Bowden, Matthew J. Bown, John C. Chambers, Juliana C. Chan, Daniel Chasman, Judy Cho, Mina K. Chung, Bruce Cohen, Adolfo Correa, Dana Dabelea, Mark J. Daly, Dawood Darbar, Ravindranath Duggirala, Josée Dupuis, Patrick T. Ellinor, Roberto Elosua, Jeanette Erdmann, Tõnu Esko, Martti Färkkilä, Jose Florez, Andre Franke, Gad Getz, Benjamin Glaser, Stephen J. Glatt, David Goldstein, Clicerio Gonzalez, Leif Groop, Christopher Haiman, Craig Hanis, Matthew Harms, Mikko Hiltunen, Matti M. Holi, Christina M. Hultman, Mikko Kallela, Jaakko Kaprio, Sekar Kathiresan, Bong-Jo Kim, Young Jin Kim, George Kirov, Jaspal Kooner, Seppo Koskinen, Harlan M. Krumholz, Subra Kugathasan, Soo Heon Kwak, Markku Laakso, Terho Lehtimäki, Ruth J. F. Loos, Steven A. Lubitz, Ronald C. W. Ma, Daniel G. MacArthur, Jaume Marrugat, Kari M. Mattila, Steven McCarroll, Mark I. McCarthy, Dermot McGovern, Ruth McPherson, James B. Meigs, Olle Melander, Andres Metspalu, Benjamin M. Neale, Peter M. Nilsson, Michael C. O’Donovan, Dost Ongur, Lorena Orozco, Michael J. Owen, Colin N. A. Palmer, Aarno Palotie, Kyong Soo Park, Carlos Pato, Ann E. Pulver, Nazneen Rahman, Anne M. Remes, John D. Rioux, Samuli Ripatti, Dan M. Roden, Danish Saleheen, Veikko Salomaa, Nilesh J. Samani, Jeremiah Scharf, Heribert Schunkert, Moore B. Shoemaker, Pamela Sklar, Hilkka Soininen, Harry Sokol, Tim Spector, Patrick F. Sullivan, Jaana Suvisaari, E. Shyong Tai, Yik Ying Teo, Tuomi Tiinamaija, Ming Tsuang, Dan Turner, Teresa Tusie-Luna, Erkki Vartiainen, Marquis P. Vawter, James S. Ware, Hugh Watkins, Rinse K. Weersma, Maija Wessman, James G. Wilson, Ramnik J. Xavier, Kent D. Taylor, Henry J. Lin, Stephen S. Rich, Wendy S. Post, Yii-Der Ida Chen, Jerome I. Rotter, Chad Nusbaum, Anthony Philippakis, Eric Lander, Stacey Gabriel, Benjamin M. Neale, Sekar Kathiresan, Mark J. Daly, Eric Banks, Daniel G. MacArthur, Michael E. Talkowski

**Affiliations:** 1 Program in Medical and Population Genetics, Broad Institute of MIT and Harvard, Cambridge, MA USA; 2 Center for Genomic Medicine, Massachusetts General Hospital, Boston, MA USA; 3 Division of Medical Sciences, Harvard Medical School, Boston, MA USA; 4 Department of Neurology, Massachusetts General Hospital and Harvard Medical School, Boston, MA USA; 5 Analytical and Translational Genetics Unit, Massachusetts General Hospital, Boston, MA USA; 6 Department of Medicine, Harvard Medical School, Boston, MA USA; 7 Data Science Platform, Broad Institute of MIT and Harvard, Cambridge, MA USA; 8 The Institute for Translational Genomics and Population Sciences, Department of Pediatrics, Los Angeles Biomedical Research Institute at Harbor-UCLA Medical Center, Torrance, CA USA; 9 Center for Public Health Genomics, University of Virginia, Charlottesville, VA USA; 10 Johns Hopkins University School of Medicine, Baltimore, MD USA; 11 Department of Systems Biology, Harvard Medical School, Boston, MA USA; 12 Department of Biology, MIT, Cambridge, MA USA; 13 Stanley Center for Psychiatric Research, Broad Institute of MIT and Harvard, Cambridge, MA USA; 14 Division of Cardiology, Massachusetts General Hospital, Boston, MA USA; 155 Present Address: Cellarity Inc., Cambridge, MA USA; 156 Present Address: Centre for Population Genomics, Garvan Institute of Medical Research, and UNSW Sydney, Sydney, Australia; 157 Present Address: Centre for Population Genomics, Murdoch Children’s Research Institute, Melbourne, Australia; 15 Analytic and Translational Genetics Unit, Massachusetts General Hospital, Boston, MA USA; 16 European Molecular Biology Laboratory, European Bioinformatics Institute, Wellcome Genome Campus, Hinxton, Cambridge, UK; 17 Genomics Platform, Broad Institute of MIT and Harvard, Cambridge, MA USA; 18 Broad Genomics, Broad Institute of MIT and Harvard, Cambridge, MA USA; 19 Division of Genetics and Genomics, Boston Children’s Hospital, Boston, MA USA; 20 Department of Pediatrics, Harvard Medical School, Boston, MA USA; 21 Wellcome Sanger Institute, Wellcome Genome Campus, Hinxton, Cambridge, UK; 22 National Heart & Lung Institute and MRC London Institute of Medical Sciences, Imperial College London, London, UK; 23 Cardiovascular Research Centre, Royal Brompton & Harefield Hospitals NHS Trust, London, UK; 24 Unidad de Investigacion de Enfermedades Metabolicas, Instituto Nacional de Ciencias Medicas y Nutricion, Mexico City, Mexico; 25 Peninsula College of Medicine and Dentistry, Exeter, UK; 26 Division of Preventive Medicine, Brigham and Women’s Hospital, Boston, MA USA; 27 Division of Cardiovascular Medicine, Brigham and Women’s Hospital and Harvard Medical School, Boston, MA USA; 28 Department of Cardiology, University Hospital, Parma, Italy; 29 Department of Biology, Faculty of Natural Sciences, University of Haifa, Haifa, Israel; 30 Department of Medicine, Albert Einstein College of Medicine, Bronx, NY USA; 31 Department of Genetics, Albert Einstein College of Medicine, Bronx, NY USA; 32 Department of Quantitative Health Sciences, Lerner Research Institute, Cleveland Clinic, Cleveland, OH USA; 33 Gastroenterology Department, Sorbonne Université, APHP, Saint Antoine Hospital, Paris, France; 34 Framingham Heart Study, National Heart, Lung, & Blood Institute and Boston University, Framingham, MA USA; 35 Department of Medicine, Boston University School of Medicine, Boston, Massachusetts, USA; 36 Department of Epidemiology, Boston University School of Public Health, Boston, Massachusetts, USA; 37 Department of Biostatistics, Center for Statistical Genetics, University of Michigan, Ann Arbor, MI USA; 38 National Human Genome Research Institute, National Institutes of Health, Bethesda, MD USA; 39 The Charles Bronfman Institute for Personalized Medicine, Icahn School of Medicine at Mount Sinai, New York, NY USA; 40 Department of Biochemistry, Wake Forest School of Medicine, Winston-Salem, NC USA; 41 Center for Genomics and Personalized Medicine Research, Wake Forest School of Medicine, Winston-Salem, NC USA; 42 Center for Diabetes Research, Wake Forest School of Medicine, Winston-Salem, NC USA; 43 Department of Cardiovascular Sciences, University of Leicester, Leicester, UK; 44 Department of Cardiovascular Sciences and NIHR Leicester Biomedical Research Centre, University of Leicester, Leicester, UK; 45 Department of Epidemiology and Biostatistics, Imperial College London, London, UK; 46 Department of Cardiology, Ealing Hospital NHS Trust, Southall, UK; 47 Imperial College Healthcare NHS Trust, Imperial College London, London, UK; 48 Department of Medicine and Therapeutics, The Chinese University of Hong Kong, Hong Kong, China; 49 Program for Neuropsychiatric Research, McLean Hospital, Belmont, MA USA; 50 Department of Medicine, University of Mississippi Medical Center, Jackson, MI USA; 51 Department of Epidemiology, Colorado School of Public Health, Aurora, CO USA; 52 Department of Pharmacology, University of Illinois at Chicago, Chicago, IL USA; 53 Department of Medicine, University of Illinois at Chicago, Chicago, IL USA; 54 Department of Genetics, Texas Biomedical Research Institute, San Antonio, TX USA; 55 Department of Biostatistics, Boston University School of Public Health, Boston, MA USA; 56 Cardiac Arrhythmia Service, Cardiovascular Research Center, Massachusetts General Hospital, Boston, MA USA; 57 Cardiovascular Epidemiology and Genetics, Hospital del Mar Medical Research Institute (IMIM), Barcelona, Catalonia Spain; 58 Centro de Investigación en Red en Enfermedades Cardiovasculares (CIBERCV), Barcelona, Catalonia Spain; 59 Department of Medicine, Medical School, University of Vic-Central University of Catalonia, Vic, Catalonia Spain; 60 Institute for Cardiogenetics, University of Lübeck, Lübeck, Germany; 61 DZHK (German Research Centre for Cardiovascular Research), partner site Hamburg/Lübeck/Kiel, Lübeck, Germany; 62 University Heart Center, Lübeck, Lübeck, Germany; 63 Estonian Genome Center, Institute of Genomics, University of Tartu, Tartu, Estonia; 64 Clinic of Gastroenterology, Helsinki University Hospital, Helsinki University, Helsinki, Finland; 65 Diabetes Unit, Massachusetts General Hospital, Boston, MA USA; 66 Center for Genomic Medicine, Massachusetts General Hospital, Boston, MA USA; 67 Program in Metabolism, Broad Institute of MIT and Harvard, Cambridge, MA USA; 68 Institute of Clinical Molecular Biology (IKMB), Christian-Albrechts-University of Kiel, Kiel, Germany; 69 Bioinformatics Consortium, Massachusetts General Hospital, Boston, MA USA; 70 Department of Pathology, Massachusetts General Hospital, Boston, MA USA; 71 Cancer Center, Massachusetts General Hospital, Boston, MA USA; 72 Cancer Genome Computational Analysis Group, Broad Institute of MIT and Harvard, Cambridge, MA USA; 73 Endocrinology and Metabolism Department, Hadassah-Hebrew University Medical Center, Jerusalem, Israel; 74 Department of Psychiatry and Behavioral Sciences, SUNY Upstate Medical University, Syracuse, NY USA; 75 Institute for Genomic Medicine, Columbia University Medical Center, Hammer Health Sciences, New York, NY USA; 76 Department of Genetics and Development, Columbia University Medical Center, Hammer Health Sciences, New York, NY USA; 77 Centro de Investigacion en Salud Poblacional, Instituto Nacional de Salud Publica, Cuernavaca, Mexico; 78 Genomics, Diabetes and Endocrinology, Lund University, Lund, Sweden; 79 Institute for Molecular Medicine Finland (FIMM), HiLIFE, University of Helsinki, Helsinki, Finland; 80 Lund University Diabetes Centre, Malmö, Sweden; 81 Human Genetics Center, University of Texas Health Science Center at Houston, Houston, TX USA; 82 Department of Neurology, Columbia University, New York, NY USA; 83 Institute of Genomic Medicine, Columbia University, New York, NY USA; 84 Institute of Biomedicine, University of Eastern Finland, Kuopio, Finland; 85 Department of Psychiatry, Helsinki University Central Hospital, Lapinlahdentie, Helsinki, Finland; 86 Department of Medical Epidemiology and Biostatistics, Karolinska Institutet, Stockholm, Sweden; 87 Icahn School of Medicine at Mount Sinai, New York, NY USA; 88 Department of Neurology, Helsinki University Central Hospital, Helsinki, Finland; 89 Department of Public Health, Faculty of Medicine, University of Helsinki, Helsinki, Finland; 90 Cardiovascular Disease Initiative, Broad Institute of MIT and Harvard, Cambridge, MA USA; 91 Center for Genome Science, Korea National Institute of Health, Chungcheongbuk-do, South Korea; 92 MRC Centre for Neuropsychiatric Genetics & Genomics, Cardiff University School of Medicine, Cardiff, USA; 93 Department of Health, National Institute for Health and Welfare (THL), Helsinki, Finland; 94 Section of Cardiovascular Medicine, Department of Internal Medicine, Yale School of Medicine, Connecticut Center for Outcomes Research and Evaluation, Yale New Haven Hospital, New Haven, CT USA; 95 Division of Pediatric Gastroenterology, Emory University School of Medicine, Atlanta, GA USA; 96 Department of Internal Medicine, Seoul National University Hospital, Seoul, South Korea; 97 Institute of Clinical Medicine, The University of Eastern Finland, Kuopio, Finland; 98 Institute of Clinical Medicine Neurology, Kuopio University Hospital, Kuopio, Finland; 99 Department of Clinical Chemistry, Fimlab Laboratories and Finnish Cardiovascular Research Center-Tampere, Faculty of Medicine and Health Technology, Tampere University, Tampere, Finland; 100 The Mindich Child Health and Development Institute, Icahn School of Medicine at Mount Sinai, New York, NY USA; 101 Li Ka Shing Institute of Health Sciences, The Chinese University of Hong Kong, Hong Kong, China; 102 Hong Kong Institute of Diabetes and Obesity, The Chinese University of Hong Kong, Hong Kong, China; 103 Cardiovascular Research REGICOR Group, Hospital del Mar Medical Research Institute (IMIM), Barcelona, Catalonia Spain; 104 Department of Genetics, Harvard Medical School, Boston, MA USA; 105 Oxford Centre for Diabetes, Endocrinology and Metabolism, University of Oxford, Churchill Hospital, Headington, Oxford, UK; 106 Wellcome Centre for Human Genetics, University of Oxford, Oxford, UK; 107 Oxford NIHR Biomedical Research Centre, Oxford University Hospitals NHS Foundation Trust, John Radcliffe Hospital, Oxford, UK; 108 F Widjaja Foundation Inflammatory Bowel and Immunobiology Research Institute, Cedars-Sinai Medical Center, Los Angeles, CA USA; 109 Atherogenomics Laboratory, University of Ottawa Heart Institute, Ottawa, Canada; 110 Division of General Internal Medicine, Massachusetts General Hospital, Boston, MA USA; 111 Department of Clinical Sciences, University Hospital Malmo Clinical Research Center, Lund University, Malmo, Sweden; 112 Department of Clinical Sciences, Lund University, Skane University Hospital, Malmo, Sweden; 113 Instituto Nacional de Medicina Genómica (INMEGEN), Mexico City, Mexico; 114 Medical Research Institute, Ninewells Hospital and Medical School, University of Dundee, Dundee, UK; 115 Department of Molecular Medicine and Biopharmaceutical Sciences, Graduate School of Convergence Science and Technology, Seoul National University, Seoul, South Korea; 116 Department of Psychiatry, Keck School of Medicine at the University of Southern California, Los Angeles, CA USA; 117 Department of Psychiatry and Behavioral Sciences, Johns Hopkins University School of Medicine, Baltimore, MA USA; 118 Division of Genetics and Epidemiology, Institute of Cancer Research, London, USA; 119 Research Unit of Clinical Neuroscience, University of Oulu, Oulu, Finland; 120 Research Center, Montreal Heart Institute, Montreal, Quebec Canada; 121 Department of Medicine, Faculty of Medicine, Université de Montréal, Montreal, Quebec Canada; 122 Department of Biomedical Informatics, Vanderbilt University Medical Center, Nashville, TN USA; 123 Department of Medicine, Vanderbilt University Medical Center, Nashville, TN USA; 124 Department of Biostatistics and Epidemiology, Perelman School of Medicine at the University of Pennsylvania, Philadelphia, PA USA; 125 Department of Medicine, Perelman School of Medicine at the University of Pennsylvania, Philadelphia, PA USA; 126 Center for Non-Communicable Diseases, Karachi, Pakistan; 127 National Institute for Health and Welfare, Helsinki, Finland; 128 Deutsches Herzzentrum München, Munich, Germany; 129 Technische Universität München, Munich, Germany; 130 Division of Cardiovascular Medicine, Nashville VA Medical Center and Vanderbilt University, School of Medicine, Nashville, TN USA; 131 Department of Psychiatry, Icahn School of Medicine at Mount Sinai, New York, NY USA; 132 Department of Genetics and Genomic Sciences, Icahn School of Medicine at Mount Sinai, New York, NY USA; 133 Institute for Genomics and Multiscale Biology, Icahn School of Medicine at Mount Sinai, New York, NY USA; 134 Institute of Clinical Medicine Neurology, University of Eastern Finland, Kuopio, Finland; 135 Department of Twin Research and Genetic Epidemiology, King’s College London, London, UK; 136 Departments of Genetics and Psychiatry, University of North Carolina, Chapel Hill, NC USA; 137 Saw Swee Hock School of Public Health, National University of Singapore, National University Health System, Singapore, Singapore; 138 Department of Medicine, Yong Loo Lin School of Medicine, National University of Singapore, Singapore, Singapore; 139 Duke-NUS Graduate Medical School, Singapore, Singapore; 140 Life Sciences Institute, National University of Singapore, Singapore, Singapore; 141 Department of Statistics and Applied Probability, National University of, Singapore, Singapore; 142 Folkhälsan Institute of Genetics, Folkhälsan Research Center, Helsinki, Finland; 143 HUCH Abdominal Center, Helsinki University Hospital, Helsinki, Finland; 144 Department of Psychiatry, Center for Behavioral Genomics, University of California, San Diego, CA USA; 145 Institute of Genomic Medicine, University of California, San Diego, CA USA; 146 Juliet Keidan Institute of Pediatric Gastroenterology, Shaare Zedek Medical Center, The Hebrew University of Jerusalem, Jerusalem, Israel; 147 Instituto de Investigaciones Biomédicas UNAM, Mexico City, Mexico; 148 Instituto Nacional de Ciencias Médicas y Nutrición Salvador Zubirán, Mexico City, Mexico; 149 Radcliffe Department of Medicine, University of Oxford, Oxford, UK; 150 Department of Gastroenterology and Hepatology, University of Groningen and University Medical Center Groningen, Groningen, The Netherlands; 151 Department of Physiology and Biophysics, University of Mississippi Medical Center, Jackson, MS USA; 152 Program in Infectious Disease and Microbiome, Broad Institute of MIT and Harvard, Cambridge, MA USA; 153 Center for Computational and Integrative Biology, Massachusetts General Hospital, Boston, MA USA; 154 Department of Psychiatry & Human Behavior, University of California Irvine, Irvine, CA USA

**Keywords:** Genome informatics, Chromosome abnormality, Structural variation, Genomics, Mutation

Correction to: *Nature* 10.1038/s41586-020-2287-8Published online 27 May 2020

In this Article, author Marquis P. Vawter was missing from the Genome Aggregation Database Consortium list. They are associated with the affiliation: ‘Department of Psychiatry & Human Behavior, University of California Irvine, Irvine, CA, USA’, and contributed to the generation of the primary data incorporated into the gnomAD resource. In addition, the following statement should have been included in the Acknowledgements section: ‘L.C.F. was supported by the Swiss National Science Foundation (Advanced Postdoc.Mobility 177853).’ The original Article has been corrected online.

