# Supplementary material for: A structural variation reference for medical and population genetics
Source: Nature. 2020 May 27;581(7809):444–51. doi: 10.1038/s41586-020-2287-8 (PMC7334194; doi:10.1038/s41586-020-2287-8)
Supplement: Supplementary file 2 — Reporting Summary [file 41586_2020_2287_MOESM2_ESM.pdf]

nature research

Corresponding author(s): Michael Talkowski, Ph.D.

Last updated by author(s): Feb 27, 2020

Reporting Summary

Nature Research wishes to improve the reproducibility of the work that we publish. This form provides structure for consistency and transparency in reporting. For further information on Nature Research policies, see Authors & Referees and the Editorial Policy Checklist.

Statistics

For all statistical analyses, confirm that the following items are present in the figure legend, table legend, main text, or Methods section.

n/a	Confirmed	
☒	☐	The exact sample size (n) for each experimental group/condition, given as a discrete number and unit of measurement
☒	☐	A statement on whether measurements were taken from distinct samples or whether the same sample was measured repeatedly
☐	☒	The statistical test(s) used AND whether they are one- or two-sided<br>*Only common tests should be described solely by name; describe more complex techniques in the Methods section.*
☐	☒	A description of all covariates tested
☐	☒	A description of any assumptions or corrections, such as tests of normality and adjustment for multiple comparisons
☐	☒	A full description of the statistical parameters including central tendency (e.g. means) or other basic estimates (e.g. regression coefficient) AND variation (e.g. standard deviation) or associated estimates of uncertainty (e.g. confidence intervals)
☐	☒	For null hypothesis testing, the test statistic (e.g. F, t, r) with confidence intervals, effect sizes, degrees of freedom and P value noted<br>*Give P values as exact values whenever suitable.*
☒	☐	For Bayesian analysis, information on the choice of priors and Markov chain Monte Carlo settings
☒	☐	For hierarchical and complex designs, identification of the appropriate level for tests and full reporting of outcomes
☐	☒	Estimates of effect sizes (e.g. Cohen's d, Pearson's r), indicating how they were calculated

Our web collection on statistics for biologists contains articles on many of the points above.

Software and code

Policy information about availability of computer code

Data collection	All software used for data collection is described in the Methods. All software is publicly available, and all custom software developed in this study has been released under the MIT license. Details on code access are provided in Supplementary Information.
Data analysis	All software used for data analysis is described in the Methods. All software is publicly available, and all custom software developed in this study has been released under the MIT license. Details on code access are provided in Supplementary Information.

For manuscripts utilizing custom algorithms or software that are central to the research but not yet described in published literature, software must be made available to editors/reviewers. We strongly encourage code deposition in a community repository (e.g. GitHub). See the Nature Research guidelines for submitting code & software for further information.

Data

Policy information about availability of data

All manuscripts must include a data availability statement. This statement should provide the following information, where applicable:

- Accession codes, unique identifiers, or web links for publicly available datasets
- A list of figures that have associated raw data
- A description of any restrictions on data availability

The entire gnomAD-SV reference map, from which all conclusions in the study are drawn, has been made publicly accessible through multiple sources. See the Data Availability Statement in the Supplementary Information for more instructions on data access.

Field-specific reporting

Please select the one below that is the best fit for your research. If you are not sure, read the appropriate sections before making your selection.

☒ Life sciences ☐ Behavioural & social sciences ☐ Ecological, evolutionary & environmental sciences

For a reference copy of the document with all sections, see nature.com/documents/nr-reporting-summary-flat.pdf

Life sciences study design

All studies must disclose on these points even when the disclosure is negative.

Sample size	No statistical analyses were performed to predetermine sample size. Sample size was dictated by the total number of human whole-genome sequences available with appropriate consent for genetic data aggregation and variant site & frequency release.
Data exclusions	A minority of samples were excluded for various analyses. Where applicable, these exclusions are detailed in the main manuscript, Methods, and Supplementary Information
Replication	No replication was attempted.
Randomization	No randomization was necessary as most analyses were performed on all available samples.
Blinding	No blinding was performed, as no comparisons between subsets of samples were conducted.

Reporting for specific materials, systems and methods

We require information from authors about some types of materials, experimental systems and methods used in many studies. Here, indicate whether each material, system or method listed is relevant to your study. If you are not sure if a list item applies to your research, read the appropriate section before selecting a response.

Materials & experimental systems

n/a	Involved in the study	
☒	☐	Antibodies
☒	☐	Eukaryotic cell lines
☒	☐	Palaeontology
☒	☐	Animals and other organisms
☐	☒	Human research participants
☒	☐	Clinical data

Methods

n/a	Involved in the study	
☒	☐	ChIP-seq
☒	☐	Flow cytometry
☒	☐	MRI-based neuroimaging

Human research participants

Policy information about studies involving human research participants

Population characteristics	The samples analyzed in this study were aggregated across numerous population genetic and complex disease association studies. This aggregation is described in detail in the Methods, and in Karczewski et al., 2019.
Recruitment	Samples were not recruited directly by this study. Data aggregation is described in detail in the Methods, and in Karczewski et al., 2019.
Ethics oversight	This study was overseen by the Broad Institute's Office of Research Subject Protection and the Partners Human Research Committee, and was given a determination of Not Human Subjects Research.

Note that full information on the approval of the study protocol must also be provided in the manuscript.
