# Supplementary material for: A structural variation reference for medical and population genetics
Source: Nature. 2020 May 27;581(7809):444–51. doi: 10.1038/s41586-020-2287-8 (PMC7334194; doi:10.1038/s41586-020-2287-8)
Supplement: Supplementary file 3 — Reviewer reports and authors' response from the peer review of this Article at Nature. [file 41586_2020_2287_MOESM3_ESM.pdf]

Peer Review File

Manuscript Title: A structural variation reference for medical and population genetics

Reviewer Comments & Author Rebuttals

Reviewer Reports on the Initial Version:

Referee #1 (Remarks to the Author):

This paper, entitled "A global structural variation reference for medical and population genetics" by Collins et al. presented gnomAD-SV, a supplemental addition to gnomAD that includes SV variations of ~15,000 samples from different populations based on deep WGS sequencing. The authors demonstrated that gnomAD-SV correlated with SV calls from the 1000 Genomes Project (1KG) and also covered additional novel SVs. The authors further reaffirm that many of their inversion signatures correspond to complex SV events, rather than represent simple inversions, as others have shown previously.

A few concerns arise as I read through this manuscript. First, the null distribution simulation of SVs, which the authors used to evaluate SV selective pressure, is, by the authors' own admission, imperfect. However, they don't give any indication about what other factors might be missing, or whether the model in its current state is of sufficient quality for the clinical applications they clearly are aiming for. I believe the authors need to demonstrate that this current model is of sufficient quality, or at least explain what this model needs for improvement, and why it is difficult to achieve.

Second, there is a recapitulation of known disease-associated SVs in the paper, but no demonstration of gnomAD-SV's usefulness at identifying novel disease-associated SVs. An analysis of SVs predicted in gnomAD-SV, but not elsewhere, accompanied with an experimental validation, would be most helpful here.

Third, the authors release their data as hg19 only. Given the age of the hg19 reference, and the fact that recent and future human genome projects will undoubtedly use more recent reference builds, I think the utility of gnomAD-SV will be increased if the authors also release an hg38 version. Other publications have provided data generated against both reference builds to offer broad applicability of their data.

Fourth, a major issue is the lack of direct experimental validation of their results, especially for complex variants and variants found by a single technology. Any new variants identified without providing any direct evidence for these SVs seriously undermines confidence in these novel SVs. Instead, they rely on an analysis of Mendelian concordance, and a very limited set of PacBio sequencing data (4 samples; 0.03% of the sample set). Also, it would be helpful if the authors provide detailed results in the tables/figures for their purported long-read support for up to 88.1% of SVs predicted from short-read WGS.
Finally, there seems to be a lack of a defined SV calling pipeline provided by the authors that others in the field can use for calling SVs in similar cohorts. Their own pipeline appears to be unpublished and I do not believe it is currently available as a tool for other researchers.

Other comments:
In the text, the authors state that the gnomAD-SV resource will be made available without restrictions on reuse, and will be integrated directly into the gnomAD Browser. I wonder whether the authors have an estimated date for when the SV data will be publically available in the browser.

The rate of pathogenic SVs reported in the text didn't match what is reported in the figures. In the

Summary (Line 59), Introduction (Line 105) and Results (Line 347) section, the rate was estimated as 0.24. However the rate was reported as 0.4% in the Figure 6C. In addition, how did the authors estimate this rate? I recommend the authors provide some details on the underlying methods.

The description for the pLoF is not consistent in the text. The rare pLoF SVs were reported as "at least 25%" in the Summary (Line 58), ", "approximately 25%" in the Introduction (Line 105) and "up to 25%" in the Results (Line 246). Furthermore, it's not clear to me how the authors came up with this number (25%). Following the main text, I estimated it as 21.7% (=5 rare pLoF SVs/(5 rare pLoF SVs +18 rare pLoF SNV/Indels)). Can the authors please clarify this result and its underlying methods?

Line 115: "the samples across population genetics and complex disease association studies". Can the authors please provide more details, i.e. the numbers of patient samples and the type of diseases of the patient samples used in this study?

Line 136: "BNDs substantially inflated the variant count, were enriched in false positives". How do the authors determine whether one BND is false positive or not?

Line 150: "97.8% sensitivity to detect large CNVs (>40kb) previously reported from microarrays in 1,893 individuals (ref 25)". The Ref 25 paper used 10,220 samples from 2,591 ASD families for CNV study. What the criteria the authors used to select those 1,893 samples for the comparison here? Also can the authors provide the exact CNV numbers to calculate 97.8%? Since the Ref 25 was an ASD study, I would like to know whether this current study included any ASD samples and whether those 97.8% CNVs were enriched in the ASD samples. If no ASD samples were included in this study, can the authors explain why 97.8% of ASD CNVs can be detected in non-ASD samples?

Line 155: The authors compared their results with 1KG and found 87.4% of SVs are novel. I would like to know how many SVs reported in 1KGP can be detected in your results. The precision and recall rate should be provided here.

Lines 189-190: "Among canonical SVs, deletions were collectively more rare than other classes (P < 1x10-100; one-sided Wilcoxon Test; Supplementary Figure 8)". While in Figure 1C, the authors clearly showed that deletions are the most abundant SV class. Can the authors please address this discrepancy?

Line 206: Why is the mutation rate of 0.35 de novo SVs per generation in this study more than 2-fold the rate of the 519 quartets (~0.15)? Can the authors provide the age distribution of the samples?

Line 243: The citation to the Extended Data Figure 2e-h should be corrected as Extended Data Figure 3e-h

Lines 309-311: "…duplications of NPHP1 at 2q13, where carrier frequencies in East Asian samples were 2.5-to-4.9-fold higher than other populations (Figure 5b). This finding caps the credible effect size of NPHP1 duplications in severe diseases,…". What is the size of this duplication? What are the functional implications? Why does this duplication have a significantly high frequency in the East Asian population? Can the authors elaborate a bit more?

Figure 3b: It would be nice if the authors can provide any mechanistic explanation on the trend of SVs distribution along the meta-chromosome.

Figure 5a: Both number 1 and number 3 were marked twice in red and blue. Does this mean that 2q13 and 15q11.2 have both deletions and duplications? Can the authors please clarify this?

Figure 6e: The extremely complex SV involving at least 49
breakpoints across seven chromosomes is interesting. The Circos plot is nice but the authors should clarify in the figure legend what the bold green lines between chr1, chr13 and chr14 indicate.

Referee #2 (Remarks to the Author):

“A global structural variation reference for medical and population genetics” by Collins et al. is a cracking manuscript describing a high-quality large-scale SV resource of over 10,000 whole genome samples constructed from the ExAC/gnomAD resource. It provides comprehensive characterization of these SVs in a timely and exciting piece containing a number of transformative analyses. I especially enjoyed reading the enclosed analyses pertaining to clinical genomics, rare SVs, SV mutational mechanisms which include complex SV classes and SV formation rates. This is in my view a well-written and very important paper, with a high quality of presentation and with an SV resource that will be of great value for the research community (and is likely to be highly used) despite having only SV site-level information. This manuscript seems generally suitable for a wide audience, and presents exciting novelty and insights of interest to a large number of readers. The manuscript is succinctly written, and could essentially be published without too many modifications.

* Since only site-level information with allele frequency (AF) metrics will be available to researchers it is of paramount importance to convince readers of the genotyping quality of this SV resource. The authors performed a number of analyses in this regard, which includes AF comparisons to 1000 Genomes and Mendelian Error Rates. The most accurate analysis for common SVs would be to investigate SV SNP-taggability by nearby single-nucleotide variants, stratified by SV class. Intensity rank sum testing to establish an FDR for deletions and duplications would be likewise very useful.

* Depending on the downstream analysis a mendelian error rate of ~4.1% might be too high and the relatively large number of de novo SVs per child deserves further attention. Did the authors validate any de novo SV using long reads? It would be very helpful if it would be possible to filter the VCF based QUAL or some other QC metric to extract a high-confidence SV set with Mendelian error rate <1%. Would a subset of the data show considerably fewer de novo SVs per child? Are QUAL scores inversely correlated to Mendelian error rates? Mendelian Error Rates should be provided separately by SV type.

* The SV size distribution in Fig. 1f shows an unexpected increase for DUPs and MCNVs at ~5kbp. I suppose this is because of the transition from split read to read depth based genotyping but this has not been discussed in the main text. I was also surprised that there was no peak at 300bp (representing Alu elements deleted from the reference).

* There is a surprising low number of MCNVs in the callset, almost 3-fold lower compared to the number of MCNVs reported in 1000 Genomes phase 3. Given that gnomAD included more samples I rather expected the opposite. Do the authors have an explanation for this? Is this related to previous merging issues in 1000 Genomes?

* Given the small size of the dispersed duplications I assume that the majority of these events has been called using paired ends. What is the average distance between inserted copies and source loci and are these copied loci derived from mobile elements (copy-paste mechanism)?

* Is there any known DNA repair defect in the sample presented in Fig. 6e, with the SV involving at least 49 breakpoints?

Minor comments:

* For a handful of SVs of type INS and CPX the END coordinate is occasionally smaller than POS (SV start). Why?

* The manuscript misses an evaluation of breakpoint accuracy by SV size, class and allele frequency. It would be very helpful if this could be included based on long-read data.

* At present, all the analyses presented in the manuscript are based on a confident subset of 382,610 SVs. This is on its own an impressive number of SV sites compared to prior studies. I don't see a need to inflate that number in the abstract by adding (low-confident) BND variants, or variants with the filter type FAIL.

Referee #3 (Remarks to the Author):

Ample data indicate that structural varation of the genome (SV) is an important source of phenotypic variation in humans, especially as a cause of rare disease. Population-scale databases of exome sequencing data have revolutionized the way human geneticists interpret single nucleotide changes or small indels in coding regions, but similar databases for SV have lagged behind owing to the poor power of exome sequencing to detect SV and resolve their breakpoints. In this manuscript, Collins, Brand, et al. introduce a SV database derived from whole genome sequencing of 14,891 individuals compiled under the auspices of the gnomAD Consortium. This new resource is a welcome addition to the human genetics toolkit and is likely to accelerate the identification of pathogenic variation in a clinical context, as well as broaden our understanding of SV biology. The documentation of the computational methods is exemplary, including full code availability empowering others to reuse the pipeline. The analyses presented and corresponding conclusions are, in general, measured. I will limit my comments to the following objectives : 1) to improve the clarity and accuracy of claims, 2) to improve the usefulness of the data and related analyses.

1. While the authors have done a nice job of contextualizing gnomAD-SV by comparison with other well known SV callsets, analysis of published case-control data, etc. I am surprised by the lack of comparison with the ExAC CNV map and lack of integration with the gnomAD SV/indel callset. The authors spend quite a bit of time characterizing pLoF mutations in this manuscript, and have specifically described the number of genes with homozygous pLoF due to SV (this can also be gleaned from the VCF annotation). One integrative analysis that would be very good to see is a tabulation of the genes that are biallelic pLoF due to compound heterozygosity of one pLoF SNV and on pLoF SV. It would be most helpful for this to be included as a supplementary table. The authors are the only ones that can perform this analysis, as the individual-level genotype data will not be released to the public.

2. The section on “Relevance to disease association and clinical genetics” is most important as it addresses the potential for gnomAD-SV to enhance human genetic of disease across the world. However, I had the most trouble with the clarity of the writing and conclusions in this section. First, the authors present case-control analyses of 4 disease cohorts, with various levels of filtering on gnomAD-SV a priori. This doesn’t seem like a great idea, and certainly not something done as casually as presented here, as differences in the geographic ancestry among the cases, the controls, and the gnomAD samples could easily produce spurious associations. It’s probably important to point out that the quantitative filtering performance reported in these analyses (i.e. what’s in Extended Data Figure 6) is highly dependent on the platform being used for the

case/control (tumor/normal) data - results will differ with WGS data.

4. Next, the authors dive into a detailed analysis of pathogenic allele frequencies at 51 genomic disorder loci. The estimated genotyping error rate for the entire gnomAD-SV resource is in the range of 4-10%. I expect there is some heterogeneity in this rate depending sequence context, SV type, etc. Given the detailed analysis and interpretation provided for these 51 sites, it would seem useful to have a better sense of the quality of genotyping specifically at these loci. Have the authors carefully inspected the genotyping accuracy for all 51 of these GD regions, e.g. by assessing the raw data underlying these calls?

5. The authors state that the observed NPHP1 duplication frequency in EAS "caps the credible effect size of NPHP1 duplications in severe diseases, and underscores the value of characterizing putatively disease-associated SVs across diverse populations." This statement is facile and needs further explanation. Have they excluded genotyping error as an explanation for the high frequency NPHP1 duplications in EAS? How do the authors know this (these) NPHP1 allele(s) in EAS is (are) equivalent to those in other populations? What is the cap on the credible effect sizes of NPHP1 duplications dictated by this observation? Have the authors ruled out alternate explanations for this observation that accommodate existing estimates of the effect size of NPHP1 duplications?

Minor comments:

Supp Fig 12- Would have liked to see more integration with SNV - how many sites in gnomAD appear to be het loF on the basis of SNV data but are actually compound-het LoF when integrated with SV data. Would be even better to annotate this somehow in gnomAD browser.

Supplemental information, pg 23 "Due to the availability of GRCh37-aligned WGS BAM files …" please reword this sentence. Were the BAMS analyzed by Karczewski et al 2019 aligned to a different reference? I would find that surprising since the gnomAD website states "All data are based on GRCh37/hg19."

Line 140 - it would be useful to mention the sequencing depth of gnomAD samples for comparison with 1KG and GTEx.

Line 210- here is another reason that this mutation rate estimate is likely to be biased: the Watterson estimator is based on the assumption of a neutrally evolving population (i.e. the standard neutral Wright-Fisher model), which clearly does not obtain for SVs observed in a mixture of chromosomes from the diverse populations in gnomAD.

Line 315- "These data estimate that roughly 0.05%…" please rephrase; the humans are doing the estimation here.

Line 340 - "filtering all SVs found in an individual genome versus gnomAD-SV dramatically reduced the number of singleton SVs in that genome to a median of 13". Can the authors please clarify in the text the samples being used here? Are these results based on a "leave one out" type of analysis, where one gnomAD sample is removed from the cohort, SV AFs are re-estimated, and filtering is applied to this one sample? If so, this is probably not a realistic example of how gnomAD-SV will be used. What is more likely is that the clinical case genome will be processed with a different SV calling method, and the false positive rate in the resulting callset will be higher, as the analysis will not benefit from the rigorous QC performed here, with >14K samples of background for setting baselines. Does gnomAD-SV provide an advantage in filtering benign CNVs that overlap protein-coding exons, compared to the existing ExAC CNV map, which is based on a large sample set?

Figure 4a - it's probably incorrect to state that the predicted effect of whole-gene inversion is "No

effect”, especially given that the singleton proportion for that class could indicate that they are more deletions than pLoF deletions. Possible effects of a whole gene inversion include disruption of cis- and trans-regulation of the gene, leading to ectopic expression or abnormal expression levels across the normal expression program.

Figure 4d - I noticed this reference to Supplementary Figure 10 is incorrect; it should be Supp Fig 11. I haven’t systematically checked the accuracy of the other figure references.

Figure 5 - panel(C) what do the horizontal dashed lines represent? . panel (D) The authors state that they have “re-estimated ORs for each fo the 51 GDs by comparing to the 29,085 DD cases from (c)”. First I don’t think this statement is coherent, as there appear to be no DD cases explicitly shown in (c) or cited in the caption for (c). Second, the authors should clarify the annotation on the right side -the GD loci within the “0.05%” have a cumulative carrier frequency of 0.05%, I believe, but that is not at all obvious from the diagram or caption.

Supplement, pg 44 - section “Estimating SV mutation rates” - the mathematical symbols didn’t render properly in the PDF, for instance, one line says: “Where was the number of SV sites observed per population for a given SV class and was the total number of chromosomes analyzed in each population”. Clearly “K” and “n” are missing here.

Data sharing:
The gnomAD-SV downloads (the VCF and bed files) available from the gnomAD website could really benefit from a README.

Could the authors provide the revised GD OR estimates shown in 5D as a supplemental table?

Author Rebuttals to Initial Comments:

RESPONSE TO REFEREES

An open resource of structural variation for medical and population genetics

Collins*, Brand*, et al.

Responses to Referee #1

This paper, entitled "A global structural variation reference for medical and population genetics" by Collins et al. presented gnomAD-SV, a supplemental addition to gnomAD that includes SV variations of ~15,000 samples from different populations based on deep WGS sequencing. The authors demonstrated that gnomAD-SV correlated with SV calls from the 1000 Genomes Project (1KG) and also covered additional novel SVs. The authors further reaffirm that many of their inversion signatures correspond to complex SV events, rather than represent simple inversions, as others have shown previously.

A few concerns arise as I read through this manuscript. First, the null distribution simulation of SVs, which the authors used to evaluate SV selective pressure, is, by the authors' own admission, imperfect. However, they don't give any indication about what other factors might be missing, or whether the model in its current state is of sufficient quality for the clinical applications they clearly are aiming for. I believe the authors need to demonstrate that this current model is of sufficient quality, or at least explain what this model needs for improvement, and why it is difficult to achieve.

The referee alludes to several important points related to the evaluation of selection on SVs. We have addressed these points through a new addition to our manuscript, a statistical model for estimating selection on SVs, which we describe in detail in the main manuscript and supplement, but also briefly explain below.

Quantifying selection on SVs presents challenges that are unique from those of coding SNVs/indels. These include the relative sparseness of SVs and the consequent lack of established SV mutation rate models. While gnomAD-SV represents a ~7-fold increase in sample size over the 1000 Genomes Project (Sudmant *et al., Nature*, 2015), our SV data are still sparse and markedly underpowered for locus- or gene-level inference of selection and estimation of fine-scale mutation rates. For these reasons, we did not make any specific assumptions or perform any simulations of SV null distributions in our initial submission. From our experience, simulation-based approaches are heavily confounded by SV class, rearrangement size, genomic context, and technical covariates. Instead, in our initial submission, we relied upon an established, empirical approach to quantify selection via inference from site-frequency spectra: namely, the proportion of singleton variants. As we noted in the initial submission, while the approach performed as expected, it was imperfect as it is sensitive to SV size (as shown in Figure 1h).To address these inherent challenges for our revised submission, we present an improved method for quantifying selection, which we refer to as the Adjusted Proportion of Singletons (APS). This APS metric controls for known covariates that influence singleton proportion for SVs (variant

class, size, context, and evidence), and the baseline reference point is the subset of strictly intergenic SVs, which provides a closer estimate of near-neutral selection. We provide details on the construction and fitting of this APS model in the Supplementary Methods and Supplementary Figure 14, and demonstrate that it is well-calibrated for all SV classes and sizes in Extended Data Figure 5b.

We use this new APS metric to uniformly quantify selection against various groups of SVs in our revised manuscript. While all of the inferences drawn from these analyses remain unchanged from the initial submission, we believe this is a more interpretable and robust approach than our initial presentation. In addition to our benchmarking the APS model and assessment of coding effects from SVs, we also now provide an analysis of selection on CNVs overlapping noncoding elements using APS. These analyses revealed evidence for widespread, but modest, selection against both copy gain and loss of noncoding regulatory elements, although no noncoding effect equaled the strength of selection against protein-coding pLoF SVs. In this same analysis, we found that intragenic SVs overlapping no annotated elements did not exhibit evidence of increased selection, again reinforcing that this APS method is reasonably well-calibrated. Nonetheless, we continue to note that these datasets are sparse and that these models will remain imperfect until the continued aggregation of larger WGS sample sizes and emerging technologies like long-read sequencing allow us to better parameterize true SV distributions and mutation rates.

Second, there is a recapitulation of known disease-associated SVs in the paper, but no demonstration of gnomAD-SV's usefulness at identifying novel disease-associated SVs. An analysis of SVs predicted in gnomAD-SV, but not elsewhere, accompanied with an experimental validation, would be most helpful here.

We share this interest in identifying novel disease-associated SVs, and this is a major focus for the field more broadly. However, given that the overall gnomAD project is meant to be a population-resource, limited phenotype information is available and disease association analyses is not permitted under the data use agreement. We do note that the methods applied here were developed as a complex ensemble of algorithms to improve sensitivity over existing methods, but more importantly to prioritize specificity and precise genotyping for the discovery of *de novo* SVs in family-based association studies of autism (described in detail in Werling *et al.*, *Nat Genet*, 2018). Unfortunately, much of the text describing these methods was detailed in the Supplement rather than the main manuscript, but there were two products of the methods development efforts that directly inform these questions and distinguish the quality control in gnomAD-SV from many prior SV studies:

1. Our rates of *de novo* SVs were derived from almost 1,000 trios rather than a small handful of families, which serve as an excellent proxy for our FDR as a consequence of the combination of false-positive SV discovery in the probands and false-negative genotyping in the parents; and
2. In the Werling *et al.* paper, we described an extensive series of molecular validation studies to determine true positive rates for a curated set of *de novo* variants predicted

from 519 quartet families re-analyzed with the gnomAD pipeline and used for benchmarking in gnomAD-SV here. These confirmation studies involved a series of molecular validation assays from all high-quality *de novo* SV predictions (n=171), including targeted amplification and sequencing, droplet digital PCR (ddPCR) for repeat-mediated SVs, long-insert WGS, chromosomal microarray (CMA), and Sanger sequencing.

In sum, these studies established a 97% unbiased molecular validation rate for credible *de novo* SV predictions generated by highly similar methods to those used in gnomAD-SV. While additional molecular validation would be ideal, as suggested by the reviewer ("SVs predicted in gnomAD-SV, but not elsewhere, accompanied with an experimental validation,"), we note that 86% of all 335,470 high-quality, resolved SVs documented in gnomAD-SV are unique as compared to the existing gold-standard resource (as shown in Supplementary Figure 8), so validation is infeasible for a greater subset of the variants observed, even if we restrict to specific subsets of variants (*e.g.*, those disrupting dominant acting disease genes or high constrained genes). However, we have supplemented this study with a total of seven benchmarking approaches, including a more extensive comparison to long-read raw WGS data for close to 20,000 SVs (see response and Table below)

Regarding new variant discovery, we detail in Figure 2 the discovery of numerous classes of complex SVs that were cryptic to prior technologies, as well as an example of a chromothripsis-like rearrangement in one individual (Figure 6e & Extended Data Figure 8). In the autism quartets used for benchmarking here, we also described discoveries of clearly pathogenic SVs, such as a *de novo* balanced translocation that disrupted *GRIN2B* and a *de novo* cryptic deletion of four exons of *CHD2* (Werling *et al., Nat. Genet.*, 2018). We and others are engaged in large-scale disease association studies of SVs from WGS, and this resource has already been invaluable to interpret and filter such variants for individual cases, even though novel disease gene discovery was not possible in this reference resource.

Third, the authors release their data as hg19 only. Given the age of the hg19 reference, and the fact that recent and future human genome projects will undoubtedly use more recent reference builds, I think the utility of gnomAD-SV will be increased if the authors also release an hg38 version. Other publications have provided data generated against both reference builds to offer broad applicability of their data.

We thank the reviewer for this useful suggestion. All samples analyzed in this study were aligned to the hg19 reference genome as part of their initial data processing. While we could not realign these samples to a second reference genome, the next iteration of gnomAD will be generated from samples native to the hg38 reference genome. However, to increase the utility of the existing gnomAD-SV resource, we have worked with the dbVar team at the NIH National Center for Biotechnology Information (NCBI) to produce and maintain a version of the gnomAD-SV callset lifted over to hg38 coordinates. We are thankful to Tim Hefferon and his team at NCBI, which now provides readily available hg38 coordinates for our dataset using accession nstd166 (https://www.ncbi.nlm.nih.gov/dbvar/studies/nstd166/). We have referenced this accession number both in the main text and in the supplementary methods.

Fourth, a major issue is the lack of direct experimental validation of their results, especially for complex variants and variants found by a single technology. Any new variants identified without providing any direct evidence for these SVs seriously undermines confidence in these novel SVs. Instead, they rely on an analysis of Mendelian concordance, and a very limited set of PacBio sequencing data (4 samples; 0.03% of the sample set). Also, it would be helpful if the authors provide detailed results in the tables/figures for their purported long-read support for up to 88.1% of SVs predicted from short-read WGS.

We concur that careful benchmarking is essential for large-scale reference resources to ensure that results are well calibrated, and benchmarking in general has been a challenge for most SV studies. From the outset, our goal was to provide a comprehensive benchmarking effort for the field, and in this regard we think gnomAD-SV largely achieves this objective. Given the larger sample size and global diversity of gnomAD-SV compared with other published WGS-derived SV datasets, most SVs in gnomAD-SV are unsurprisingly novel by comparison to existing data because most variants in the human population are rare. While this scale of validation is not feasible, we have provided seven measures of SV quality, which taken together provide a composite estimate of sensitivity and specificity for this study that are largely consistent across approaches. Notably, as described above, we have tuned our methods toward precision over sensitivity, so we expect the dominant error mode of the gnomAD-SV callset to be false negatives rather than false positives. These seven approaches are fully detailed in Extended Data Figures 2-3, Supplementary Figures 6-12, Supplementary Tables 4-5, and Supplementary Note 1, and we provide Supplementary Table 4 below as a summary. In addition to the table, we note the following four points:

a. **PacBio long-read WGS comparisons**: We have now performed extensive SV confirmation using existing PacBio long-read WGS and algorithms that directly interrogate raw sequence data, finding a 94% confirmation rate for 19,316 SV observations. While these comparisons involve only four WGS samples that overlap between gnomAD-SV and existing PacBio resources, these four samples represent over one-quarter of the entire sample size of the largest published analysis of PacBio WGS to date: earlier this year, Audano et al. published on 15 PacBio genomes in *Cell*. Likewise, the largest prior direct integration of Illumina short-read WGS and PacBio long-read WGS included three trios from the HGSVC study (Chaisson *et al., Nat. Comms.,* 2019). From these four samples, we were able to assess 19,316 variant observations, yielding a 94% confirmation rate, as mentioned above. To further contextualize these analyses, we have substantially expanded the details of these PacBio long-read comparisons in our revised manuscript. We now provide an entire Extended Data Figure dedicated to PacBio comparisons, where we include breakdowns by sample, SV class, size, and allele frequency. We also provide a new estimate of breakpoint accuracy from PacBio data (Supplementary Figure 10), and a breakdown by variant quality score (Supplementary Figure 12c-d). These analyses have provided significant additional insights into the properties of SV detection from short-read

WGS. We thank the reviewer for this suggestion, and are confident these inclusions have strengthened our revised manuscript.

b. **Trio analyses**: Mendelian transmission analysis provides a relatively comprehensive assessment of variant discovery performance, as it reflects both site discovery and genotyping, and simultaneously considers both false negatives and false positives. In this study, we analyzed 970 complete parent-child trios to provide a multifaceted assessment of error rates. As mentioned in our revision cover letter, we have also completely rerun our methods to regenerate a revised callset targeting a lower FDR based on suggestions from multiple referees regarding family-based FDR, which performed as expected and lowered the apparent *de novo* rate to 3.0% (compared to 4.1% in our initial submission). Furthermore, we now empirically demonstrate that our conclusions in the initial submission are robust to more or less stringent callset filtering (Supplementary Figure 24).

c. **Curation of variants**: Short of molecular validation, the gold standard for *in silico* variant benchmarking and quality control is direct inspection of variant calls. In our revised callset, we have now performed manual review and curation of >15,000 SVs from across the spectrum of SV classes, sizes, frequencies, and contexts, as detailed in the Supplementary Methods (pages 58-61). For example, we manually reviewed read-depth evidence for all 697 autosomal CNVs ≥500kb, finding that virtually all such CNVs (694/697; 99.6%) had unambiguous support in read-depth data. We also performed a similar analysis for all 249 complex SVs with at least one large (≥50kb) predicted CNV interval (n=326 total CNV intervals evaluated), finding just one predicted CNV interval (0.3%; 1/326) lacking unambiguous read-depth support. Examples of these normalized read depth profiles are provided in Supplementary Figure 20. This manual review confirmed the specificity of our methods for large and complex SVs, which are the variant types most likely to impact many of the analyses presented in the manuscript and be of greatest relevance to the clinical genomics community..

d. **Multiplicity of benchmarking approaches**: As mentioned above, we have now applied a battery of seven different approaches to comprehensively assess the quality of the revised gnomAD-SV callset. We provided several of these analyses in our initial submission, but in our revised manuscript we have expanded benchmarking to provide as much orthogonal data as possible for readers to assess variant quality and approach for future SV studies, specifically including a more in-depth analysis of the PacBio samples mentioned above. These data are provided in complete detail in Extended Data Figures 2-3, Supplementary Figures 6-12, Supplementary Tables 4-5, and Supplementary Note 1. We have also updated the previous Supplementary Table 1 with updated results and reproduce it below as a summary:

Analysis	Details	Samples	SVs	Measurement	Value
**1. Trio analysis**	Rate of Mendelian violations per trio for autosomal SVs with complete trio genotypes at at least one non-reference allele present in the trio	2,910 (970 trios)	8,512 per trio (median)	Mendelian violation rate	3.8%
	Rate of apparently *de novo* heterozygous autosomal SVs in children with complete trio genotypes	2,910 (970 trios)	4,686 per trio (median)	Heterozygous genotype error rate (mix of FDR in children, FNR in parents, and true *de novo* SV)	3.0%
	Fraction of homozygous genotypes in children where at least one parent is reference for autosomal SVs with complete trio genotypes	2,910 (970 trios)	1,227 per trio (median)	Genotype error rate (mix of homozygous FDR in parents & heterozygous FNR in children)	7.5%
	Number of untransmitted homozygous genotypes in parents divided by the sum of transmitted heterozygous genotypes in children and untransmitted homozygous genotypes for autosomal SVs with complete trio genotypes	2,910 (970 trios)	4,624 per trio (median)	Heterozygous genotype FNR	1.9%
**2. CMA comparison**	Fraction of autosomal CNVs >40kb from CMA (Sanders *et al.*, 2015) with <30% coverage by simple repeats, segmental duplications, or somatic hypermutable sites that also have matching CNVs (≥50% coverage) in at least 50% of gnomAD-SV samples	1,893	2,524	Sensitivity (for large CNVs)	97.1%
**3. Hardy-Weinberg equilibrium**	Fraction of autosomal biallelic SVs in HWE	12,653	321,140	HWE rate	85.8%
**4. SV & SNV/indel linkage disequilibrium**	Median maximum genotypic correlation coefficient between common (AF≥1%) SVs with <30% coverage by simple repeats and segmental duplications and all SNVs/indels within ±1Mb from a subset of 5,353 overlapping AFR and EUR samples in this study and Karczewski et al. (2019)	5,353	23,597	Pearson Correlation Coefficient (R^2)	0.85
**5. Doubleton genotype analysis**	Fraction of doubleton (i.e., AC=2) SVs with ≤10% coverage by simple repeats and segmental duplications that also appear in two samples from the same population among all doubleton SVs appearing in any two samples (excluding 129 samples with uncertain population assignments)	12,524	32,044	Fraction of intra-population concordant doubleton SVs	79.0%
**6. Comparisons to 1000 Genomes Project**	Correlation of AFs for biallelic autosomal SVs appearing at AF≥1% in either gnomAD and/or 1000 Genomes Project (Sudmant et al., 2015)	N/A	37,907	R^2	0.72
**7. Long-read WGS comparison**	Fraction of SVs with SR support and <30% coverage by simple repeats and segmental duplications that also have long-read WGS support as computationally evaluated by VaPoR (Zhao *et al.*, 2017)	4	4,829 per sample (mean)	PPV	94.0%

Finally, there seems to be a lack of a defined SV calling pipeline provided by the authors that others in the field can use for calling SVs in similar cohorts. Their own pipeline appears to be unpublished and I do not believe it is currently available as a tool for other researchers.

We appreciate the opportunity to further highlight the details and instructions on the SV discovery pipeline. In the interest of space, we provided most details about the pipeline methods and code availability in the supplementary information ("Computational platform" and "Code availability" text provided below for reference). The pipeline and codebase were both made publicly available at the time of initial submission, and the variants were released in the gnomAD-SV database. The pipeline used in this study was based on a prototype as published in Werling *et al., Nat Genet*., 2018. Given the concern raised, we have now featured this accessibility statement more prominently in the main manuscript, and provide multiple references to the locations of the codebase, which we hope will help future readers with accessibility. Notably, Referee 3 commented favorably on the code base and extensive documentation provided.

Computational platform

Most WGS processing, SV discovery, and downstream analyses for gnomAD-SV was conducted on the FireCloud platform (https://software.broadinstitute.org/firecloud/), recently renamed to "Terra" (https://terra.bio/), which is a secure open platform for collaborative genome analysis developed as part of the NCI Cloud Pilot program (Birger *et al., bioRxiv*, 2017). Where relevant, all workflows and methods used in this study have been made publicly available via the FireCloud Methods Repository (https://portal.firecloud.org/#methods).

Code availability

The overall structure and availability of code used in this study is outlined on the home page of the main gnomAD-SV github repository (https://github.com/talkowski-lab/gnomad-sv-pipeline). The gnomAD-SV discovery pipeline is publicly available via a series of methods configured for the FireCloud/Terra platform (https://portal.firecloud.org/#methods) under the methods namespace "Talkowski-SV". The svtk software package used extensively in the gnomAD-SV discovery pipeline is publicly available via gitHub (https://github.com/talkowski-lab/svtk). Most custom scripts used in the production and/or analysis of the gnomAD-SV dataset are publicly available via gitHub (https://github.com/talkowski-lab/gnomad-sv-pipeline). All code is made available under the MIT License, unless stated otherwise.

In the text, the authors state that the gnomAD-SV resource will be made available without restrictions on reuse, and will be integrated directly into the gnomAD Browser. I wonder whether the authors have an estimated date for when the SV data will be publically available in the browser.

The gnomAD-SV dataset was publicly released in the gnomAD browser website (https://gnomad.broadinstitute.org) and we also released a blog post providing a tutorial for users hoping to explore the SV data in the gnomAD browser; that blog post is available here: https://macarthurlab.org/2019/03/20/structural-variants-in-gnomad/

To access the SV data in the gnomAD browser, users must toggle between variant 'modes' using a button on the top right of the page (screenshot below):

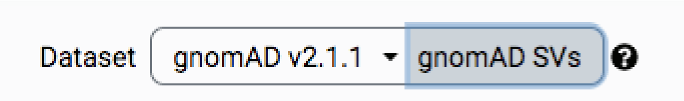

The rate of pathogenic SVs reported in the text didn't match what is reported in the figures. In the Summary (Line 59), Introduction (Line 105) and Results (Line 347) section, the rate was estimated as 0.24. However the rate was reported as 0.4% in the Figure 6C. In addition, how did the authors estimate this rate? I recommend the authors provide some details on the underlying methods.

We regret this confusion regarding the presentation of the data and agree that these statements required greater clarity, which we now provide in this revision. The rate reported in Figure 6c (0.32%) is the proportion of samples carrying a very rare (AF<0.1%) pLoF SV in one of the 57 autosomal genes for which incidental findings are clinically reportable per guidelines from the American College of Medical Genetics (ACMG). The rate reported in the main text (0.18%) is the proportion of individuals carrying a very rare pLoF SV in one of those same 57 genes that would be interpreted as "likely pathogenic" or "pathogenic" per ACMG interpretation criteria following manual review by our team (Kalia *et al., Genet. Med.,* 2017). The difference between these two estimates is comprised of the proportion of samples that carry a very rare pLoF SV in one of the 57 ACMG genes, but which would not meet ACMG criteria to be classified as "likely pathogenic" or "pathogenic" variants. The details of this analysis, including an explanation of interpretation criteria, are now provided in the supplementary methods (page 72). We thank the reviewer for raising this potentially confusing issue for many readers, and the reworked description of this analysis can now be found in the text (lines 379-381) as well as elsewhere in the manuscript (lines 56 and 104).

The description for the pLoF is not consistent in the text. The rare pLoF SVs were reported as "at least 25%" in the Summary (Line 58), ", "approximately 25%" in the Introduction (Line 105) and "up to 25%" in the Results (Line 246). Furthermore, it's not clear to me how the authors came up with this number (25%). Following the main text, I estimated it as 21.7% (=5 rare pLoF SVs/(5 rare pLoF SVs +18 rare pLoF SNV/Indels)). Can the authors please clarify this result and its underlying methods?

This is an important clarification and we have addressed it in our revised manuscript. The difference in the estimate is the functional interpretation of intragenic exonic duplication (IED) SVs, most of which we anticipate result in pLoF, but with a greater dependence on context. Thus, the lower bound of the range of rare pLoF events per genome strictly considers pLoF SVs, and the upper bound is when including both pLoF and IED SVs.

As added context, we have reproduced Supplementary Figure 17 below, which was included in our initial submission and outlines our criteria for annotating genic effects of SVs:

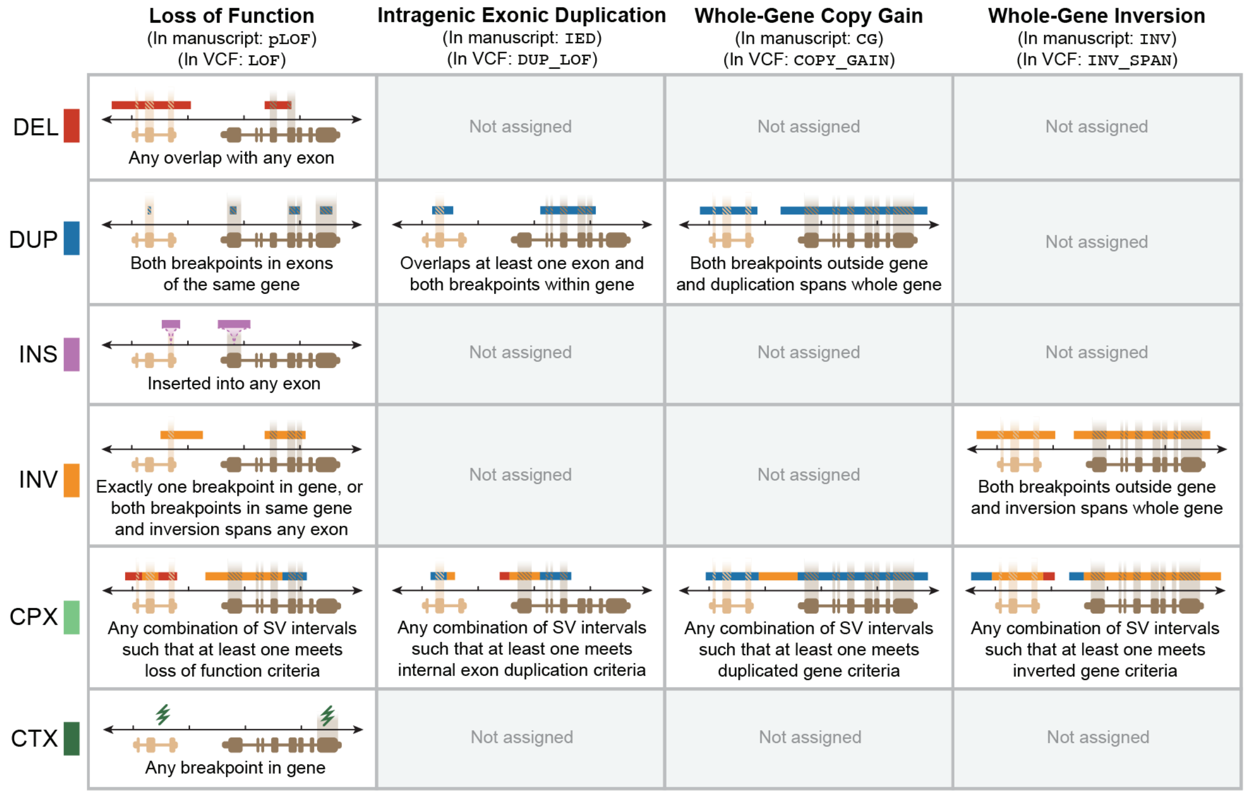

We have clarified our reporting of the fraction of rare protein-truncating events contributed by SVs throughout the manuscript, and paid explicit attention to the wording when presenting these findings. After updating these data to reflect (a) our new SV callset and (b) the revised analyses from the gnomAD SNVs/indels in Karczewski *et al.*, we now uniformly report this statistic as a range of 25-29% in all cases, and have provided more context when first reporting this number in the results section of the manuscript (lines 239-247).

For full clarity, the 25-29% range is derived as follows:

When excluding IED SVs: 25% = 5.5 / 21.8 = (5.5 rare pLoF SVs per genome) / (5.5 rare pLoF SVs per genome + 16.3 rare pLoF SNVs/indels per genome)

When including IED SVs: 29% = 6.8 / 23.1 = (5.5 rare pLoF SVs per genome + 1.3 rare IED SV per genome) / (5.5 rare pLoF SVs per genome + 1.3 rare IED SV per genome + 16.3 rare pLoF SNVs/indels per genome)

Line 115: “the samples across population genetics and complex disease association studies”. Can the authors please provide more details, i.e. the numbers of patient samples and the type of diseases of the patient samples used in this study?

The majority of samples in this study were aggregated from cohorts sequenced by the NIH Centers for Common Disease Genetics (CCDG) and similar WGS collections and thus reflect mostly common and/or complex disease studies. These samples are detailed in the “WGS data aggregation” section of the supplementary methods (supplement page 40).

Over the course of SV discovery, analyses, and public data release, we generated several subsets of the overall cohort; these subsets and steps are described in Supplementary Figure 23. Given the broad data aggregation nature of gnomAD, limited phenotypic metadata is available for most samples, but the counts for each of the largest phenotype groups are listed in the table below.

		**Sample Phenotype (if known)**			
**Data subset**	**Total samples**	Explicitly labeled as "control" or no phenotype specified	Coronary artery disease	Neuropsych. disorder	Autism
VCF including related individuals (strictly used for callset benchmarking)	14,237	8,141	3,023	2,562	511
VCF used for all analyses in manuscript other than Mendelian transmission analysis & microarray benchmarking	12,653	7,260	2,888	2,505	0
VCF appropriately consented for public release	10,847	5,454	2,888	2,505	0

Finally, in our initial submission, we generated a subset of the gnomAD-SV callset to exclude all individuals with known neuropsychiatric disease, and used this subset for the genomic disorder analyses presented given the well-documented associations between neuropsychiatric disorders and a subset of these regions. In our revised submission and in response to the Referee’s suggestion, we now make this subset publicly available, as well as a subset restricted to individuals explicitly labeled as “controls” by their contributing projects. These two new subsets of the gnomAD-SV dataset will be publicly released with the existing gnomAD-SV dataset in the gnomAD Browser, and we hope they will prove useful for end-users.

Line 136: "BNDs substantially inflated the variant count, were enriched in false positives". How do the authors determine whether one BND is false positive or not?

In this and previous studies (*e.g.*, Werling *et al., Nat. Genet.*, 2018), we observed that BNDs were enriched for features and metrics indicative of low-quality variants, including localization to sequences with poor mappability, low genotype quality scores, and reduced rates of Mendelian transmission based on family data. Furthermore, given that BNDs by definition cannot be resolved to an existing, parsimonious alternate allele structure, they are more prone to both false negatives and false positives (otherwise they would be able to be resolved into discrete alternate allele structures).

In gnomAD-SV, even after heavy filtering through our improved methods, we still observe that BNDs present in our final SV callset have higher rates of apparent de novo SVs in 970 parent-child trios across any class of SVs passing all filters (Extended Data Figure 2a). The rate of apparent de novo BNDs (5.6%) is nearly double that of fully resolved CNVs (3.0%) or balanced SVs (3.1%) passing all post hoc filters (see Extended Data Figure 2a, reproduced below). Thus, we have not considered these incompletely resolved variants in our analyses.

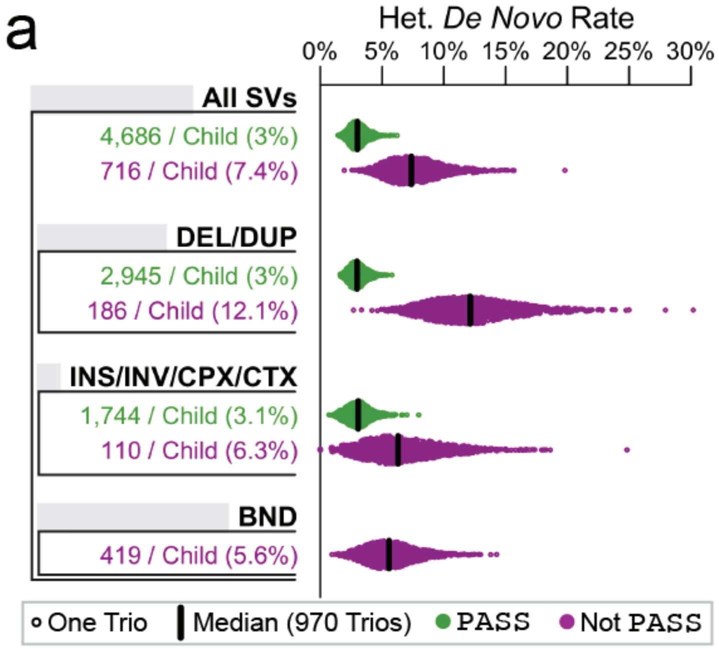

Line 150: "97.8% sensitivity to detect large CNVs (>40kb) previously reported from microarrays in 1,893 individuals (ref 25)". The Ref 25 paper used 10,220 samples from 2,591 ASD families for CNV study. What the criteria the authors used to select those 1,893 samples for the comparison here? Also can the authors provide the exact CNV numbers to calculate 97.8%? Since the Ref 25 was an ASD study, I would like to know whether this current study included any ASD samples and whether those 97.8% CNVs were enriched in the ASD samples. If no ASD samples were included in this study, can the authors explain why 97.8% of ASD CNVs can be detected in non-ASD samples?

As the reviewer notes, the Sanders *et al.* study (Ref 25 in the original submission) considers ASD families; more specifically, simplex ASD quartets, or four-member families with unaffected parents, one affected child, and one unaffected child. All ASD samples and their siblings were excluded from all analyses in this manuscript except for callset quality assessments and benchmarking. The 1,893 samples from the ASD families are a subset of the 10,220 individuals from Sanders *et al.* for which WGS data was generated by the Simons Foundation as described in several recent publications (*e.g.*, Werling *et al., Nat. Genet.*, 2018; Turner *et al., Cell*, 2018; Brandler *et al., Science*, 2018). In total, WGS data was generated on 2,076/10,220 samples from Sanders *et al.*, 1,893 of which passed all sample-level quality control in gnomAD-SV and were retained for benchmarking analyses. As noted above, all ASD samples and their siblings were removed after callset benchmarking but prior to analysis. We have clarified this in the supplementary methods under the section titled "WGS data aggregation".

Regarding the comparison to microarrays: we provided the number of samples (n=1,893), number of CNVs evaluated (n=2,524), and the overall outcome of this analysis in Supplementary Table 4, and provide the methods for this analysis in the supplementary methods under the section titled "Comparison to chromosomal microarray data on matched samples," found on page 62 of the supplementary information. Given that 75% of samples from Ref 25 are not affected with ASD but come from quartet families, effectively all of the genetic variation in the ASD proband is inherited from unaffected parents. Thus, we can use all four members of these families for benchmarking purposes, both by comparison to existing microarray-based CNV calls (the analysis in question) as well as for Mendelian violation rates.

To summarize, there are no ASD-affected individuals from Ref 25 present for analyses presented throughout the study. We have added more detail to the supplementary methods to improve the interpretability of our use of this cohort.

Line 155: The authors compared their results with 1KG and found 87.4% of SVs are novel. I would like to know how many SVs reported in 1KGP can be detected in your results. The precision and recall rate should be provided here.

We thank the referee for this question; indeed, this is an important point and the corresponding results were provided in panels a-c of a supplementary figure from our initial submission (now Supplementary Figure 6 in our revised submission). These analyses found that 57% of the SVs reported by the 1000 Genomes Project were captured by gnomAD-SV. Panels d-f of that same

figure show the reciprocal analysis, where we calculated the fraction of SVs reported by gnomAD-SV also captured in the 1000 Genomes Project (14%).

It is important to note that the samples from the 1000 Genomes Project (~7X coverage WGS data) were not included in the gnomAD-SV cohort, and thus we would not expect to detect the majority of singleton and rare SVs documented in the 1000 Genomes Project. Therefore, assessment of precision/recall is not applicable here; however, the 1000 Genomes Project samples have now been sequenced to 30X coverage, and these analyses will be available in a future release of gnomAD, as well as projects such as the Human Genome Structural Variation Consortium (HGSVC).

Lines 189-190: "Among canonical SVs, deletions were collectively more rare than other classes (P < 1x10-100; one-sided Wilcoxon Test; Supplementary Figure 8)". While in Figure 1C, the authors clearly showed that deletions are the most abundant SV class. Can the authors please address this discrepancy?

In this context, "rare" was being used to refer to the site-frequency spectrum and the lower average allele frequency of deletions compared to other variant classes, not the total count of variants documented per class. We agree this is a potentially confusing point for readers and have removed this line from the revised manuscript, as it is not a major finding and has been demonstrated in previous studies.

Line 206: Why is the mutation rate of 0.35 de novo SVs per generation in this study more than 2-fold the rate of the 519 quartets (~0.15)? Can the authors provide the age distribution of the samples?

The Watterson estimator is a calculation performed on population-level genetic data. As noted in the main text, it is not a direct observation of *de novo* variants. As such, the 95% confidence interval for the SV mutation rate based on gnomAD is 0.13-0.44 SVs per generation; this interval is statistically consistent with the SV mutation rate from molecular validation experiments in the 519 quartets published in Werling *et al.* (0.16 SVs per generation). Nonetheless, the reviewer raises an important point that, while not significantly different than the validated *de novo* SVs from the 519 quartets, there are distinctions between the two studies:

1. The 7-fold larger sample size of gnomAD-SV improved overall sensitivity compared to Werling *et al.*, as is seen in the 27% increase in the average number of SVs detected per genome (5,863 in Werling vs 7,439 in this more stringently filtered revision of gnomAD-SV). We have consistently observed in previous studies that increasing sample size when joint-calling SVs improves power for variant detection, so this increase in variant count was expected.
2. gnomAD-SV includes both mobile element insertions as well as non-transposon small sequence insertions, whereas Werling *et al.* only considered mobile element insertions.

This is observed in the greatest difference between mutation rate estimates from gnomAD-SV and Werling et al. coming from insertion variants (pink point in Figure 3a).

3. As mentioned above, the Watterson estimator is a statistical estimate, whereas the rate reported in Werling *et al.* was based on variants for which molecular assays could confirm both (1) variant validity and (2) inheritance status. Therefore, we expect the rates from Werling et al. to be a conservative lower estimate.

We now provide age distributions for various subsets of the gnomAD-SV cohort in Supplementary Figure 2, and mention the average sample age in the main text on lines 113-114 (mean age = 49 years old).

Line 243: The citation to the Extended Data Figure 2e-h should be corrected as Extended Data Figure 3e-h

Thank you for this careful review. We have corrected the figure reference in our revised manuscript.

Lines 309-311: "…duplications of NPHP1 at 2q13, where carrier frequencies in East Asian samples were 2.5-to-4.9-fold higher than other populations (Figure 5b). This finding caps the credible effect size of NPHP1 duplications in severe diseases,…". What is the size of this duplication? What are the functional implications? Why does this duplication have a significantly high frequency in the East Asian population? Can the authors elaborate a bit more?

Duplications of *NPHP1* have been previously reported among Japanese individuals with autism (Yasuda *et al., Ann. Gen. Psychiatry*, 2014) and speculated as potentially disease-associated based on ascertainment in clinical microarray databases (Dittwald *et al., Genome Res.*, 2013). However, to our knowledge, the disease association of *NPHP1* duplications has not been statistically established or replicated in larger cohorts.

In gnomAD-SV, we identify a duplication present in >100 samples (including several homozygotes) for the reported *NPHP1* gene duplication locus. The carrier frequency of this duplication is significantly higher in East Asian samples compared to other populations. The duplication includes the entire *NPHP1* gene, which presumably results in increased gene dosage of *NPHP1*, but this prediction would require functional studies of variant carrier biospecimens, which are not available to us. We also identify a second duplication present in 3 samples matching the larger (620kb) 2q13 microdeletion syndrome locus, which includes *NPHP1* among other genes. In our revised manuscript, we now provide read depth-based evidence for the predicted CNV carriers for all genomic disorders in Supplementary Figure 20; the relevant panels from this figure are reproduced here for clarity:

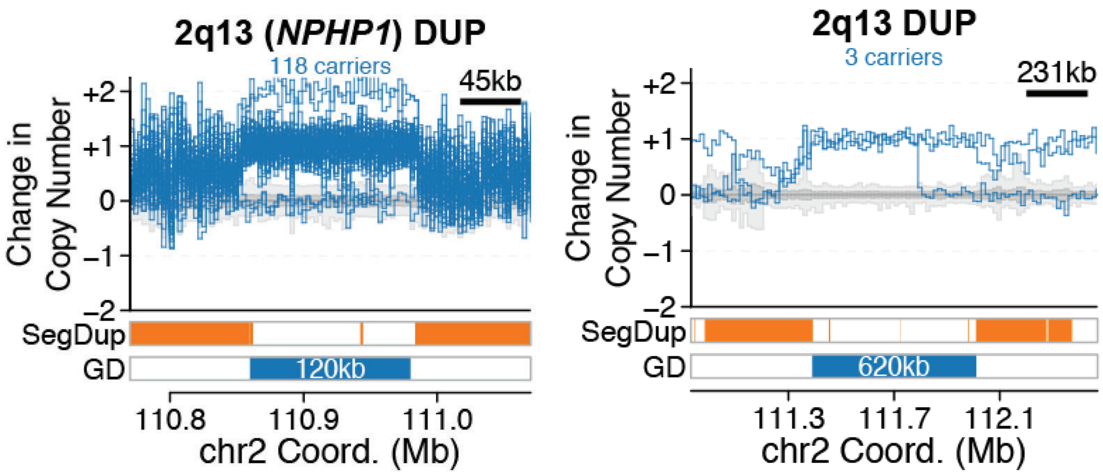

We highlighted these *NPHP1* duplications in our manuscript because (1) it was the only genomic disorder evaluated in our analyses that exhibited striking population stratification and (2) it is a cautionary example where population structure and genetic ancestry may lead to over-interpreting disease risk conferred by CNVs at such loci. However, given that this particular finding was a point of focus for multiple Referees, we have deemphasized it in our revised manuscript for clarity.

Figure 3b: It would be nice if the authors can provide any mechanistic explanation on the trend of SVs distribution along the meta-chromosome.

We thank the reviewer for this suggestion. While the mechanisms that mediate SV formation have been the focus of prior studies (*e.g.*, Monlong *et al., Nucleic Acids Res*., 2018), and the non-uniform distribution of SVs across chromosomal contexts has been noted from microarray studies (Cooper *et al., Nat. Genet.*, 2011) and just recently from long-read WGS analyses of 15 genomes (Audano *et al., Cell*, 2019), we agree that our findings in this study could benefit from additional context. Thus, in our revised submission, we have now expanded these analyses to include a statistical analysis of co-occurrence of SVs with seven classes of repetitive elements (Supplementary Figure 16b-g), and describe the results of these analyses in the main text. In brief, we find a strong correspondence between SV density and numerous repeat classes, consistent with prior research on SV mechanisms of formation.

Figure 5a: Both number 1 and number 3 were marked twice in red and blue. Does this mean that 2q13 and 15q11.2 have both deletions and duplications? Can the authors please clarify this?

Yes, this is the correct interpretation. Some genomic disorder loci are reciprocal, but frequencies of deletion & duplication are not always symmetrical. This has been noted in the corresponding figure legend of our revised submission.

Figure 6e: The extremely complex SV involving at least 49 breakpoints across seven chromosomes is interesting. The Circos plot is nice but the authors should clarify in the figure legend what the bold green lines between chr1, chr13 and chr14 indicate.

We thank the reviewer for this suggestion. We have added clarifying text to the legends of Figure 6 and Extended Data Figure 8 in our revised submission.

Responses to Referee #2

A global structural variation reference for medical and population genetics" by Collins et al. is a cracking manuscript describing a high-quality large-scale SV resource of over 10,000 whole genome samples constructed from the ExAC/gnomAD resource. It provides comprehensive characterization of these SVs in a timely and exciting piece containing a number of transformative analyses. I especially enjoyed reading the enclosed analyses pertaining to clinical genomics, rare SVs, SV mutational mechanisms which include complex SV classes and SV formation rates. This is in my view a well-written and very important paper, with a high quality of presentation and with an SV resource that will be of great value for the research community (and is likely to be highly used) despite having only SV site-level information. This manuscript seems generally suitable for a wide audience, and presents exciting novelty and insights of interest to a large number of readers. The manuscript is succinctly written, and could essentially be published without too many modifications.

We thank the referee for their assessment of our work and its potential value to the biomedical research community in the coming years.

** Since only site-level information with allele frequency (AF) metrics will be available to researchers it is of paramount importance to convince readers of the genotyping quality of this SV resource. The authors performed a number of analyses in this regard, which includes AF comparisons to 1000 Genomes and Mendelian Error Rates. The most accurate analysis for common SVs would be to investigate SV SNP-taggability by nearby single-nucleotide variants, stratified by SV class. Intensity rank sum testing to establish an FDR for deletions and duplications would be likewise very useful.*

This is a great suggestion, and we agree that the inclusion of such analyses as well as other genotyping quality assessments are paramount to the value of the resource. We describe some of the additions and QC metrics in the analyses in response to Referee #1 above (see response to point #4), and provide further detail below.

In our revised manuscript, we now present an analysis of linkage disequilibrium (LD) between SVs and SNVs/indels. We find that the average common (AF>1%) SV is well-tagged by nearby SNVs/indels (median peak R^2=0.85), after excluding variants in repetitive sequence contexts where both biological factors (*e.g.*, complex or multiallelic haplotype structures) and technical factors (*e.g.*, reduced genotyping accuracy for both SNVs and SVs) confound accurate LD calculations. Patterns of LD were largely consistent across SV classes, sizes, and frequencies, and correlated positively with variant quality scores. We now describe these data in the main manuscript on lines 148 and 341-342, and in Extended Data Figure 2c and Supplementary Figures 7 & 12.

Intensity rank sum testing of B-allele frequencies or normalized read depths is another valid approach for estimating FDR of CNVs. However, since our SV discovery pipeline uses both SNV B-allele frequencies and read depth from WGS data to filter and classify SVs, reusing these same data to estimate FDR would not be an independent or orthogonal measurement, and we anticipate

it would likely be anti-conservative and underestimate our true FDR. Instead of intensity rank sum testing, we have greatly expanded the PacBio long-read WGS comparisons since our initial submission, as described in response to Referee #1, which now includes an estimation of breakpoint accuracy. As another new approach to evaluate genotyping accuracy, we considered doubleton SVs, finding that they overwhelmingly appeared isolated to specific populations, as expected (79.0% of doubletons were intra-population vs. 35.0% expected by chance; $P<10^{-100}$, one-sided binomial test). We now provide extensive extended data and supplementary materials to permit readers to better assess the quality of the gnomAD-SV dataset: see Extended Data Figures 2-3, Supplementary Figures 6-12, Supplementary Tables 4-5, and Supplementary Note 1 for more information.

** Depending on the downstream analysis a Mendelian error rate of ~4.1% might be too high and the relatively large number of de novo SVs per child deserves further attention. Did the authors validate any de novo SV using long reads? It would be very helpful if it would be possible to filter the VCF based QUAL or some other QC metric to extract a high-confidence SV set with Mendelian error rate <1%. Would a subset of the data show considerably fewer de novo SVs per child? Are QUAL scores inversely correlated to Mendelian error rates? Mendelian Error Rates should be provided separately by SV type.*

We fully agree that, while a 4.1% Mendelian violation rate (MVR) is fairly stringent among population-based SV studies to date, it may be undesirably high for certain applications, such as the precise identification of *de novo* SVs in family-based studies, or for optimal precision of allele frequency estimates for rare variants. Indeed, in our previous analysis of 519 autism quartets using a prototype of the gnomAD-SV pipeline, we demonstrated that strict filtering and variant curation can yield an MVR < 1% where 97% of all predicted *de novo* SVs were successfully validated by molecular assays, albeit at the expense of sensitivity (Werling *et al., Nat. Genet.*, 2018). For gnomAD-SV, we initially targeted a <5% MVR based on two main considerations:

1. The primary purpose of gnomAD-SV is as a population-scale reference catalogue of SVs where sensitivity is especially valuable and overly strict filtering would be disadvantageous; and
2. To stay consistent with the ~5% FDR precedent set by previous landmark public SV resources (*e.g.*, by the 1000 Genomes Project; Sudmant *et al., Nature*, 2015).

Nevertheless, one of the main focuses of our revision process based upon these comments was to improve the MVR in gnomAD-SV, and to provide additional context such that users of gnomAD-SV can further filter the data to suit their needs. The steps we have taken to address these points are described below.

First, we completely regenerated the SV callset on the entire gnomAD-SV cohort with augmented methods and parameters tuned to reduce our MVR. Specifically, we further refined our hierarchical minimum genotype filtering procedure (described in Supplementary Methods) to uniformly increase stringency across SV classes, sizes, frequencies, WGS evidence types, and genomic contexts. As we mentioned above, this process successfully reduced our observed *de novo* rate by nearly one-third (down to 3.0% in the revised callset from 4.1% in the initial submission; see Extended Data Figure 2a). This filtering has undoubtedly improved the overall specificity of the

dataset, though we note from Extended Data Figure 2a provided above the reduction in sensitivity (represented in the reduced number of variants per individual from 8,202 to 7,439 in the current revision), as the vast majority of filtered variants in the trio analyses are still observed as inherited variants in the parents. Nonetheless, given this balance of sensitivity and specificity we consider this a significant improvement in the resource, which is still more sensitive than all contemporary population SV studies, and the improved specificity does not alter any of the conclusions of our analyses (see Supplementary Figure 24).

Second, as described above (in particular, in response to Referee #1 point #4, and Referee #2 point #1), we have performed extensive benchmarking of our revised callset, including numerous breakdowns by SV class, size, frequency, and genomic context. These analyses are described in Extended Data Figures 2-3, Supplementary Figures 6-12, Supplementary Tables 4-5, and Supplementary Note 1.

Third, prompted by Referee #2's excellent suggestion, we have performed a comprehensive cross-assessment of three orthogonal quality assessment strategies versus variant quality (QUAL) scores, stratified by SV class. Specifically, we considered PacBio long-read WGS confirmation rate, LD between SVs and nearby SNVs/indels, and *de novo* rate from 970 trios. All three analyses demonstrated that the QUAL scores provided in the gnomAD-SV dataset are well-calibrated across SV classes and inversely correlated with error rates, and therefore can be used for *post hoc* filtering should users of gnomAD-SV desire a higher-quality subset of the data. These analyses are presented in Supplementary Figures 11-12; we reproduce Supplementary Figure 12 below for convenience:

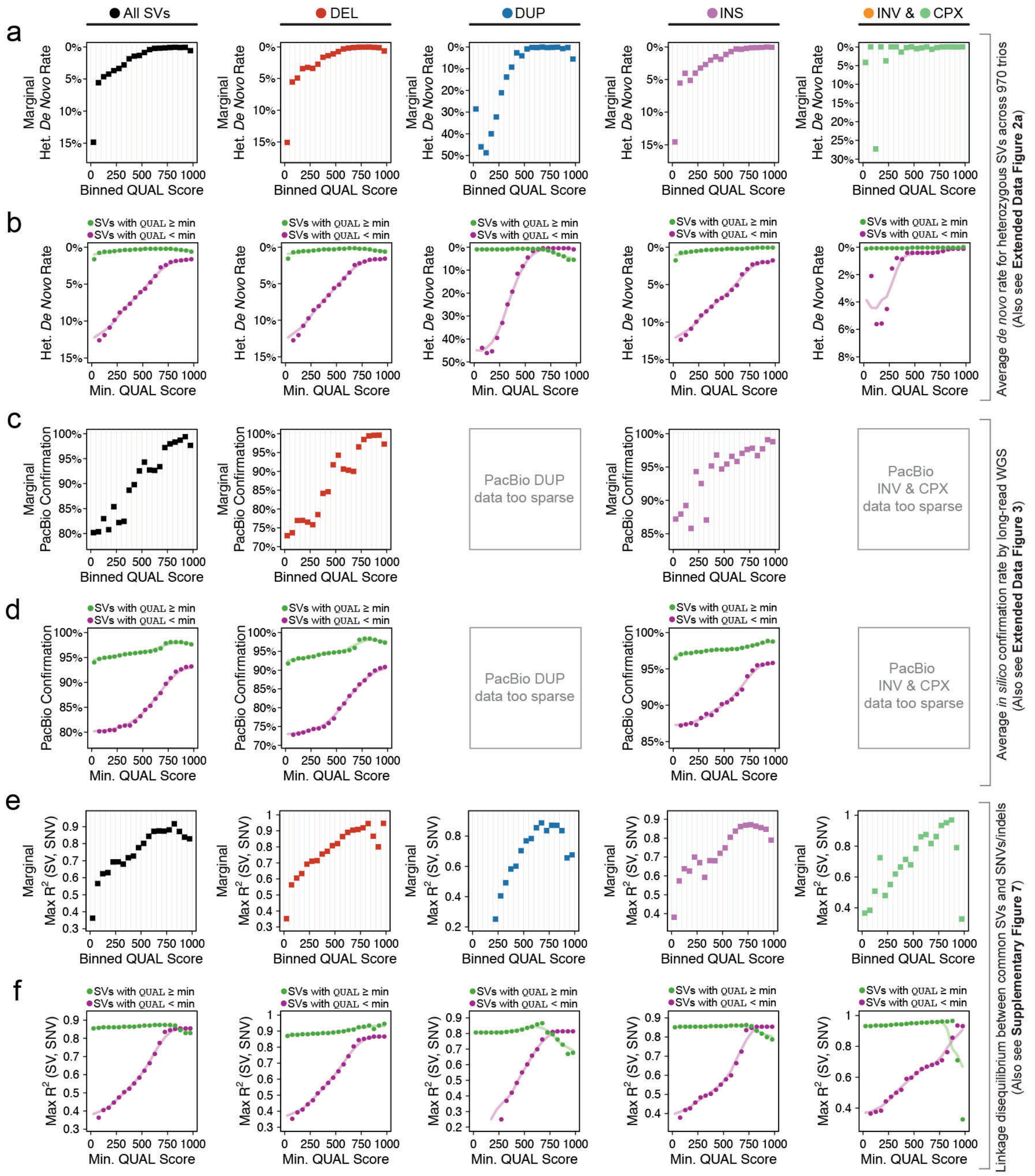

Abbreviated legend: (a-b) de novo rate analysis; (c-d) PacBio comparisons; (e-f) SNV/indel LD analysis.

Finally and most importantly, we reproduced our analysis described in the manuscript at three different variant filtering criteria, and found no material differences in our conclusions based on looser or stricter filtering thresholds. These analyses are presented in detail in Supplementary Figure 24, but are reproduced below for convenience:

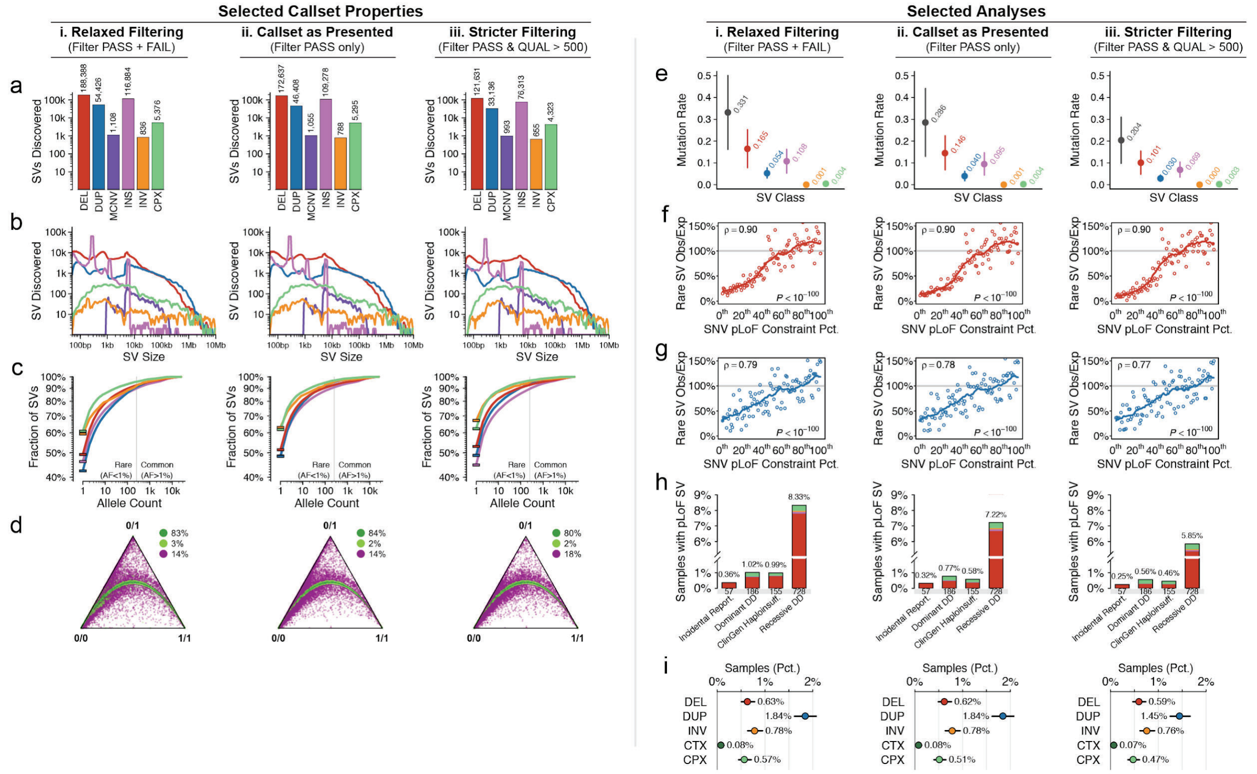

Abbreviated legend: (a) counts of SVs; (b) SV size distributions; (c) allele frequency distributions; (d) Hardy-Weinberg equilibria; (e) Watterson estimator-derived mutation rates; (f) correlations of rare pLoF SVs with pLoF SNV constraint; (g) correlations of rare CG SVs with pLoF SNV constraint; (h) carrier rates for very rare pLoF SVs in medically relevant gene lists; (i) carrier rates for rare, large (≥1Mb) SVs.

In conclusion, we thank the reviewer for raising this point, as it provided an opportunity to more thoroughly benchmark our dataset and improve the overall quality of the gnomAD-SV resource and this manuscript. Based on the lack of difference in any major result or inference due to less or more strict filtering criteria, we conclude that the 3.0% MVR dataset as presented in our revised manuscript is robust to technical filter selection and broadly applicable to many aspects of genetic research and clinical diagnostics.

** The SV size distribution in Fig. 1f shows an unexpected increase for DUPs and MCNVs at ~5kbp. I suppose this is because of the transition from split-read to read depth based genotyping but this has not been discussed in the main text. I was also surprised that there was no peak at 300bp (representing Alu elements deleted from the reference).*

This is an astute assessment that the transition in SV density for DUPs and MCNVs at 5kb is due to the >5kb restriction applied to CNV calls contributed only by read depth-based analyses. This threshold was selected due to our assessment that the false discovery rate is inflated for CNVs

discovered uniquely by read depth-based approaches below 5kb compared to those ≥5kb. We have added extra text to the supplementary methods to make this filtering behavior clearer.

We found that *Alu* deletions were markedly enriched among deletions failing one or more VCF FILTER statuses: just 4.2% (7,285/172,637) of all filter-passing deletions matched annotated SINE repeats compared to 11.9% (1,876/15,751) of deletions that failed one or more VCF FILTERs and were excluded from all analyses in our manuscript. Thus, the lack of a more distinct peak at ~300bp among deletions is likely due to the filtering and technical challenges of genotyping SVs with high confidence in repetitive sequence contexts, especially in this relatively small deletion size range. Indeed, we observe that SVs are universally enriched among one or more repeat element classes, as expected, and we provide new data and analyses to this point as part of Supplementary Figure 16b-g.

** There is a surprising low number of MCNVs in the callset, almost 3-fold lower compared to the number of MCNVs reported in 1000 Genomes phase 3. Given that gnomAD included more samples I rather expected the opposite. Do the authors have an explanation for this? Is this related to previous merging issues in 1000 Genomes?*

As the referee notes, merging high copy-number polymorphisms across many thousands of samples presents challenges for short-read WGS. Therefore, we were intentionally strict on merging and classifying MCNVs, almost certainly at the expense of sensitivity. We required the following filters before classifying a variant as an MCNV:

1. The CNV must have at least four observed copy states;
2. At least 1% of samples must belong to the fourth copy state or greater;
3. The CNV must have read-depth support; and
4. The CNV must be > 5kb in size.

We have no systematic evidence that the MCNVs documented in the 1000 Genomes Project are incorrect. Rather, the sensitivity for MCNVs in gnomAD-SV is probably low, and it is likely that some common duplications in gnomAD-SV may have a handful (<1%) of samples at supernumerary copy states. Conversely, most MCNVs catalogued in gnomAD-SV are likely truly MCNV. We provide distributions of observed copy numbers for all MCNVs in the gnomAD Browser to instill added confidence in our MCNV calls.

** Given the small size of the dispersed duplications I assume that the majority of these events has been called using paired ends. What is the average distance between inserted copies and source loci and are these copied loci derived from mobile elements (copy-paste mechanism)?*

This is the correct inference: paired-end or split-read data is required to map all dispersed duplications. It is possible that some dDUPs are mobile elements, but we classified all candidate dDUPs as MEIs based on overlap of duplicated sequence with annotated repetitive elements.

However, it is possible that some MEIs may have evaded this annotation process, especially if the source repeat element is poorly represented by RepeatMasker.

Among dDUP calls not matching our criteria to be classified as MEIs based on existing annotations, we found that 13.5% (152/1,128) were interchromosomal, while the remainder were intrachromosomal with the following distribution of distances between copied sequence and insertion point:

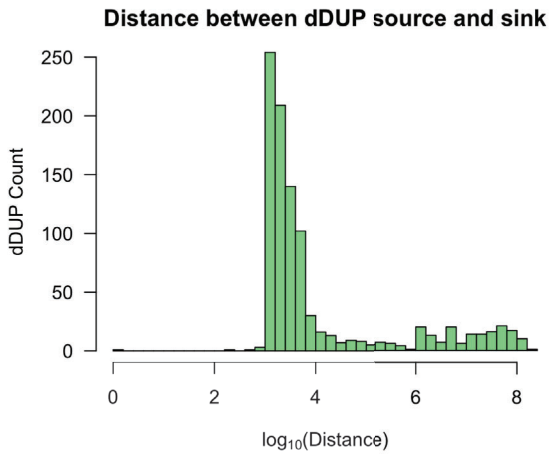

* *Is there any known DNA repair defect in the sample presented in Fig. 6e, with the SV involving at least 49 breakpoints?*

We appreciate the proposal of this intriguing hypothesis, as it is one that we have long been interested in since our first descriptions of chromothripsis and other ultra-complex rearrangements in the germline of developmental disorder cases and controls, but have yet to identify such mechanisms in our prior analyses (*e.g.*, Chiang *et al., Nat. Genet.*, 2012; Redin *et al., Nat. Genet.*, 2017; Collins *et al., Genome Biol.*, 2017).

For context regarding this specific rearrangement, all changes in copy-number appear at whole integer states, suggesting the rearrangement is germline in origin, either inherited or *de novo*. Parental DNA was unavailable for this sample, so we were unable to confirm the mode of inheritance. Available metadata for this sample, while limited, also had no indication of any phenotype that might indicate a DNA repair defect, such as a known germline cancer syndrome.

To investigate this possibility, we compared all high-confidence pLoF SNVs, indels, and SVs in this sample against two gene sets:

1. 317 dominant-acting, high-confidence consensus cancer driver genes (from COSMIC v90 Tier 1; available from https://cancer.sanger.ac.uk/census)
2. 280 genes labeled with the Gene Ontology term for “DNA repair” (GO:0006281), while also requiring experimental evidence for this classification in humans

In total, we identified 228 genes and 158 genes predicted to be inactivated by high-confidence SNVs/indels or SVs in this sample, respectively. Given that most variants per genome are common, and are therefore incompatible with causing a major DNA repair defect, we further restricted to rare (AF<1%) variants. Among the genes inactivated by rare variants, none matched a high-confidence cancer driver gene or annotated DNA repair gene. Thus, we were unable to identify any evidence for a DNA repair defect in this sample, though we continue to explore such hypotheses in our ongoing studies of chromosomal abnormalities in disease cohorts where complex rearrangements are more abundant.

** For a handful of SVs of type INS and CPX the END coordinate is occasionally smaller than POS (SV start). Why?*

We thank the referee for noting this behavior in the gnomAD-SV callset. This was a variant representation issue for events involving inter- or intra-chromosomal insertions, or other translocations of segments of DNA. For clarity, we have reformatted the gnomAD-SV resource files (both VCF and BED) such that CHROM, POS, and END correspond to a single interval on the primary/index chromosome, and have added CHR2, POS2, END2 as supplementary variant annotations where necessary for inter-chromsomal SVs or other insertion-like variants. We hope this modified variant representation will be clearer for individuals using the gnomAD-SV dataset.

** The manuscript misses an evaluation of breakpoint accuracy by SV size, class and allele frequency. It would be very helpful if this could be included based on long-read data.*

We thank the referee for this suggestion. We have now performed an analysis of SV breakpoint accuracy stratified by SV size, class, and frequency based on available PacBio long-read WGS datasets. When considering the PacBio long-read data as ground truth (which will not always be the case, such as assembly errors in regions with low sequence complexity), we found that 59.8% of SV breakpoint coordinates in gnomAD with direct read evidence (~93% of all SVs) are accurate within a single nucleotide, and 75.9% of breakpoint coordinates are accurate within ±10bp. These new analyses are quite useful for evaluation of breakpoint precision, and are presented in the revised manuscript at lines 150-154 of the main text as well as Supplementary Figure 10.

** At present, all the analyses presented in the manuscript are based on a confident subset of 382,610 SVs. This is on its own an impressive number of SV sites compared to prior studies. I don't see a need to inflate that number in the abstract by adding (low-confident) BND variants, or variants with the filter type FAIL.*

We share the referee's sentiment that the most useful information is gleaned from fully resolved, high-quality SVs; indeed, this is precisely why we restrict all analyses as presented in the study to fully resolved, high-quality variants, and inadequate variant filtering is a critical shortcoming of many WGS-based SV studies. However, as one major use for datasets like gnomAD is as a

population-level variant screening resource (where sensitivity in the reference dataset is especially valuable), we decided to retain the lower-confidence and/or unresolved variants like BNDs or filter FAIL SVs, as they may serve a purpose for a subset of gnomAD users that would like to compare against existing callsets (*e.g.*, from large-scale consortia studies using independent methods).

As mentioned above, based on Mendelian transmission analyses, we note that the variants excluded from the principal analyses presented in the study due to being unresolved and/or filter FAIL still exhibit >90% Mendelian inheritance (*e.g.*, Extended Data Figure 2a) and—while being clearly lower-quality than filter PASS variants—still appear to contain a substantial majority of likely valid SVs (*e.g.*, *Alu* deletions, as mentioned earlier).

Responses to Referee #3

Ample data indicate that structural variation of the genome (SV) is an important source of phenotypic variation in humans, especially as a cause of rare disease. Population-scale databases of exome sequencing data have revolutionized the way human geneticists interpret single nucleotide changes or small indels in coding regions, but similar databases for SV have lagged behind owing to the poor power of exome sequencing to detect SV and resolve their breakpoints. In this manuscript, Collins, Brand, et al. introduce a SV database derived from whole genome sequencing of 14,891 individuals compiled under the auspices of the gnomAD Consortium. This new resource is a welcome addition to the human genetics toolkit and is likely to accelerate the identification of pathogenic variation in a clinical context, as well as broaden our understanding of SV biology. The documentation of the computational methods is exemplary, including full code availability empowering others to reuse the pipeline. The analyses presented and corresponding conclusions are, in general, measured. I will limit my comments to the following objectives : 1) to improve the clarity and accuracy of claims, 2) to improve the usefulness of the data and related analyses.

We thank the referee for their kind remarks, and particularly for their acknowledgement of our commitment to the transparent release of all data and code used in this study.

1. While the authors have done a nice job of contextualizing gnomAD-SV by comparison with other well-known SV callsets, analysis of published case-control data, etc. I am surprised by the lack of comparison with the ExAC CNV map and lack of integration with the gnomAD SV/indel callset. The authors spend quite a bit of time characterizing pLoF mutations in this manuscript, and have specifically described the number of genes with homozygous pLoF due to SV (this can also be gleaned from the VCF annotation). One integrative analysis that would be very good to see is a tabulation of the genes that are biallelic pLoF due to compound heterozygosity of one pLoF SNV and on pLoF SV. It would be most helpful for this to be included as a supplementary table. The authors are the only ones that can perform this analysis, as the individual-level genotype data will not be released to the public.

We agree with this referee's interest in a direct comparison between the ExAC CNV map and the gnomAD-SV dataset. Unfortunately, given the lack of overlapping samples between the exome sequencing data used for the ExAC CNV map and the WGS samples in gnomAD-SV, we were unable to directly compare the two CNV callsets.

In lieu of directly comparing overlapping samples, we also considered including a site-level comparison between ExAC and gnomAD-SV. However, as CNV discovery from exome sequencing is vastly more technically challenging than CNV discovery from WGS, we note that there are limitations to the ExAC CNV map that make a direct comparison to the gnomAD-SV fraught, including:

1. The ExAC CNV calls were restricted to rare (frequency < 0.5%) CNVs of protein-coding exons, while gnomAD-SV includes SVs of all frequencies and sequence contexts;
2. The average CNV size in ExAC was 73kb, compared to a median size of 0.9kb for CNVs in gnomAD-SV;

3. By definition, exome capture-based CNV discovery is rounded to the nearest successfully captured exon, which represents substantial uncertainty around breakpoint coordinates when comparing to WGS-derived SV coordinates, especially in gene-poor regions of the genome; and
4. The ExAC CNV map reports rare CNV observations per sample but not genotypes, requiring the comparison of site frequencies rather than allele frequencies.

Furthermore, there are considerable technical differences in realistic quality benchmark expectations between exome-based and WGS-based CNV discovery. Specifically:

5. The sensitivity of ExAC compared to SNP microarray data was ~60% for CNVs covering ≥10 microarray probes (a threshold commonly used in microarray CNV analyses; see Supplementary Figure 1 from Ruderfer *et al.* for these data). This sensitivity—while encouraging for exome sequencing published in 2016—is dramatically different from the sensitivity of 97.1% versus microarray CNV calls calculated for gnomAD-SV (see Supplementary Table 4 in this manuscript).
6. The specificity of the CNV calling method used in ExAC, known as XHMM, compares favorably to other exome-based CNV methods (*e.g.*, as in Sadedin *et al.*, *GigaScience*, 2018 or Kumar *et al.*, *Genome Research*, 2019), but is still markedly below performance capable from WGS as demonstrated in this manuscript (for example, Kumar *et al.* demonstrate specificity of 0.16 - 0.36 for XHMM on independent benchmarking data for all but the largest CNVs [> 500kb]). By comparison, we estimate the precision of gnomAD-SV to be in the range of 0.9 - 0.95 based on the seven independent quality assessments performed in this study (see Supplementary Table 4).

Therefore, even though we cannot perform a direct comparison without invoking the slew of caveats above, we fully expect WGS-derived SV callsets like gnomAD-SV to outperform exome-based CNV calling in both sensitivity and specificity due to the substantial technical differences between exome sequencing and WGS.

Regarding the integration of SNVs/indels and SVs, we fully agree that such analyses are a critical area of interest for the human genetics community. However, a combined analysis of all SNVs, indels, and SVs was initially discussed and determined to be outside of the scope of this study; rather, we intended to present an SV resource with a focus on the genome biology, functional consequences, and medical relevance of SVs in the general population. This is very much a goal of future releases of gnomAD, where we will have a direct correspondence of all variant classes jointly analyzed in the same individuals in a much larger dataset, in addition to methods currently in development for statistical phasing of SVs & SNVs to permit analyses of compound heterozygous mutations. Nevertheless, we agree that there are certain analyses that are tractable and within the current study as structured, including those suggested here and by Referee #2 above. We have performed additional analyses as enumerated below:

1. As described above in response to Referee #2, our revised submission now includes a systematic analysis of linkage disequilibrium (LD) between all common SVs and SNVs/indels discovered in European and African/African-American samples passing all

quality control filters in both this study and Karczewski et al. These analyses were fruitful, confirming the accuracy of our SV genotypes on the basis of strong average LD with nearby SNVs/indels (median peak R^2=0.85) and identifying 15,634 common SVs tagged by at least one SNV or indel in strong LD (R^2≥0.8), which can be imputed into future studies without directly ascertaining the SVs themselves. We now described these new LD analyses in our revised manuscript on lines 148 and 341-342, in Extended Data Figure 2c, and in Supplementary Figure 7.

2. Building on the aforementioned LD analyses, we have also intersected the common SVs well tagged by SNVs/indels with an omnibus of human trait-associated SNPs from the union of the NHGRI-EBI GWAS catalogue and the phenome-wide association results in the UK BioBank from the Neale Lab. These new analyses identified 2,307 common SVs strongly tagged by common SNPs at loci reported to be associated with one or more human phenotypes. We were able to identify previously proposed causal SVs tagged by GWAS signals, including common gene deletions in nephropathies and psoriasis, and we also found intriguing candidate SVs tagging as-of-yet unexplained GWAS loci, such as an intronic Alu deletion of a thyroid enhancer at a hypothyroidism GWAS locus. These results are now described in a dedicated paragraph in the main text (lines 337-351), as well as in Extended Data Figure 7 and Supplementary Table 5.
3. Separately, as described in response to Referee #2 (see above), we have investigated the possibility of a genetic DNA repair defect in the sample carrying the highly complex rearrangement shown in Figure 6e and Extended Data Figure 8. This involved manual evaluation of all rare pLoF SNVs, indels, and SVs for this sample, and based on this combined analysis we were unable to confirm the genetic basis for any DNA repair defect, which we note in our revised manuscript on line 389.

2. The section on "Relevance to disease association and clinical genetics" is most important as it addresses the potential for gnomAD-SV to enhance human genetic of disease across the world. However, I had the most trouble with the clarity of the writing and conclusions in this section. First, the authors present case-control analyses of 4 disease cohorts, with various levels of filtering on gnomAD-SV a priori. This doesn't seem like a great idea, and certainly not something done as casually as presented here, as differences in the geographic ancestry among the cases, the controls, and the gnomAD samples could easily produce spurious associations. It's probably important to point out that the quantitative filtering performance reported in these analyses (i.e. what's in Extended Data Figure 6) is highly dependent on the platform being used for the case/control (tumor/normal) data - results will differ with WGS data.

We thank the referee for raising their concerns regarding the clarity and interpretation of this section, which was a component of the paper that we too had debated at length among the consortium. In response to these critiques, we have heavily reworked this section of the manuscript and attempted to simplify the conclusions and inferences. The referee raises important considerations for the analyses presented in ED Figure 6, which we regret we did not emphasize in our initial submission. In the interest of avoiding potential confusion and oversimplifying the technical considerations required when integrating population-based

reference datasets into disease association studies, we have removed the CNV filtering analyses as presented in ED Figure 6, and have used that space instead to include an analysis of SVs in strong LD with GWAS loci suggested by Referee #2, which we think provides a useful and robust application of the dataset for end users (manuscript lines 148 and 341-342, and Extended Data Figure 7). We believe this replacement strengthens the manuscript as it increases the diversity of results presented, while still reinforcing applications of gnomAD-SV to disease association studies.

4. Next, the authors dive into a detailed analysis of pathogenic allele frequencies at 51 genomic disorder loci. The estimated genotyping error rate for the entire gnomAD-SV resource is in the range of 4-10%. I expect there is some heterogeneity in this rate depending sequence context, SV type, etc. Given the detailed analysis and interpretation provided for these 51 sites, it would seem useful to have a better sense of the quality of genotyping specifically at these loci. Have the authors carefully inspected the genotyping accuracy for all 51 of these GD regions, e.g. by assessing the raw data underlying these calls?

This is a valuable point, and is a question that will be shared by other readers. We touch upon the extensive manual inspection of these GD loci in response to Referee #1. We agree that we could have provided more information in our initial submission regarding the quality of CNV calls specifically at these GD loci. As described above, we have now performed manual evaluation of the underlying read-depth evidence for over one thousand CNVs in gnomAD-SV, including 697 autosomal CNVs ≥500kb and 326 complex CNV intervals ≥50kb, finding a total of just four CNVs (0.4%; 4/1,023) lacking unambiguous support by the expected read-depth signatures, which confirms the high specificity of our SV discovery & genotyping methods for large CNVs. To further augment the GD analyses in the manuscript, we have included a new Supplementary Figure 20, which provides detailed read depth evidence supporting the CNV calls at all GD loci with at least one predicted carrier in gnomAD-SV compared to the background population of non-carrier samples. This figure has been reproduced below for convenience:

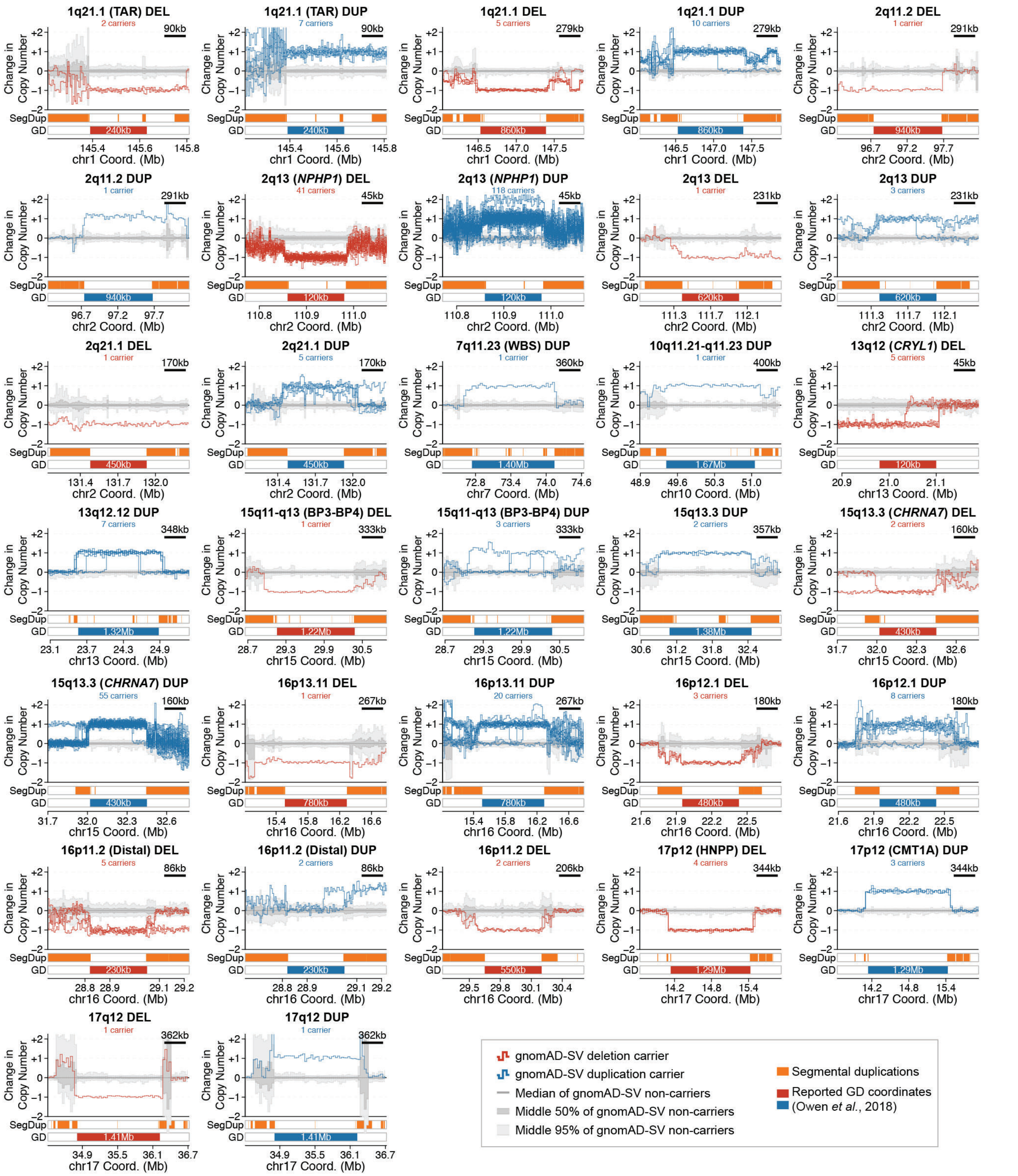
1q21.1 (TAR) DEL
2 carriers
90kb
240kb
chr1 Coord. (Mb)
1q21.1 (TAR) DUP
7 carriers
90kb
240kb
1q21.1 DEL
5 carriers
279kb
860kb
1q21.1 DUP
10 carriers
279kb
860kb
2q11.2 DEL
1 carrier
291kb
940kb
chr2 Coord. (Mb)
2q11.2 DUP
1 carrier
291kb
940kb
2q13 (NPHP1) DEL
41 carriers
45kb
120kb
2q13 (NPHP1) DUP
118 carriers
45kb
120kb
2q13 DEL
1 carrier
231kb
620kb
2q13 DUP
3 carriers
231kb
620kb
2q21.1 DEL
1 carrier
170kb
450kb
2q21.1 DUP
5 carriers
170kb
450kb
7q11.23 (WBS) DUP
1 carrier
360kb
1.40Mb
chr7 Coord. (Mb)
10q11.21-q11.23 DUP
1 carrier
400kb
1.67Mb
chr10 Coord. (Mb)
13q12 (CRYL1) DEL
5 carriers
45kb
120kb
chr13 Coord. (Mb)
13q12.12 DUP
7 carriers
348kb
1.32Mb
15q11-q13 (BP3-BP4) DEL
1 carrier
333kb
1.22Mb
chr15 Coord. (Mb)
15q11-q13 (BP3-BP4) DUP
3 carriers
333kb
1.22Mb
15q13.3 DUP
2 carriers
357kb
1.38Mb
15q13.3 (CHRNA7) DEL
2 carriers
160kb
430kb
15q13.3 (CHRNA7) DUP
55 carriers
160kb
430kb
16p13.11 DEL
1 carrier
267kb
780kb
chr16 Coord. (Mb)
16p13.11 DUP
20 carriers
267kb
780kb
16p12.1 DEL
3 carriers
180kb
480kb
16p12.1 DUP
8 carriers
180kb
480kb
16p11.2 (Distal) DEL
5 carriers
86kb
230kb
16p11.2 (Distal) DUP
2 carriers
86kb
230kb
16p11.2 DEL
2 carriers
206kb
550kb
17p12 (HNPP) DEL
4 carriers
344kb
1.29Mb
chr17 Coord. (Mb)
17p12 (CMT1A) DUP
3 carriers
344kb
1.29Mb
17q12 DEL
1 carrier
362kb
1.41Mb
17q12 DUP
1 carrier
362kb
1.41Mb
Change in Copy Number
SegDup
GD
gnomAD-SV deletion carrier
gnomAD-SV duplication carrier
Median of gnomAD-SV non-carriers
Middle 50% of gnomAD-SV non-carriers
Middle 95% of gnomAD-SV non-carriers
Segmental duplications
Reported GD coordinates (Owen et al., 2018)

5. The authors state that the observed NPHP1 duplication frequency in EAS "caps the credible effect size of NPHP1 duplications in severe diseases, and underscores the value of characterizing putatively disease-associated SVs across diverse populations." This statement is facile and needs further explanation. Have they excluded genotyping error as an explanation for the high frequency NPHP1 duplications in EAS? How do the authors know this (these) NPHP1 allele(s) in EAS is (are) equivalent to those in other populations? What is the cap on the credible effect sizes of NPHP1 duplications dictated by this observation? Have the authors ruled out alternate explanations for this observation that accommodate existing estimates of the effect size of NPHP1 duplications?

We regret the confusion regarding *NPHP1*, which was also raised by Referee #1 (point #17). As we discussed in response to the comment by Referee #1, we have assessed the evidence for NPHP1 duplications, finding consistent read-depth signatures across populations corresponding to previously reported CNV coordinates and strong evidence in favor of our predicted duplication carrier genotypes. Please see the response to Referee #1 point #17 for more information.

Given that these *NPHP1* duplications were a point of contention for two independent referees, we have de-emphasized this result in our revised manuscript, and instead used the space for SV vs. SNV/indel linkage disequilibrium analysis, an integration of SVs into existing GWAS resources, and analyses of noncoding dosage sensitivity.

Supp Fig 12- Would have liked to see more integration with SNV - how many sites in gnomAD appear to be het loF on the basis of SNV data but are actually compound-het LoF when integrated with SV data. Would be even better to annotate this somehow in gnomAD browser.

These are both excellent suggestions and we agree that the integration of the SNV/indel and SV datasets are a high priority for future analyses in gnomAD. As discussed above, these specific analyses will be a focus of the next gnomAD release on much larger datasets. However, this integration is not feasible on the current release due to technical and sample constraints for the current release, and representing variant phase information (even just for pairs of SNVs, let alone SNVs & SVs) in the gnomAD browser across many thousands of samples requires the development of new methods and algorithms. This is an active area of ongoing work for the gnomAD group and we hope one that will yield new insights in a future release.

Supplemental information, pg 23 "Due to the availability of GRCh37-aligned WGS BAM files …" please reword this sentence. Were the BAMS analyzed by Karczewski et al 2019 aligned to a different reference? I would find that surprising since the gnomAD website states "All data are based on GRCh37/hg19."

The referee is correct. All data presented in Karczewski *et al.*, 2019, and in this study, are based on the same reference genome assembly (GRCh37/hg19). We regret this confusion and have removed the reference to the genome assembly build version in the sentence highlighted by the referee to improve clarity.

Line 140 - it would be useful to mention the sequencing depth of gnomAD samples for comparison with 1KG and GTEx.

We agree that sequencing depth is an important covariate when interpreting these comparisons. Thus, we have included median coverage at all points in the manuscript where 1000 Genomes and GTEx results are mentioned (Figure 1 legend and lines 444-448).

Line 210- here is another reason that this mutation rate estimate is likely to be biased: the Watterson estimator is based on the assumption of a neutrally evolving population (i.e. the standard neutral Wright-Fisher model), which clearly does not obtain for SVs observed in a mixture of chromosomes from the diverse populations in gnomAD.

We agree that estimating SV mutation rates is a major outstanding problem in the field. We discuss some particular challenges in the earlier response to Referee #1 (see points #1 and #14), but further elaborate below.

Regarding the Watterson estimator: we acknowledged that it is an imperfect method for estimating mutation rates, and stated this limitation in the main text. Despite these limitations, it is worth noting three points:

1. Our rate of molecularly validated de novo SVs from a previous analysis of 519 quartet families is statistically consistent with the 95% confidence interval of our population-based mutation rate from the Watterson estimator;
2. There is an established precedent for this method being used in prior seminal human SV studies across diverse populations (*e.g.*, Conrad *et al., Nature*, 2010; Mills *et al., Nature*, 2011; Sudmant *et al., Nature*, 2015); and
3. We attempted to control for population structure—one of the largest confounders for this method—by calculating mutation rates for each SV type per population, then reporting the mean across populations.

Given the above, we suspect that these estimates represent imperfect yet informative (and perhaps expected) results for the field, as they are among the first mutation rate estimates derived from population-scale application of deep genome sequencing that can, serve as a comparator to these prior seminal SV studies.

Finally, based on preliminary data from the application of long-read WGS and other emerging technologies to handfuls of human genomes, we expect that the SV mutation rates reported in gnomAD-SV certainly underestimate the true mutation rates and diversity of SVs in humans; however, large-scale applications of these emerging technologies will be required to derive robust mutation rate estimates.

Line 315- "These data estimate that roughly 0.05%…" please rephrase; the humans are doing the estimation here.

Thanks for the suggestion; we have rephrased this sentence accordingly.

Line 340 - "filtering all SVs found in an individual genome versus gnomAD-SV dramatically reduced the number of singleton SVs in that genome to a median of 13". Can the authors please clarify in the text the samples being used here? Are these results based on a "leave one out" type of analysis, where one gnomAD sample is removed from the cohort, SV AFs are re-estimated, and filtering is applied to this one sample? If so, this is probably not a realistic example of how gnomAD-SV will be used. What is more likely is that the clinical case genome will be processed with a different SV calling method, and the false positive rate in the resulting callset will be higher, as the analysis will not benefit from the rigorous QC performed here, with >14K samples of background for setting baselines. Does gnomAD-SV provide an advantage in filtering benign CNVs that overlap protein-coding exons, compared to the existing ExAC CNV map, which is based on a large sample set?

We regret the lack of clarity surrounding the presentation of these analyses in our initial submission. The referee is correct: it was a leave-one-out type of analysis, as conducted through serial downsampling of the gnomAD-SV dataset described in the supplementary methods. The critique that this situation is overly optimistic in many clinical applications is valid, and the true impact of filtering based on gnomAD-SV is highly dependent on the technical details of SV discovery performed in external samples. As such, we have removed this analysis from our revised manuscript, and have replaced it with an integration into existing GWAS databases, which has allowed us to increase the breadth of results and applications of gnomAD-SV presented in this manuscript.

Figure 4a - it's probably incorrect to state that the predicted effect of whole-gene inversion is "No effect", especially given that the singleton proportion for that class could indicate that they are more deletions than pLoF deletions. Possible effects of a whole gene inversion include disruption of cis- and trans-regulation of the gene, leading to ectopic expression or abnormal expression levels across the normal expression.

We agree that this labeling is potentially unclear. The intended meaning was a direct genic effect, excluding potential positional effects. As coding sequence is not disrupted for whole-gene inversions, there would be no predicted direct effect. We have corrected the labeling in Figure 4a to better reflect this.

Figure 4d - I noticed this reference to Supplementary Figure 10 is incorrect; it should be Supp Fig 11. I haven't systematically checked the accuracy of the other figure references.

We thank the referee for bringing this to our attention, and have corrected it in our revised submission.

Figure 5 - panel(C) what do the horizontal dashed lines represent? . panel (D) The authors state that they have "re-estimated ORs for each of the 51 GDs by comparing to the 29,085 DD cases from (c)". First I don't think this statement is coherent, as there appear to be no DD cases explicitly shown in (c) or cited in the caption for (c). Second, the authors should clarify the annotation on the right side -the GD loci within the "0.05%" have a cumulative carrier frequency of 0.05%, I believe, but that is not at all obvious from the diagram or caption.

The horizontal dashed lines in original Figure 5c represented axis breaks. Based on comments from all three referees, we have extensively restructured the final results section of the manuscript (*vis a vis* disease association/medical relevance). The content from the original Figure 5 has now been extensively reworked and largely restricted to Supplementary Information to enable presentation of the GWAS integration and the noncoding dosage sensitivity analyses. The results presented in the original Figure 5 were intended to demonstrate that read depth-based SV discovery from WGS can faithfully capture homology-mediated, clinically relevant large CNVs. However, the population genetics and disease risk conferred by these regions has been the focus of many prior studies, and thus the results we obtained in gnomAD-SV were in line with prior work but not unexpected. We have therefore prioritized new analyses with more novelty (i.e., dosage sensitivity in the noncoding genome) and/or immediate applicability (i.e., SV/SNV LD maps and GWAS integration) in our revised manuscript.

Supplement, pg 44 - section "Estimating SV mutation rates" - the mathematical symbols didn't render properly in the PDF, for instance, one line says: "Where was the number of SV sites observed per population for a given SV class and was the total number of chromosomes analyzed in each population". Clearly "K" and "n" are missing here.

This was a pdf rendering error, and we have ensured the symbols are rendered as appropriate in our revised submission. Thank you for noting this.

The gnomAD-SV downloads (the VCF and bed files) available from the gnomAD website could really benefit from a README.

We thank the referee for this suggestion. Instead of a README, we have drafted & released a blog post explaining many features of gnomAD-SV dataset in more plain-speech detail, which can be viewed here:
https://macarthurlab.org/2019/03/20/structural-variants-in-gnomad/
We think that this detailed blog post is a suitable alternative for a README, and we suspect the web-based format will be more easily accessible for the broad user base of gnomAD. For computational users, each term present in the gnomAD resource is defined in the VCF header.

We have also added extensive documentation to the gnomAD browser to aid in interpretation of each SV class and functional annotation. These can be accessed by clicking the "?" next to various labels or info fields on the gnomAD browser when viewing SV data.

We hope that end-users of gnomAD-SV will find the additional accessibility/help text provided in the browser and the supplementary explanations in the blog post instructive.

Could the authors provide the revised GD OR estimates shown in 5D as a supplemental table?

Certainly; we have now provided Supplementary Table 6, which lists the GD loci evaluated in this study along with carrier statistics for gnomAD and the UK BioBank. We opted against including odds ratios, as we reasoned that it would be facile for other researchers to compute odds ratios for their phenotype of interest given their particular CNV dataset.

Reviewer Reports on the First Revision:

Referee #1:

In the revised version of the manuscript "An open resource of structural variation for medical and population genetics", the authors have addressed most of my concerns and I believe that their computational results are reliable.

My additional comments are listed below:

The authors introduced an improved method (Adjusted Proportion of Singletons; APS) to uniformly quantify selection on SVs in a manner that is similar to the MAPS metrics used for SNVs. This concept is intriguing since MAPS for SNVs is a good metric to evaluate selection of SNVs, which is better-understood than selection on SVs.

Nevertheless, I question how precise APS is in quantifying selection. For example, from Figure 2, APS of paired-duplications-inversions and of insertion-with-insertions-site-deletions are higher than that seen for simpler SVs, such as dispersed duplications. This is strange. Is SV complexity one of the factors for APS calculation? Would an analysis and discussion of SV mechanisms help explain and justify the APS results better?

The authors indicated that "57% of the SVs reported by the 1000 Genomes Project were captured by gnomAD-SV and 14% of SVs reported by gnomAD-SV were also captured in the 1000 Genome Project". I suspect that the main difference would be from rare SVs captured by the gnomAD-SV analysis. It makes me wonder what proportion of the common SVs (Allele Frequencies > 1%) would overlap with the previously reported SVs, if we only consider common SVs (AF > 1%). This may support the reliability of gnomAD-SV results.

So in summary, this paper represents a lot of work and is well-written. However, the results are somewhat expected, isn't it? What is really new and/or unexpected in the findings? What is the impact to the general reader in Nature. I am having difficulties understanding this.

Referee #2:

The authors have revised and improved their manuscript substantially. The quality of the SV resource was substantiated using additional SV-SNP LD analysis for common SVs, inspection of the population distribution of doubleton SVs and using additional benchmarking exercises using the available trio and long-read data sets. The authors could also convincingly show that the VCF variant quality scores are well-calibrated and can be used for post hoc filtering for extracting a high-quality subset of SVs with improved SNP-taggability, long-read confirmation rates and reduced de novo SV fractions. SV breakpoint accuracy was evaluated as suggested. The proposed SV resource is in our view of adequate quality and will have broad applications for population genetics and disease studies.

Referee #3:

I have read carefully through the rebuttal and have no further comments.

Author Rebuttals to First Revision:

RESPONSE TO REFEREES

A structural variation reference for medical and population genetics

Collins*, Brand*, et al.

Responses to Referee #1

In the revised version of the manuscript "An open resource of structural variation for medical and population genetics", the authors have addressed most of my concerns and I believe that their computational results are reliable.

We thank the referee for their additional comments, and for their help in improving the manuscript.

The authors introduced an improved method (Adjusted Proportion of Singletons; APS) to uniformly quantify selection on SVs in a manner that is similar to the MAPS metrics used for SNVs. This concept is intriguing since MAPS for SNVs is a good metric to evaluate selection of SNVs, which is better-understood than selection on SVs.

Thank you, this was indeed our motivation and we hope it will facilitate interpretation of our results relative to our companion paper of SNVs.

Nevertheless, I question how precise APS is in quantifying selection. For example, from Figure 2, APS of paired-duplications-inversions and of insertion-with-insertions-site-deletions are higher than that seen for simpler SVs, such as dispersed duplications. This is strange. Is SV complexity one of the factors for APS calculation? Would an analysis and discussion of SV mechanisms help explain and justify the APS results better?

We agree that this is an interesting result, and SV complexity was intentionally not included in the APS model. Given this, the most parsimonious interpretation is that SV complexity is a contributing factor to the degree of selection acting on SVs. We find the results regarding dispersed duplications to meet expectations, if the model is well calibrated. Dispersed duplications are much less likely to result in predicted loss-of-function (pLoF) than paired-duplication inversions or insertions with insertion-site deletions: just 0.4% of dispersed duplications result in pLoF compared to 22.9% of paired-duplication inversions and 6.6% of insertions with insertion-site deletions. This finding is thus consistent with all observations in our analyses that pLoF SVs experience greater selection across effectively all mutational contexts. We share the referee's interest in the underlying mechanisms driving these patterns of selection, and will actively pursue this question in future releases of gnomAD at much greater scale.

So in summary, this paper represents a lot of work and is well-written. However, the results are somewhat expected, isn't it? What is really new and/or unexpected in the findings? What is the impact to the general reader in Nature. I am having difficulties understanding this.

We believe the impact of the work to be significant across multiple domains of basic research, population genetics, and clinical diagnostic interpretation. Perhaps most importantly, the value of the open resource and its availability in a well curated gnomAD database is likely to be comparable to that of ExAC, gnomAD and the 1000 Genomes Project, to name just a few reference resources.

Responses to Referee #2

The authors have revised and improved their manuscript substantially. The quality of the SV resource was substantiated using additional SV-SNP LD analysis for common SVs, inspection of the population distribution of doubleton SVs and using additional benchmarking exercises using the available trio and long-read data sets. The authors could also convincingly show that the VCF variant quality scores are well-calibrated and can be used for post hoc filtering for extracting a high-quality subset of SVs with improved SNP-taggability, long-read confirmation rates and reduced de novo SV fractions. SV breakpoint accuracy was evaluated as suggested. The proposed SV resource is in our view of adequate quality and will have broad applications for population genetics and disease studies.

We thank the referee for their constructive comments, which have demonstrably improved the manuscript, and for their positive assessment of the potential impact of our study.

Responses to Referee #3

I have read carefully through the rebuttal and have no further comments.

We thank the referee for their useful comments and their help in improving the quality of our manuscript.
